# Oxygen-dependent regulation of F-box proteins in *Toxoplasma gondii* is mediated by Skp1 glycosylation

**DOI:** 10.1016/j.jbc.2024.107801

**Published:** 2024-09-21

**Authors:** Msano N. Mandalasi, Elisabet Gas-Pascual, Carlos Gustavo Baptista, Bowen Deng, Hanke van der Wel, John A.W. Kruijtzer, Geert-Jan Boons, Ira J. Blader, Christopher M. West

**Affiliations:** 1 Department of Biochemistry & Molecular Biology, Center for Tropical and Emerging Global Diseases, Complex Carbohydrate Research Center, University of Georgia, Athens, Georgia, USA; 2 Department of Microbiology & Immunology, University at Buffalo School of Medicine, Buffalo, New York, USA; 3 Chemical Biology & Drug Discovery, Utrecht Institute for Pharmaceutical Sciences, Utrecht University, CG Utrecht, The Netherlands; 4 Department of Chemistry, Complex Carbohydrate Research Center, University of Georgia, Athens, Georgia, USA

**Keywords:** ubiquitin ligase, prolyl hydroxylase, glycosyltransferase, *Toxoplasma*, oxygen-sensing;, hydroxyproline, O-linked glycan, F-box protein

## Abstract

A dynamic proteome is required for cellular adaption to changing environments including levels of O_2_, and the SKP1/CULLIN-1/F-box protein/RBX1 (SCF) family of E3 ubiquitin ligases contributes importantly to proteasome-mediated degradation. We examine, in the apicomplexan parasite *Toxoplasma gondii*, the influence on the interactome of SKP1 by its novel glycan attached to hydroxyproline generated by PHYa, the likely ortholog of the HIFα PHD2 oxygen-sensor of human host cells. Strikingly, the representation of several putative F-box proteins (FBPs) is substantially reduced in PHYaΔ parasites grown in fibroblasts. One, FBXO13, is a predicted lysyl hydroxylase related to the human JmjD6 oncogene except for its F-box domain. The abundance of FBXO13, epitope-tagged at its genetic locus, was reduced in PHYaΔ parasites thus explaining its diminished presence in the SKP1 interactome. A similar effect was observed for FBXO14, a cytoplasmic protein of unknown function that may have co-evolved with PHYa in apicomplexans. Similar findings in glycosylation-mutant cells, rescue by proteasomal inhibitors, and unchanged transcript levels suggested the involvement of the SCF in their degradation. The effect was selective because FBXO1 was not affected by loss of PHYa. These findings are physiologically significant because the effects were phenocopied in parasites reared at 0.5% O_2_. Modest impact on steady-state SKP1 modification levels suggests that effects are mediated during a lag phase in hydroxylation of nascent SKP1. The dependence of FBP abundance on O_2_-dependent SKP1 modification likely contributes to the reduced virulence of PHYaΔ parasites owing to impaired ability to sense O_2_ as an environmental signal.

*Toxoplasma gondii* is a highly successful intracellular apicomplexan parasite of warm-blooded animals. It typically infects a host orally, disseminates *via* the vascular system possibly *via* intracellular transport, and persists as slow-growing cysts in the central nervous system and muscles, where further growth is suppressed by the immune system (1). Uniquely in the gut of felines, the parasite may also engage in a sexual cycle that generates dormant oocysts that pass into the environment (2). All these transitions must be guided by external cues that are sensed and used by the parasite to modify its proteome to support new activities in new anatomical locations. One process that governs the proteome is the targeted removal of specific proteins, and a primary route involves tagging with polyubiquitin chains that are in turn recognized by factors that deliver the protein to the 26S-proteasome to be degraded into amino acids (3). The SKP1/cullin-1/F-box protein/Rbx1 (SCF) family of E3 ubiquitin (Ub) ligases contributes importantly to protein degradation because of the multitude of F-box proteins (FBPs) encoded by most eukaryotic genomes (4, 5). Most FBPs are substrate receptors that are linked to the rest of the complex through their F-box domains to a single adaptor protein, SKP1. The SCF complexes are also referred to as the Cullin-RING-ligase-1 (CRL1) family. CRLs, of which there are three types in *Toxoplasma* owing to different cullin isoforms (6), are in general regulated by the neddylation of the cullin subunit. Neddylation controls both binding to the complex of a Ub-charged E2 protein that donates Ub to the target, and an FBP/SKP1 pair that presents the target or in some cases is the target (7). An SCF-specific mode of regulation has been identified in the social amoeba *Dictyostelium discoideum*, where O_2_-dependent glycosylation of SKP1 (8) affects the repertoire of associated FBPs (9, 10), which would influence access of FBPs to the SCF complex and therefore the susceptibility of potential targets for degradation. The genes that encode the 6 enzyme activities that modify SKP1 are conserved in selected organisms throughout the protist kingdom (8, 11), and a prior study documented that *Toxoplasma* SKP1 is similarly, though not identically, modified (12).

The multi-step modification of *Toxoplasma* SKP1 begins with O_2_-dependent hydroxylation of a key Pro residue (12) in a disordered region (13) that mediates association with the F-box domain of the FBP. The resulting hydroxyproline serves as an anchor for a series of 5 glycosyltransferase activities (Fig. 1*A*) that assemble a linear glycan (12, 14, 15), which is hypothesized to influence the conformation of SKP1 in a way that promotes interaction with select FBPs (15, 16), and also to inhibit dimerization which may sequester SKP1 from complexing with FBPs (17, 18). Evidence indicates that this mechanism constitutes an O_2_-sensing pathway used by *Dictyostelium* to assess its position in the soil as part of its decision-making process to form fruiting bodies at the soil surface (8). This post-translational modification appears to be dedicated to SKP1 and the finding that proteasomal inhibition reverses the effect of the mutational blockade of SKP1 modification (10) supports the model that this O_2_-dependent mechanism acts, as expected, *via* its polyubiquitination activity.

**Figure 1:**
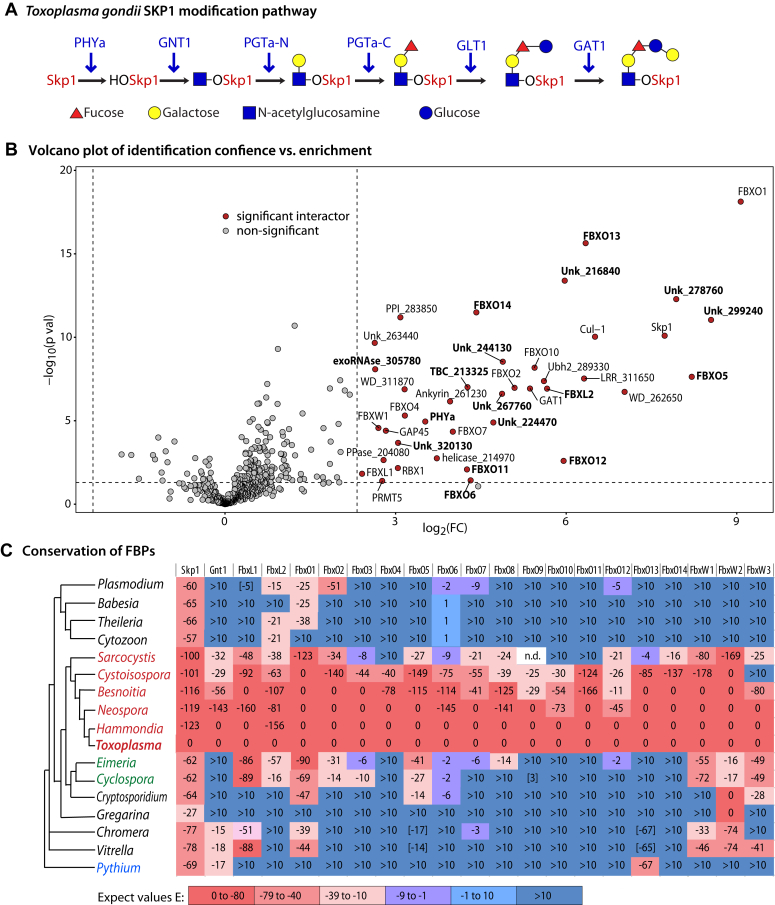
**Skp1 glycosylation pathway and summary of Skp1-SF interactors.***A*, Skp1 is modified at Pro154 by a specific O_2_-dependent prolyl 4-hydroxylase, and 5 glycosyltransferase reactions mediated by 4 proteins that generate the linear pentasaccharide glycan on the resulting hydroxyproline residue. *B*, Volcano plot of significant interactors based on co-IPs targeting SF-tagged SKP1 (SKP1-SF) with anti-FLAG mAb M2, and analysis of peptides generated from the co-IPs using mass spectrometry. The vertical dashed line indicates a 5-fold enrichment of proteins found in the tagged vs. untagged strain, and the horizontal dashed line indicates statistical significance of the fold-change of *p* < 0.05. Candidate protein hits that satisfy these criteria (upper right quadrant) are in red. Proteins that were enriched >2-fold (*p* < 0.05) in parental (RHΔΔ) vs. PHYaΔ parasites are in bold. Only proteins identified with ≥2 peptides and an FDR <5%, detected were plotted, except for FBXL1, FBXW1 and FBXO4 (see Table 1). *C*, Taxonomic distribution of putative *Toxoplasma* F-box proteins. Predicted FBPs described in Table 2 were used to search for homologs of the full-length *Toxoplasma* sequences among apicomplexans, two closely related alveolates (*Chromera velia* and *Vitrella brassicaformis*), and a more distantly related stramenopile (*Pythium ultimum*) using the BLASTp algorithm. Incomplete sequences were reconstructed from genome sequences where possible (Table S3). Sequence alignments were used to confirm the apparent conservation of F-box-like domains. Rows of the heat map that describe homology based on expected values are organized according to the phylogenetic cladogram described at the left. The first 2 columns show SKP1 and conservation of its modification pathway as represented by its first glycosyltransferase, GNT1. Expect values that are bracketed indicate the absence of evidence for an F-box sequence. Sarcocystid coccidians are named in red; non-sarcocystid coccidians are in green, and *Pythium* is in blue. n.d., refers to a sequence reconstructed from genomic data that did not receive an Expect value. See Tables S2 and S3 for sequence identifiers.

Previous studies predicted 18 FBPs in *Toxoplasma*, with four validated in a preliminary characterization of the TgSKP1 interactome (19). Here we explored the interactome of SKP1 at a greater depth and compared it to the interactome in mutant cells that lack the SKP1 prolyl hydroxylase (PHYa), which evidence suggests is the protist ortholog of human PHD2 that regulates transcriptional responses to O_2_ *via* a CRL2-dependent mechanism (20). We find that the SKP1-interactome of PHYaΔ cells, chosen to simulate low O_2_, is markedly deficient in several FBPs. To explore the basis of these deficiencies, a detailed analysis was performed for three FBPs: FBXO1, an early component of the daughter cell scaffold (19), FBXO13, a likely lysyl hydroxylase related to human JmjD6 (21, 22) that appears to have an O_2_-sensing role in *Dictyostelium* (10), and FBXO14, which is found only in apicomplexans that also harbor SKP1 modifications genes. Significantly, the two FBPs whose representation was reduced in the SKP1 interactome in the absence of PHYa were also reduced in total amount in the cell likely due to a proteasome-dependent mechanism. Similar findings on O_2_-deprived parasites indicate that PHYa is a physiological regulator of the SCF complex that influences the proteome in a protist-specific manner.

## Results

### Characterization of the Skp1 interactome

The SKP1 interactome was previously examined in the type 1 RH strain using either a co-immunoprecipitation (co-IP) strategy based on an affinity-purified anti-TgSKP1 antiserum, or by using an anti-FLAG antibody for a strain in which the Skp1 locus was modified to append an SF-tag (FLAG tag) at the C-terminus of SKP1 (19). To examine the role of SKP1’s posttranslational modifications, these studies were expanded and repeated in matched SKP1-SF expressing strains, including RHΔhxgprtΔku80 (parental control), KU80ΔPHYaΔ, KU80ΔGNT1Δ, and KU80ΔPGTaΔ strains. For convenience in this report, the parental normal strain is referred to as RHΔΔ whether or not one of its genes has been edited for epitope tagging, and derived deletion strains are simply referred to by the genes deleted (*e.g.*, PHYaΔ). In addition, we expanded the search to compare intracellular and spontaneously egressed extracellular parasites and optimized our magnetic bead pull-down strategy to reduce non-specific binding and increase the depth of coverage.

**Figure 2:**
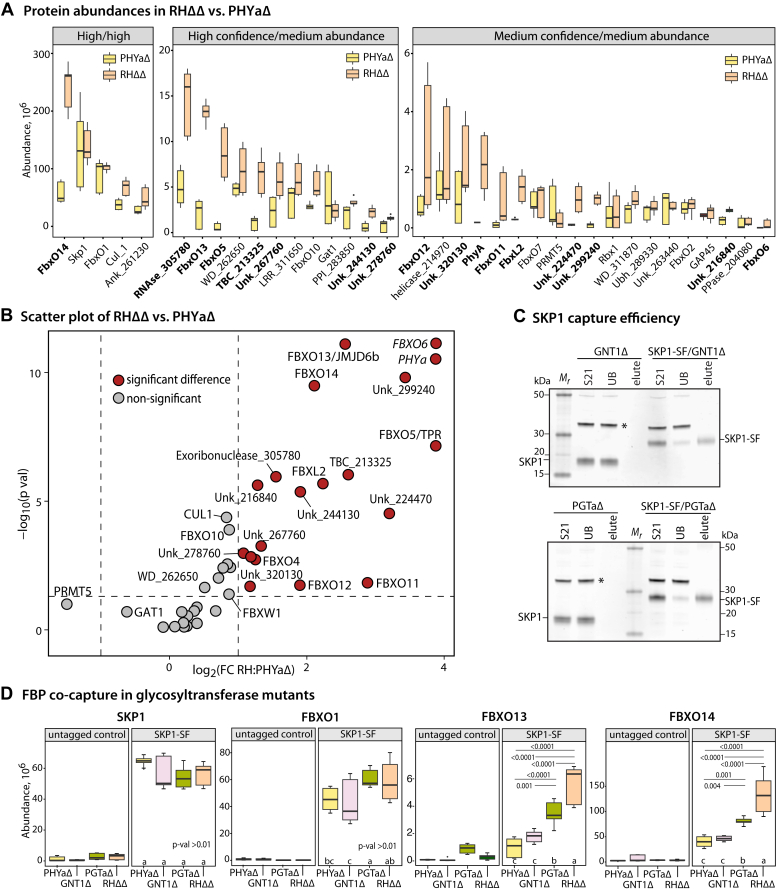
**Comparison of SKP1-SF interactors between parental and SKP1 modification mutants.***A*, abundance boxplot representation of confident SKP1-SF interactors identified in Fig. 1*B* in normal (RHΔΔ, in peach) parasites, in comparison with PHYaΔ (in yellow) parasites, classified by confidence (high<1% FDR; medium <5% FDR) and abundance level. See Fig. S1 for data from co-IPs of control un-tagged strains), and Table 1 for summary. *B*, scatter plot of the data in panel A, comparing log_2_ of the fold change in abundance in RHΔΔ (normal) vs. PHYaΔ cells, against statistical significance of the difference (ordinate). Note that FBXO6 and PHYa were arbitrarily placed in the upper right-hand corner because they were not detected in PHYaΔ extracts. *C* and *D*, SKP1 was additionally SF-tagged in GNT1Δ and PGTaΔ parasites. Spontaneously partially lysed-out parasites from these strains were subjected to co-IP with anti-FLAG mAb M2 as above. *C*, Representative Western blot analyses of co-IP fractions (S21, soluble input fraction; UB, unbound fraction; elute, SDS eluate) from these and their corresponding untagged strains are shown. *D*, relative abundances, based on nLC/MS analyses of these co-IPs as above, of FBXO1, FBXO13, and FBXO14 as well as SKP1, from the 4 (RHΔΔ, PHYaΔ, GNT1Δ, and PGTaΔ) SKP1-SF tagged strains and their corresponding untagged counterparts are plotted. Boxplots represent expression values for 3 independent biological replicates, each with 3 technical replicates, with the whiskers representing the lowest and the highest values of the dataset. Boxes represent the two middle quartiles and the line within the box represents the median. *p*-values for significant differences ≤0.01 are shown. Data were analyzed with 2-way ANOVA with a *p*-value <0.01; there were no statistically significant differences within the SKP1 and FBXO1 groups.

Criteria for classification as a *bona fide* interactor included: (i) detection in anti-FLAG SKP1-SF co-IPs at a level ≥5-fold greater level than in parallel co-IPs of untagged strains, with statistical p-val<0.05, (ii) detection in at least 2 of the 3 biological replicates, and (iii) at least two peptides detected at an FDR of ≤5%. These criteria excluded all known intramitochondrial, intra-apicoplast, or secretory proteins that commonly contaminate co-IPs targeting nucleocytoplasmic proteins. As previously suggested (19), independent analyses indicated no differences between intracellular and extracellular parasites. Thus the data were pooled and summarized as a volcano plot (Fig. 1*B*) that represents the enrichment in tagged vs. untagged strains (abscissa) and the statistical significance for the fold-change (ordinate axis). By analyzing all samples together, 35 proteins were classified as potential high-confidence SKP1 interactors, about double the number of our previous study (19). As described in Table 1, 16 and 19 of these were high (FDR <1%), and medium (1%<FDR<5%) confidence assignments, respectively. Relaxing FDR to <10% confidence, 3 additional predicted FBPs, FBXL1, FBXW1, and FBX04, were identified. The specific data are represented in Fig. S1. The list included 14 potential FBPs (including low-confidence hits), two SKP1 modification enzymes (PHYa and GAT1), and 20 interactors of unknown significance, some of which might be cryptic FBPs with divergent F-box sequences. The apparent relative abundance of steady-state interactors, as inferred from peptide spectral counting, ranged over 200-fold (see Fig. 2*A*). However, this comparison is biased by the number of peptides detected, the number of peptides in the protein, and the efficiency of their detection.

**Table 1:** Interactome of *Toxoplasma* SKP1-SF

The list of interactors included, as expected, subunits of the SCF complex, Cul1, and Rbx1, suggesting that some of the complexes were poised to be active in polyubiquitination. Of the 18 previously predicted FBPs, 10 were detected in the SKP1-SF interactome at high or medium confidence, with 3 more detected at relatively low confidence. Because F-box domain prediction is confounded by extensive degeneracy, the sequences of the remaining interactors were combed for evidence of F-box-like sequences. This search netted an additional predicted FBP (sequence shown in Fig. S2), raising the total to 14 of the 19 predicted FBPs detected to varied degrees of confidence in the SKP1 interactome. 13 of the 16 interactors detected in our previous study (19) were confirmed in this more exhaustive study. Of the missing three, TGGT1_245720 and TGGT1_226850 were detected but did not exceed the ≥5-fold enrichment over control samples and TGGT1_276180 was not seen. Characteristics of currently predicted *Toxoplasma* FBPs, including fitness scores in cell culture (23), molecular weight, detection in extracellular or intracellular parasites, and dependence on PHYa (see below) are summarized in Table 2. Failure to detect the remaining five predicted FBPs was potentially due to expression levels in tachyzoites below the sensitivity of the method. In addition, we do not exclude the possibility that others of the interactors have cryptic F-box sequences.

**Table 2:** Predicted *Toxoplasma* F-box proteins

Name	Gene ID	Motif	Fitness score (23)	TGGT1		SKP1 co-IP (all)	Detected in ref. (19)	PHYa effect	RH:PHYa ratio
				aa	kDa				
FBXL1	TGGT1_262530	LRR	−0.18	1013	113	Yes^a^		N	
FBXL2	TGGT1_313200	LRR	−3.85	843	91	Yes	Yes	Y	4.7
FBXO1	TGGT1_310930		−3.02	808	87.5	Yes	Yes	N	
FBXO2	TGGT1_215210		0.49	683	75	Yes		N	
FBXO3	TGGT1_225900		−2.31	1461	161	n.d.			
FBXO4	TGGT1_228380		1.18	1726	187	Yes^a^		Y	2.4
FBXO5	TGGT1_243750	TPR,CAAx	−1.7	868	96	Yes		Y	15
FBXO6	TGGT1_258900		0.31	577	63	Yes		Y	>100
FBXO7	TGGT1_275780		−1.4	979	105	Yes		N	
FBXO8	TGGT1_305630		−2.2	2165	234	n.d.			
FBXO9	TGGT1_359350		−0.45	3034	331	n.d.			
FBXO10	TGGT1_215620		−0.68	1958	207	Yes	Yes	N	
FBXO11	TGGT1_203040		1.55	1824	198	Yes		Y	7.4
FBXO12	TGGT1_278815	Leu zipper	−1.59	2421	264	Yes		Y	3.7
FBXO13	TGGT1_283890	JmjC	−3.04	739	81	Yes	Yes	Y	5.9
FBXO14	TGGT1_259880		1.34	1034	117	Yes	Yes^b^	Y	4.3
FBXW1	TGGT1_261370	WD40	−2.9	1618	177	Yes^a^		N	
FBXW2	TGGT1_299230	WD40	−0.47	1101	122	n.d.			
FBXW3	TGGT1_310910	WD40	−2.79	3679	398	n.d.			

A previously presented list of predicted *Toxoplasma* FBPs (19) is updated by the inclusion of an additional candidate found in the Skp1 interactome described in Table 1 and data regarding dependence on PHYa. Predicted characteristics are listed as follows: other domains (including putative leucine rich repeats (LRR), WD40-repeat or tetratricopeptide-repeat (TPR) substrate receptor domains), fitness score from a genome-wide CRISPR/Cas9 screen (23), *M*_r_, presence detected in the Skp1 interactomes of intracellular (IC) or spontaneously emerged (EC) parasites, and effect of the presence of PHYa. n.d., not detected.

a Low confidence.

b Not noted as an FBP in ref. (19).

To assess the conservation of *Toxoplasma* FBPs, a sequence-based analysis of their evolution within apicomplexans and related protists suggests a surprising lack of evidence for conservation outside of the sarcocystid coccidian group (Fig. 1*C*). This might mean that sarcocystid coccidians are especially rich in FBPs or that extreme sequence divergence masks functional conservation. However, the apparent coevolution with the SKP1 modification pathway, as represented by the SKP1 αGlcNAc transferase shown in column 2, also suggests a functional interdependence such that the activities of these FBPs are influenced by SKP1 glycosylation. The correlation is especially consistent for FBXO4, FBX09, FBXO10, FBXO11, FBXO13 and FBXO14. However, the correlation does not appear to extend to the apicomplexan progenitors *Chromera* and *Vitrella*, which possess the pathway genes, suggesting that the proposed FBP dependence on Skp1 glycosylation emerged later in apicomplexan evolution. Further studies of the repertoire of FBPs in other apicomplexans are warranted to validate a functional correlation, but this will require experimental studies owing to the difficulty in detecting FBPs based on sequence alone. Only FBXO13 can be confidently considered to be widely distributed among protists, based on experimental studies in the amoebozoan *Dictyostelium* (10).

### PHYa dependence of the SKP1 interactome

A parallel analysis of the SKP1-SF interactome of a strain whose PHYa gene was disrupted (PHYaΔ) was performed to assess the biochemical consequences of SKP1 modification. Data from three biological replicates, with three technical replicates each, were analyzed for statistical significance. As quantitated in Fig. 2*A* and Table 1, and displayed graphically in Fig. 2*B*, 15 high- or medium-confidence interactors exhibited statistically significant and ≥2-fold increased representation in the SKP1 interactome of normal (*i.e.*, SF-tagged RHΔΔ, or simply RHΔΔ) vs. PHYaΔ parasites, with 2 proteins not observed at all in PHYaΔ parasites, namely PHYa itself and FBXO6. Seven of these interactors, FBXO5, FBXO11, FBXO12, FBXO13, FBXO14, FBXL2, as well as FBXO6, were classified as FBPs. The role of PHYa appears to be selective for increased steady-state interaction for some but not all FBP-like proteins, with no effect observed for the previously described FBXO1, which is involved in daughter cell scaffold function during parasite replication (19), or FBXO2, FBXO7, and FBXO10. The significance of increased interactions with the seven other proteins, some of which might be cryptic FBPs or co-factors, remains to be investigated. Effects on the SKP1 interactome were not evidently correlated with SKP1 abundance, presence of a predicted substrate receptor domain, or F-box sequence characteristics. Notably, no interactors exhibited statistically significant decreased representation. This observation parallels findings made on the interactome of SKP1 from *Dictyostelium* (9, 10), where the representation of proteins was only increased in PHYa^+^ cells compared to that of PHYaΔ cells. No significant effect of modification on interaction with Cul1 or Rbx1 was detected, suggesting that hydroxylation does not affect association of FBP/SKP1 subcomplexes with Cul1 as an active E3 Ub ligase, as also observed in *Dictyostelium* (10).

### Dependence of high abundance interactors on SKP1 glycosylation

After hydroxylation of Pro154 by PHYa, Hyp154 can be modified by 5 glycosylation reactions to generate the complete glycan (Fig. 1*A*). To explore the role of glycosylation *per se*, the interactome of SKP1-SF was examined in strains whose GNT1 or PGTa genes were disrupted. Western blot analysis of the anti-FLAG co-IPs confirmed efficient capture of SKP1-SF (Fig. 2*C*). Based on proteomics analysis of SKP1-SF co-IPs, similar levels of SKP1-SF were found in parental, PHYaΔ, GNT1Δ, and PGTaΔ parasites (Fig. 2*D*). Among the three most abundant FBP-like SKP1-SF interactors, FBXO13 and FBXO14, which were underrepresented in PHYaΔ, were also underrepresented in the SKP1-SF interactomes of GNT1Δ and PGTaΔ cells relative to RHΔΔ (normal) cells. FBXO13 and FBXO14 were as underrepresented in GNT1Δ cells as in PHYaΔ cells, suggesting that prolyl hydroxylation, which occurred normally in GNT1Δ cells, did not alone explain why normal cells were different. Interestingly, an intermediate representation (between PHYaΔ and RHΔΔ) in the interactomes of PGTaΔ cells was observed, suggesting that successive steps of SKP1 modification lead to their increased representation in the SKP1-SF interactome. This trend was statistically significant and parallels a similar finding for FbxwD in *Dictyostelium* (9). An incremental contribution of successive monosaccharide modifications could help explain the evolutionary origin of this complex pathway. In contrast, the representation of FBXO1, which was not significantly affected by the expression of PHYa, was also not affected by the absence of GNT1 or PGTa. Since the specificity of GNT1 and PGTa for SKP1 is better established than the specificity of PHYa, the results reinforce the inference that the role of PHYa is mediated *via* SKP1 rather than an unknown alternative target.

### FBXO13 and FBXO14 are F-box proteins

**Figure 3:**
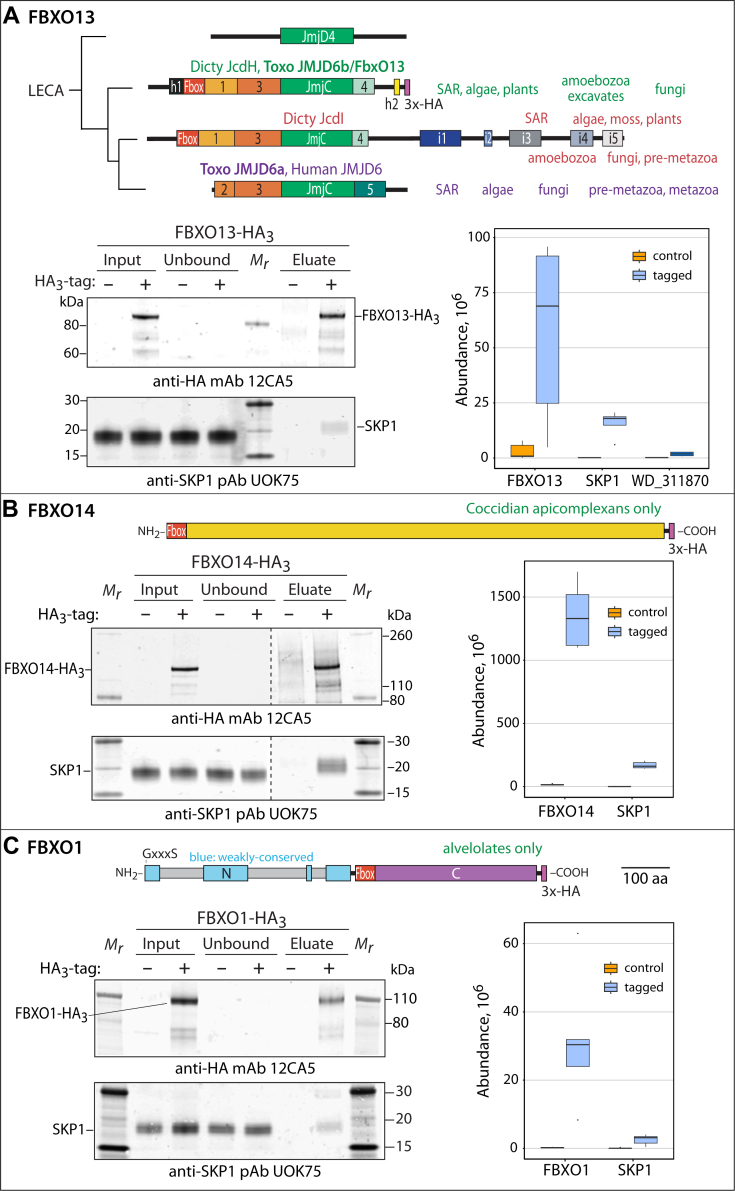
**Interaction of FBXO1, FBXO13, and FBXO14 with SKP1.** The genomic loci of FBXO13, FBXO14 and FBXO1 were edited (Figs. S3, S6 and S8) to modify the protein C-termini with an HA_3_-epitope tag (Figs. S4, S7 and S9). *A*, Domain diagram of *Toxoplasma* JMJD6b, referred to here as FBXO13, and a simplified phylogram (from ref. 10) of its evolutionary relationship with homologs together with a summary of their taxonomic distribution. A yeast representative of the JMJD4 lineage serves as an outgroup; LECA refers to the last eukaryotic common ancestor. The tagged and parental (RHΔΔ) strains were lysed in the presence of a non-ionic detergent and incubated with anti-HA mAb 12CA5 covalently crosslinked to protein A/G magnetic beads. Proteins before and after co-IP were analyzed by SDS-PAGE and Western blotting using mAb 12CA5 for FBXO13-HA_3_, or affinity-purified anti-SKP1 pAb UOK75 for SKP1. Input, unbound, and eluted (with high pH) samples, representing the same fraction of the original extract, were analyzed in parallel. Similarly, proteins were eluted from the beads at high pH and their tryptic peptides were quantitated by mass spectrometry. All proteins that were detected at an FDR of <5%, with ≥2 peptides, and >5-fold enriched over the untagged control strain, are shown. *B*, Domain diagram of FBXO14, which is found only in *Toxoplasma* and closely related species (Fig. 1*C*). Interactors of FBXO14-HA_3_ were analyzed as in panel A. Dashed lines separate different Western blots. *C*, Domain diagram of FBXO1, whose homologs are more broadly distributed beyond apicomplexans to include alveolates. Interactors of FBXO1-HA_3_ were analyzed as in panel A.

FBXO13 is encoded by TGGT1_283890 and annotated in ToxoDB as histone lysine demethylase JMJD6b. Based on a previous phylogenetic analysis (10) and summarized in Fig. 3*A*, *Toxoplasma* FBXO13 belongs to a conserved lineage of enzymes that is found throughout protists and plants. These enzymes all possess an F-box-like sequence which was confirmed in *Dictyostelium* (10). *Toxoplasma* also expresses the paralogous JMJD6a, which lacks an F-box motif and belongs to a separate lineage that is also prevalent in protists and animals. FBXO13 is predicted to be encoded by 8 exons (Fig. S3), and a genome-wide CRISPR screen in a fibroblast monolayer growth setting yielded a fitness score of −3.04 (23), suggesting essentiality. To enable confirmation of its association with SKP1 by reciprocal co-IP, its coding region was genomically edited to encode a C-terminal HA_3_ epitope tag in RHΔΔ cells (Fig. S4). Clonal isolates of RHΔΔ tachyzoites expressed a novel HA-tagged protein with an apparent *M*_*r*_ of 96,000 and minor bands with higher mobility, based on Western blot detection with mAb 12CA5 (Fig. 3*A*). The variation from the predicted *M*_*r*_ of 85,629 (HA_3_-tagged version) is likely due to anomalous migration during SDS-PAGE, though unknown posttranslational modifications cannot be excluded.

To test its interaction with SKP1, extracts of the tagged strain and the untagged parental strain as a control were subjected to co-IP using anti-HA covalently coupled to magnetic beads. As shown in Fig. 3*A*, the great majority of FBXO13-HA_3_ was captured and eluted, and the eluted fraction was accompanied by a minor fraction of SKP1 in the extract, as expected because FBXO13 is only one of many FBPs. As an independent approach, two separate FBXO13-HA_3_ co-IPs were analyzed after elution and digestion with endo Lys-C and trypsin, using mass spectrometry as for the SKP1-SF co-IPs. The abundance of FBXO13 peptides detected (Fig. S5*B*) strongly supported the predicted protein model. As shown in Fig. 3*A* (right panel), SKP1 was the most abundant interactor detected at high confidence, using criteria of <1% FDR, ≥2 peptides detected, and >5-fold enriched in the tagged vs. untagged strain. The interaction between FBXO13 and SKP1 is likely to direct, because FBXO13 contains a sequence (Fig. S2) that is homologous to a sequence in JcdI (Fbxo13) from *D. discoideum* that was demonstrated to be an F-box based on mutagenesis (10). Of the other >600 proteins detected in this co-IP, the only other protein exceeding these criteria was a WD40-repeat-containing protein (TGGT1_311870) also found in the SKP1 interactome (Table 1).

FBXO14 is encoded by TGGT1_259880 (ToxoDB) and annotated in ToxoDB as a protein of unknown function. It is predicted to be encoded by a single exon and the HA_3_-tagged version has a predicted *M*_*r*_ of 121,303 (Fig. 3*B*). It has a cell culture fitness score from a CRISPR screen of 1.04, indicating dispensability during monolayer growth conditions. Its genomic locus was C-terminally tagged as above and clonal isolates yielded a novel band with an apparent *M*_*r*_ of 126,000 (Fig. 3*B*). The validity of this gene model was verified by peptides retrieved after immunoprecipitation of FBXO14-HA (Fig. S5).

Analysis of the FBXO14-HA_3_ interactome confirmed the expected presence of SKP1 based on Western blotting (Fig. 3*B*). This result was confirmed by proteomics analysis of the co-IPs by mass spectrometry, where only FBXO14 and SKP1 were detected above the background of >700 proteins also detected in the control co-IP from untagged cells. Although not experimentally confirmed, FBXO14 possesses a candidate F-box sequence (Fig. S6) at its N-terminus that is highly conserved among sarcocystid apicomplexans, suggesting that its interaction with SKP1 is also direct.

FbxO1 is partially conserved throughout the alveolate group, which includes apicomplexans, but not elsewhere (Fig. 1*C*), and plays an early role in the specialized form of cell division known as endodyogeny in *Toxoplasma* (19). FBXO1 was similarly C-terminally HA_3_-tagged in RHΔΔ cells (Figs. S8 and S9), resulting in a novel anti-HA reactive band with an apparent *M*_*r*_ of 102,000, compared to an expected value of 91,059 (with HA_3_ tag). Confirming a previous study (19), SKP1 robustly co-IPed with FBXO1-HA_3_ based on Western blotting (Fig. 3*C*). Proteomics analysis of the co-IPs confirmed the interaction with SKP1 but failed to identify other significant interactors, whether FBXO1 was HA_3_-tagged at its C-terminus (Fig. 3*C*) or its N-terminus in a Tati strain (not shown).

Notably, Cul1 (TGGT1_289310) was not detected in the interactomes of these three FBPs, in contrast to its abundance in the interactome of FBXL2-HA_3_ (24). Thus these FBPs were not detected as active E3 Ub ligases. Furthermore, subunits of the COP9 signalosome were not detected in these FBP interactomes, also in contrast to that of FBXL2-HA_3_ (24), indicating differential regulation.

### Expression of FBXO13, FBXO14, and FBXO1 in Skp1 modification mutants

**Figure 4:**
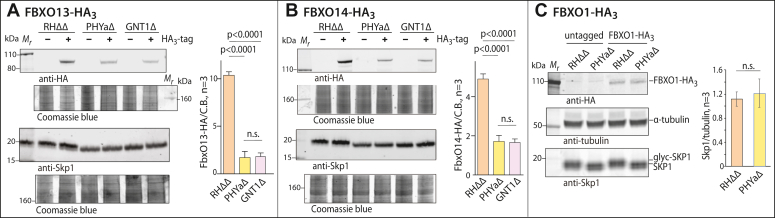
**Dependence of FBXO13-HA**_**3**_**, FBXO14-HA**_**3,**_**and FBXO1-HA**_**3**_**abundance on PHYa and GNT1.** FBXO13, FBXO14, and FBXO1 were HA_3_-tagged at their genetic loci in the indicated Skp1 modification mutant strains, and their levels were compared with their levels in RHΔΔ. *A*, Western blot analysis of FBXO13-HA_3_ and SKP1 levels in SDS-solubilized whole cell lysates from RHΔΔ (parental), PHYaΔ, and GNT1Δ strains that were mechanically lysed out of hTERT cells. The bar graph shows the average ± S.D, based on densitometry normalized to Coomassie blue stained post-blotted gels from 3 independent trials. Mobility differences for SKP1 are due to differences in glycosylation. *B*, FBXO14-HA_3_ levels were analyzed as in panel A. *C*, FBXO1-HA_3_ levels were analyzed as in panel A except that levels were quantitated by densitometry ratioed to α-tubulin as a loading control. Samples were not reduced and alkylated, explaining the more dispersed migration of SKP1. Significance was tested using an unpaired *t* test.

To explore the reason for their under-representation in the SKP1 interactomes of PHYaΔ and GNT1Δ parasites, FBXO13 and FBXO14 were also epitope-tagged in PHYaΔ and GNT1Δ parasites and, for comparison, FBXO1 was tagged in PHYaΔ parasites. Western blot analysis of whole cell lysates from spontaneously lysed extracellular parasites showed that FBXO13-HA_3_ levels were considerably reduced, over 3-fold, in PHYaΔ relative to RHΔΔ (normal) cells (Fig. 4*A*), which likely explains its reduced abundance in the SKP1 interactome of PHYaΔ. A similarly reduced level was seen in GNT1Δ parasites, reinforcing the importance of glycosylation of SKP1. This finding is significant because it strongly indicates that the role of PHYa involves SKP1 since the evidence that GNT1 is specific for SKP1 is stronger than the evidence that PHYa is dedicated to SKP1 (12). A similar though more modest >2-fold reduction was observed for FBXO14-HA_3_ in both PHYaΔ and GNT1Δ parasites (Fig. 4*B*). In contrast, levels of FBXO1-HA_3_ were not sensitive to SKP1’s modification status by PHYa (Fig. 4*C*), consistent with its similar representations in the interactomes of SKP1-SF from PHYaΔ and RHΔΔ cells. Given the involvement of SKP1 in E3(SCF) mediated ubiquitination, we hypothesized that FBXO13 and FBXO14 levels are influenced by proteasome-mediated degradation.

### FBXO13 and FBXO14 abundances appear to be regulated by protein turnover

**Figure 5:**
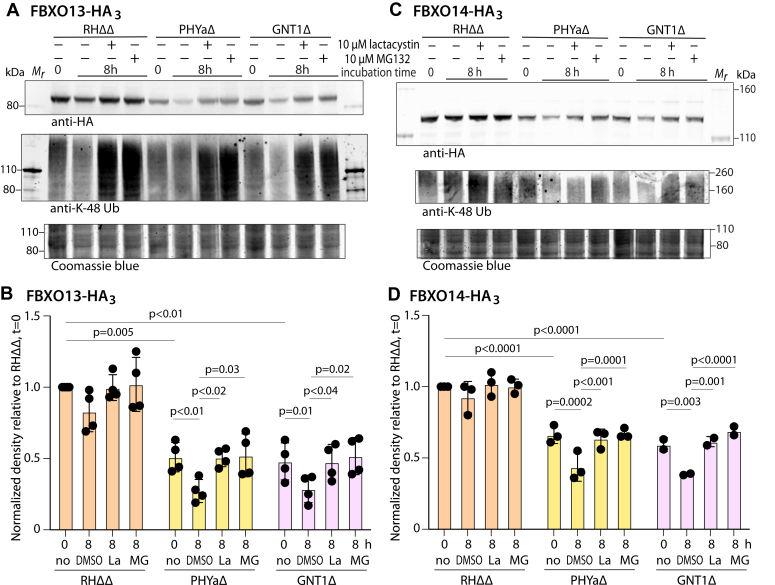
**FBXO13-HA**_**3**_**and FBXO14-HA**_**3**_**levels are increased by proteasomal inhibition.** Intracellular parasites were mechanically lysed out of hTERT fibroblasts, incubated in 10 μM MG132 (MG), 10 μM lactacystin (La), or the DMSO carrier for 8 h, and subjected to Western blot analysis using anti-HA or anti-K48-polyUb, followed by Coomassie blue staining of the blotted gel. *A*, a representative trial from FBXO13-HA_3_ expressing RHΔΔ, PHYaΔ, GNT1Δ parasites. *B*, box plots show FBXO13-HA_3_ densitometry data normalized to Coomassie blue staining from 4 independent trials. Significance was assessed using two-way ANOVA with repeated measures at α0.05. *C*, FBXO14-HA_3_ levels were analyzed as in panel A. *D*, summary from 2 or 3 independent trials.

To address whether increased degradation contributes to the reduced levels of FBXO13 and FBXO14, RHΔΔ, PHYaΔ, and GNT1Δ parasites were lysed out of host cells and subjected to treatment with either of two inhibitors of the *Toxoplasma* proteasome (25, 26), which mediates the end step in Ub-proteasome system dependent protein degradation. Parasites were treated extracellularly to avoid confounding effects of inhibiting host proteasomes. Pilot studies showed that 3 to 10 μM MG132 or lactacystin maximally promoted the accumulation of K48-linked polyubiquitin chains and FBXO13-HA_3_ in PHYaΔ cells. As shown by the example in Fig. 5*A* and quantified over 4 independent experiments in Fig. 5*B*, FBXO13-HA_3_ levels declined further in PHYaΔ and GNT1Δ parasites over the 8-h period of extracellular treatment, but not significantly for RHΔΔ. Treatment with either MG132 or lactacystin preserved the levels observed at 0 h, suggesting that the decline over the 8 h interval was due to proteasome-mediated degradation. Accumulation of poly-ubiquitinated proteins using an antibody selective for K48-linked Ub chains confirmed the activity of the inhibitors. Similar trends were observed for FBXO14-HA_3_ levels (Fig. 5, *C* and *D*). SKP1 levels were not significantly affected over the course of these experiments. The similar effects of independent inhibitors diminish the likelihood that off-target effects are involved. Given the general downregulation of protein translation in extracellular parasites (27), the findings suggest that increased turnover of FBXO13 and FBXO14, possibly *via* the SCF complex, contributes to their relative loss when SKP1 is not modified.

### Oxygen-dependent regulation of FBXO13 and FBXO14

**Figure 6:**
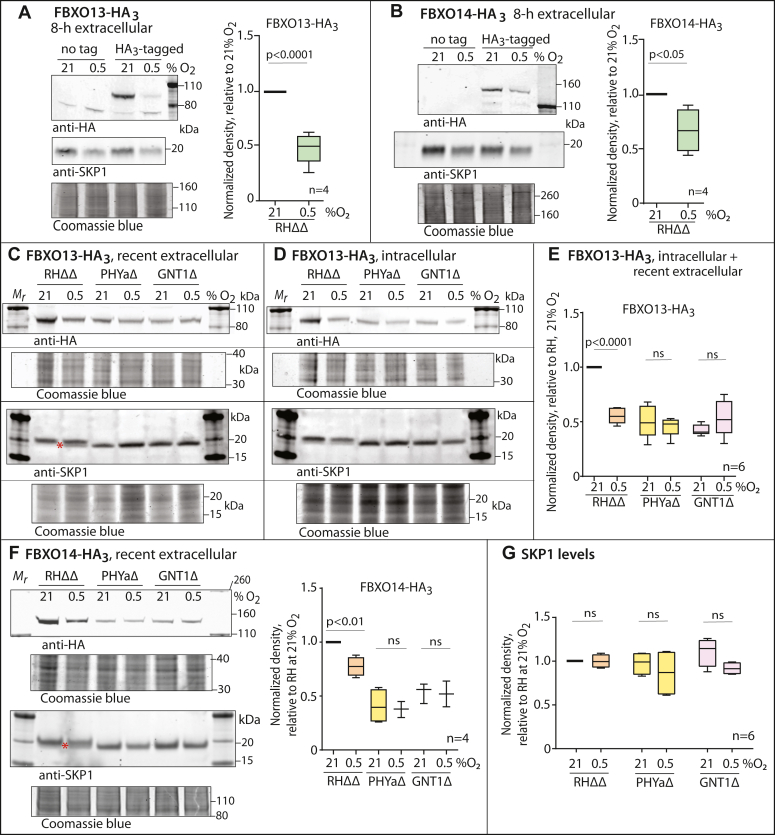
**Dependence of FBXO13-HA**_**3**_**and FBXO14-HA**_**3**_**on oxygen.** Parasites (RHΔΔ, PHYaΔ, and GNT1Δ) were incubated for 42 h at 21% or low (0.5%) O_2_ in HFFs or hTERT cells preincubated in their respective O_2_ level, and Western blotted for FBXO13-HA_3_ and SKP1. *A*, extracellular parasites that had spontaneously lysed from hTERT cells within the previous 6 to 8 h were analyzed. Levels were quantitated by densitometry ratio to Coomassie blue staining. *B*, similar for FBXO14-HA_3_. *C*, a representative example of recent spontaneously lysed extracellular parasites (collected shortly following natural emergence from high MOI infections) grown on HFFs was analyzed by Western blotting. The red asterisk denotes a more rapidly migrating and likely unmodified isoform of SKP1, also noted in panel F. *D*, a representative example of intracellular parasites (collected from low MOI infections) grown on HFFs. *E*, densitometric analysis of FBXO13-HA_3_ data pooled from all samples. *F*, a representative example of recently emerged extracellular FBXO14-HA_3_ parasites, together with a densitometric analysis of FBXO14-HA_3_ levels. *G*, SKP1 levels from all samples of intracellular and recent lysed extracellular parasites were aggregated and densitometrically analyzed relative to Coomassie blue-stained gels (post-blot). Significance was tested using an unpaired *t* test. n.s., difference not statistically significant.

The deletion of PHYa was examined initially as a surrogate for the response of the parasite to low O_2_. Therefore we next tested whether low O_2_ had similar effects on FBP levels in normal parasites. The HA_3_-tagged strains were grown in hTERTs or HFFs at high (21%) or low (0.5%) O_2_ for 48 h, and protein levels were analyzed by Western blotting. Initial studies performed on extracellular parasites recovered from hTERT cultures showed substantially reduced levels of FBXO13-HA_3_ and FBXO14-HA_3_ at low O_2_, as was observed in PHYaΔ cells and, unexpectedly, also reduced levels of SKP1 (Fig. 6, *A* and *B*). To address the origin of the effect on SKP1, which was not observed in PHYaΔ or GNT1Δ cells, subsequent studies were performed after less extracellular residence time and on HFFs. Unlike hTERT cells, HFFs are contact inhibited and might provide a more homogeneous O_2_ environment, and address possible host cell effects. Under these conditions, SKP1 levels were unaffected by low O_2_ in freshly emerged parasites whereas FBXO13-HA_3_ levels were similarly low, comparable to the level in PHYaΔ and GNT1Δ cells at 21% O_2_ (Fig. 6*C*). As expected, parasites still residing within HFFs showed the same effect (Fig. 6*D*), as quantified in Fig. 6*E*. In these trials, a modest fraction of SKP1 in the low O_2_ samples appeared to migrate more rapidly, at the position of SKP1 from PHYaΔ cells (red asterisks in panels C and F), as expected if hydroxylation and glycosylation of newly synthesized SKP1 were inhibited; this possibility is examined further below. A similar trend was observed for FBXO14-HA_3_ but the effect of low O_2_ on its level was not as pronounced as in PHYaΔ cells (Fig. 6*F*). Interestingly, the growth of PHYaΔ or GNT1Δ parasites at 0.5% O_2_ did not further reduce their FBXO13-HA_3_ and FBXO14-HA_3_ levels, suggesting that the effect of low O_2_ on their abundance was mediated by SKP1 modification. Total levels of SKP1 were not affected in freshly emerged and intracellular parasites (Fig. 6*G*), thus excluding a role for total SKP1 levels in the effect on the FBPs. Overall, these findings suggest that O_2_ regulates the levels of FBXO13 and FBXO14 *via* an effect on SKP1 modification by PHYa.

### Effects of Skp1 modification and O_2_ on gene expression of FBPs and Skp1

**Figure 7:**
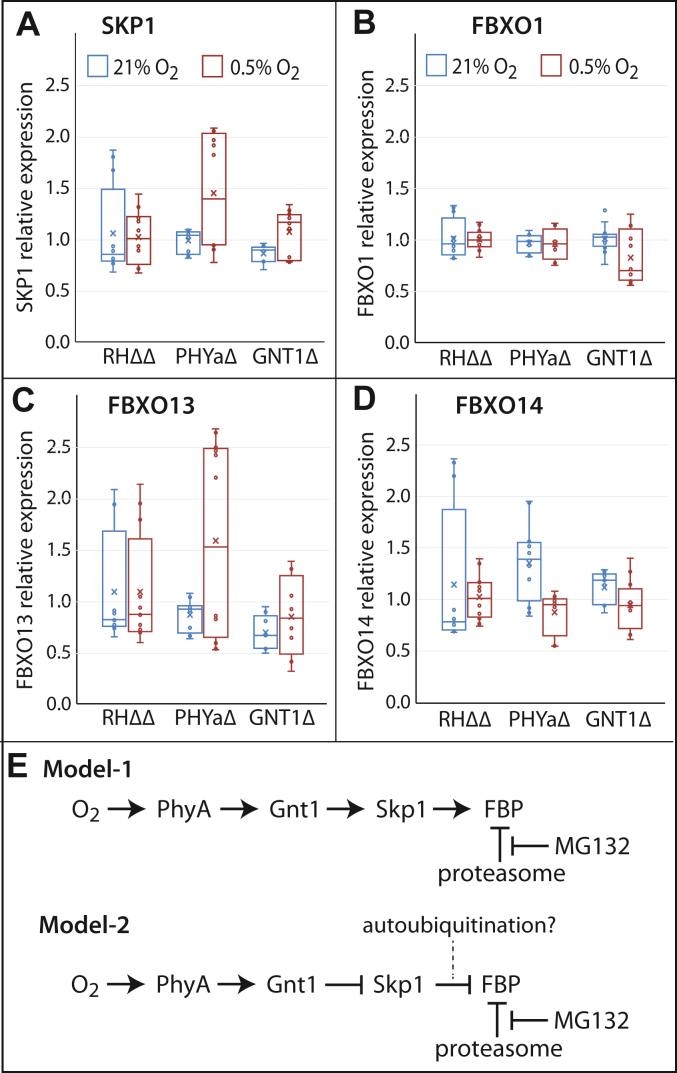
**Gene expression analysis of SKP1 and selected FBPs.***A–D*, parasites were grown on hTERT fibroblasts for 48 h at low (0.5%) or normal (21%) oxygen levels until they partially (50–75%) spontaneously lysed out. Relative expression levels for each gene were quantified *via* qRT-PCR using actin-1 and β-tubulin as housekeeping genes. Fold-change RNA expression values for SKP1 (*A*), FBXO1 (*B*), FBXO13 (*C*), and FBXO14 (*D*) were calculated relative to the housekeeping RNA values using the ΔΔCt method. Boxplots represent expression values for 4 independent biological replicates, each with 3 technical replicates, with the whiskers representing the lowest and the highest values of the dataset. Boxes represent the two middle quartiles, the line within the box represents the median, and the x marker represents the mean. Significance was tested using an unpaired *t* test. No differences within groups were statistically significant below *p* = 0.05. *E*, mechanistic models. See text.

To address the possibility that the reduced levels of FBXO13-HA_3_ and FBXO14-HA_3_ were the result of reduced transcript levels available for protein translation, their mRNA levels were quantified *via* qRT-PCR as fold-change relative to actin-1 and β-tubulin as housekeeping genes. As expected, Skp1 and FbxO1 mRNA levels were not affected in PHYaΔ and GNT1Δ parasites at 21% O_2_ (Fig. 7, *A* and *B*) and, similarly, levels of FbxO13 and FbxO14 were also not significantly decreased in PHYaΔ or GNT1Δ parasites (Fig. 7, *C* and *D*). Furthermore, incubation of parasite cultures for 48 h at low O_2_ also failed to affect mRNA levels for any of the 4 genes in either of the 3 strains. Overall, transcription of these four genes, as assessed by steady-state levels of their transcripts, is not sensitive to O_2_ levels or Skp1 modification, at least relative to the housekeeping genes actin-1 and β-tubulin. The results are consistent with the reduced levels of FBXO13-HA_3_ and FBXO14-HA_3_ in PHYaΔ and GNT1Δ relative to RHΔΔ parasites, and at 0.5% O_2_ in all strains, being mediated by protein degradation.

### Effects of low O_2_ on Skp1 modification

The mimicking of the effect of low O_2_ on FBXO13 and FBXO14 relative to the deletion of PHYa or GNT1 led us to predict that Skp1 modification would be reduced, as suggested by the appearance of a more rapidly migrating SKP1 band in SDS-PAGE gels of parasites reared at 0.5% O_2_ in our previous study (19). However, a smaller fraction of SKP1 appeared to migrate more rapidly in the present study (Fig. 6), even when samples were reduced and alkylated prior to SDS-PAGE to sharpen migration. Thus we employed quantitative mass spectrometry of SKP1 peptides to directly assess the modification status of Skp1 at high and low O_2_. For an internal standard for unmodified SKP1, a peptide corresponding to the natural tryptic peptide including Pro154 was synthesized with a heavy isotope of Val (^13^C_5_,^15^N), resulting in a mass increment of +12 (owing to the presence of 2 Val residues) relative to the native peptide from SKP1 (Fig. S11). To monitor modifications of Skp1, corresponding heavy peptides containing, in place of Pro, 4(*trans*)-hydroxyproline (Hyp) or GlcNAc α-linked to the Hyp, were synthesized. The GlcNAc-Hyp isoform is the terminal modification in PGTaΔ cells (12). Parallel HFF cultures containing PGTaΔ parasites expressing SKP1-SF were maintained at 21% or 0.5% O_2_, harvested for intracellular parasites, subjected to immunoprecipitation with mAb M2 (anti-FLAG), and introduced into our proteomics workflow after supplementation with the heavy peptide standards as internal standards.

Western blot analysis of the SKP1-SF IPs prior to proteolysis indicated that >80% of total SKP1-SF was captured by and released from the mAb M2 beads, indicating that the majority of SKP1 was sampled (Fig. 2*C*). After proteolysis, the three isoforms of SKP1 peptide_145-161_ were quantitated based on their abundance relative to the absolute amount of the corresponding synthetic heavy peptides spiked into the samples, as calculated by Proteome Discoverer 2.5. Examples of the mass spectrometry profiles are shown in Fig. S12. For SKP1-SF IPed from the PGTaΔ strain grown at 21% O_2_, only the Pro and GlcNAc-Hyp isoforms were detected, as expected (Table 3). As controls, the GGFGGn-Pro and the unmodified (Pro) isoform were detected in the RHΔΔ strain, only the Pro isoform was detected in the PHYaΔ strain, and only the Pro and Hyp isoforms were detected in the GNT1Δ strain, as previously reported (12). To assess the effect of low O_2_ on Skp1 modification, PGTaΔ parasites were grown at high or low O_2_ for 42 h. Two independent experiments with matched parasites grown at 21% or 0.5% O_2_ were performed. In both cases, low O_2_ had a detectable though modest effect on the steady-state levels of SKP1 isoforms. On the first replicate, SKP1-SF from the high O_2_ cultures contained 18% Pro-peptide_145-161_ and 82% GlcNAc-Hyp-peptide_145-161_, whereas SKP1-SF from low O_2_ cultures contained 24% Pro-peptide_145-161_ and 76% GlcNAc-Hyp-peptide_145-161_. On the second replicate, we measured 36% Pro-peptide_145-161_ and 64% GlcNAc-Hyp-peptide_145-161_ on SKP1-SF from the high O_2_ cultures contained, whereas SKP1-SF from low O_2_ cultures contained 41% Pro-peptide_145-161_ and 58% GlcNAc-Hyp-peptide_145-161_. This result was consistent with the increased presence of a more rapidly migrating band of SKP1 at low O_2_ (Fig. 6, *C* and *F*), but the modest extent of the increase of unmodified SKP1 contrasted with the complete conversion to this form in PHYaΔ cells. The results point to the significance of this unmodified subpopulation in mediating the effects of low O_2_ on FBXO13 and FBXO14.

**Table 3:** Quantitation of steady state levels of SKP1 isoforms at two O_2_ concentrations

Sample	21% O_2_		0.5% O_2_	
	Raw abundance	% Occupancy	Raw abundance	% Occupancy
expt. 1				
Pro-peptide	1.28 E+06	18.0	2.65 E+06	24.3
Heavy Pro-peptide	1.20 E+06		1.72 E+06	
Hyp-peptide	n.d.		n.d.	
Heavy Hyp-peptide	9.66 E+05		1.57 E+06	
Gn-Hyp-peptide	5.84 E+06	82.0	8.25 E+06	75.7
Heavy Gn-Hyp-peptide	1.51 E+06		2.05 E+06	
expt. 2				
Pro-peptide	6.17 E+06	35.6	3.03 E+07	41.5
Heavy Pro-peptide	4.69 E+06		1.06 E+05	
Hyp-peptide	n.d.		n.d.	
Heavy Hyp-peptide	3.86 E+06		2.36 E+05	
Gn-Hyp-peptide	1.11 E+07	64.4	4.26 E+07	58.5
Heavy Gn-Hyp-peptide	1.53 E+06		8.83 E+04	

SKP1-SF was immunoprecipitated from extracts of intracellular PGTaΔ parasites that had been grown and processed under ambient conditions (21% O_2_) or in a glove box maintained at 0.5% O_2_. Synthetic peptides corresponding to the three potential SKP1 isoforms expressed by PGTaΔ cells and containing stable isotopes (^13^C/^15^N-Val) to distinguish them from the sample peptides were introduced following endo Lys-C/trypsin digestion of the samples. Spectral counting by nLC/mass spectrometry was employed to quantitate levels of cellular unmodified and modified tryptic peptides relative to the 3 internal standards of heavy synthetic peptides. Results from two independent trials, each including 3 to 4 technical replicates, are presented separately. As expected (12), no higher glycoforms of Skp1 were detected. n.d., not detected.

### Effects on localization of FBXO13 and FBXO14

**Figure 8:**
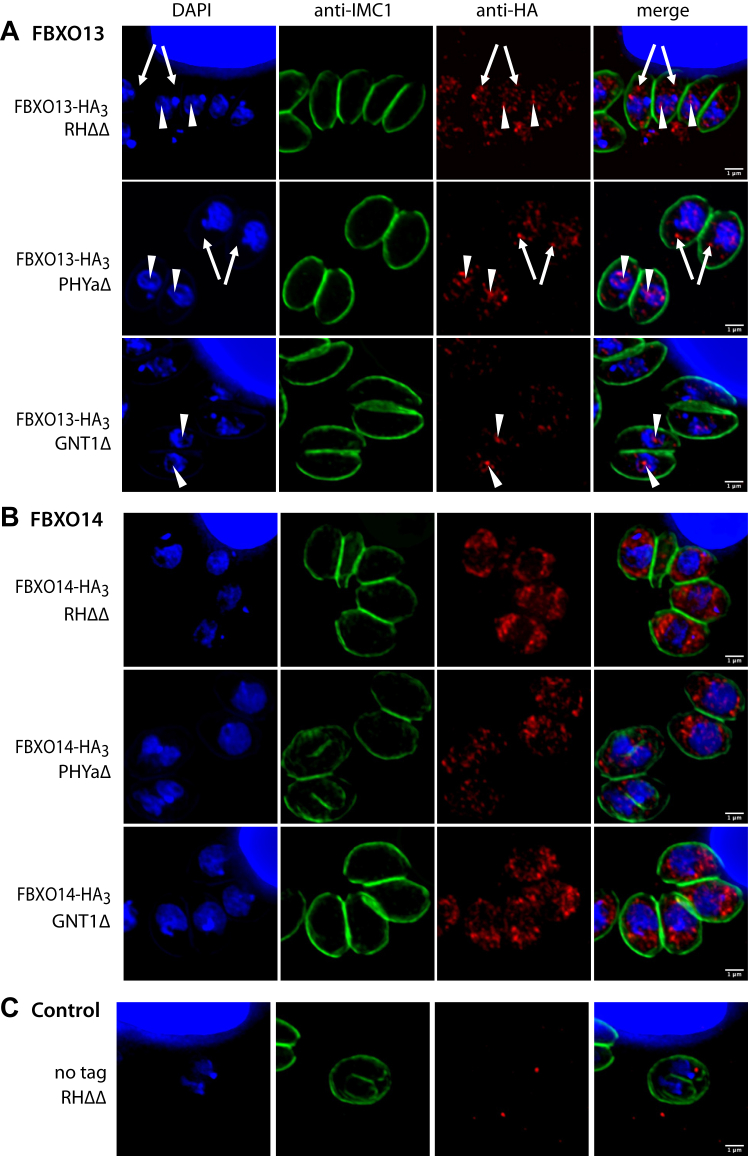
**Immunolocalization of FBXO13-HA**_**3**_**and FBXO14-HA**_**3**_**.***A* and *B*, HFFs infected with parasite strains expressing (*A*) FBXO13-HA_3_ or (*B*) FBXO14-HA_3_ in RHΔΔ, PHYaΔ or GNT1Δ genetic backgrounds were maintained at 21% O_2_ and MeOH fixed. An antibody against IMC1 (*green*) highlights the inner membrane complex at the parasite periphery, DAPI (*blue*) identifies both host and parasite nuclei, and anti-HA localizes the FBP as indicated. *Arrowheads* indicate parasite nuclei, and *arrows* indicate the cytoplasmic region. *C*, Similarly for the untagged parental RHΔΔ strain. Scale bars = 1 μm. See Fig. S10 for comparative findings at 0.5% O_2_.

To address the possibility that O_2_ exerts its effect by affecting the compartmentalization of FBXO13 and FBXO14, their localization was examined by immunofluorescence. FBXO13-HA_3_ was detected using rabbit anti-HA IgG and found to partially colocalize with the DAPI-stained nuclei of parasites residing within parasitophorous vacuoles of HFFs maintained at 21% O_2_ (Fig. 8*A*, arrowheads). Fluorescence was also abundant in extranuclear regions indicating presence in the cytoplasm (arrows), and negative in the untagged RHΔΔ strain (Fig. 8*C*). Nuclear accumulation is consistent with the presence of two potential importin-α dependent monopartite nuclear localization signals (Fig. S3), and was expected based on its homology with mammalian JMJD6 which hydroxylates nuclear-splicing factors and chromatin proteins. The intensity of labeling was reduced in PHYaΔ and GNT1Δ parasites, and in RHΔΔ and parasites maintained at 0.5% O_2_ (Fig. S10*A*), but in neither case did the pattern of labeling appear to be affected, at least within the resolution of the method.

FBXO14-HA_3_ appeared to be exclusively extranuclear in intracellular parasites at 21% O_2_ (Fig. 8*B*). This is consistent with the lack of a predicted nuclear localization signal as well as studies on the spatial proteome map of *T*. *gondii* tachyzoite (28) which indicate FBXO14 to be cytosolic. The occasional instance of fluorescence overlaying the nucleus was due to cytoplasm either above or below the nucleus as determined by inspection of each of the 16 layers of the z-stack that was summed in these maximum projection images. As for FBXO13-HA_3_, the pattern of FBXO14-HA_3_ labeling was not noticeably altered in PHYaΔ and GNT1Δ parasites, or in RHΔΔ and mutant parasites maintained at 0.5% O_2_ (Fig. S10*B*).

## Discussion

Here we identify and characterize a clear proteomic phenotype of *Toxoplasma* tachyzoites grown under low (0.5%) O_2_ compared to ambient (21%) levels. The effect is manifested as >2-fold reduced levels of FBXO13, a probable nuclear lysyl hydroxylase expected to be involved in epigenetic modifications (21, 22), and FBXO14, a cytoplasmic protein of unknown function (Figs. 3*B* and 8*B*). These two proteins share a common feature: a stable interaction with SKP1, a subunit of the E3(SCF) class of polyubiquitin ligases involved in regulated protein degradation. SKP1 is modified by the O_2_-dependent prolyl hydroxylase PHYa, whose *in vitro K*_m_ for O_2_ is ∼1 μM (29, 30), making it a candidate for mediating the effect of low O_2_. Genetic deletion of PHYa resulted in even lower levels of FBXO13 and FBXO14 (Fig. 4), consistent with its being the responsible O_2_ sensor. Genetic deletion of GNT1, which acts on the SKP1-OH product and whose specificity for modifying SKP1 is more confidently established than is the specificity of PHYa (12), confirmed that deleting PHYa is mediated *via* SKP1. Finally, the effect of low O_2_ was not significantly amplified in PHYaΔ or GNT1Δ strains (Fig. 6), reinforcing the role of PHYa in mediating the low O_2_ response.

FBP/SKP1 complexes are conditionally associated with Cul1 to comprise a full E3 Ub ligase that has the potential to assemble K48-linked Ub chains on proteins bound by substrate receptor FBPs. Proteins tagged with these Ub chains are recognized by factors that associate them with the 26S-proteasome for degradation. The finding that interfering with proteasome activity using either of two inhibitors protects FBXO13 and FBXO14 from the time-dependent breakdown in PHYaΔ and GNT1Δ parasites (Fig. 5), coupled with the finding that there is no evidence for a change in transcript levels (Fig. 7), indicates that their reduced levels resulted from increased degradation *via* the ubiquitin-proteasome system. Given the precedent that FBPs themselves can be SCF substrates (31), increased degradation of FBXO13 and FBXO14 might be mediated by the SCF itself. Previous studies have indicated that full glycosylation influences the ensemble of conformations visited by the C-terminal intrinsically disordered region of SKP1 from *Dictyostelium* (13, 16), and evidence supports a similar effect in *Toxoplasma* SKP1 (15). These effects were interpreted as promoting interaction with F-box domains. Other evidence suggests that glycosylation of *Dictyostelium* SKP1 inhibits its homodimerization (17), which competes with FBP binding (18). This has also been reasoned to promote the accessibility of SKP1 to interact with FBPs. The increased potential interaction of glycosylated Skp1 with FBPs suggested by these findings would imply greater ubiquitination activity, which is inconsistent with decreased degradation observed for FBXO13 and FBXO14. Thus their degradation might occur *via* a different mechanism, or the role of glycosylation might in some way protect these FBPs. The wide range of F-box sequences (Fig. S2) that interact with the single SKP1 protein certainly allows for varied effects on different proteins.

Both *Toxoplasma* and *Dictyostelium* possess two homologs of FBXO13. *Toxoplasma* FBXO13 and *Dictyostelium* JcdH each possess F-box domains and are considered orthologs (Fig. 4*A*). The *Dictyostelium* paralog, JcdI, also possesses an F-box but is divergent from the *Toxoplasma* paralog, JMJD6A, which lacks an F-box sequence and resides in a clade that includes human JMJD6 (10). The lysyl hydroxylase activity of human JMJD6 is predicted to occur in all of these paralogs but remains to be confirmed though dioxygenase activity was demonstrated for *Dictyostelium* JcdI (10). Comparative studies on *Dictyostelium* JcdI also show it to be enriched in the interactome of glycosylated Skp1 of proliferating cells relative to a glycosylation mutant, similar to findings for *Toxoplasma* FBXO13. In contrast, this is not associated with a difference in JcdI abundance. However, at the slug stage of development, the abundance of JcdI is decreased when Skp1 is glycosylated, opposite to the effect observed in proliferating *Toxoplasma*. Though it is unknown whether the effect on JcdI abundance is direct or indirect, it is evident that additional factors contribute to developmentally regulating its degradation. It is likely that the combination of F-box sequence differences as well as additional factors in addition to Skp1 modification have evolved to tune the abundance of this enzyme, which is critical for *Toxoplasma* viability. As for *Dictyostelium* JcdI (10), we propose that FBXO13 is not a canonical FBP substrate receptor, but rather an enzyme whose abundance is regulated *via* its stable association with Skp1 that conditionally associates with Cul1 to lead to its degradation. Two potential mechanisms are presented in Fig. 7*E*. Model-1 describes a mechanism by which SKP1 modification protects select FBPs from degradation, and Model-2 describes a mechanism by which glycosylation inhibits FBP autoubiquitination. Possibly an association of FBXO13 with unmodified SKP1, as would occur at low O_2_, leads to its polyubiquitination and degradation together with its associated SKP1 in the 26S-proteasome, resulting in limited accumulation of unmodified SKP1. Further studies are needed to explore these mechanisms.

FBXO14 is, in contrast to FBXO13, found only in the coccidian clade of apicomplexans (Fig. 1*C*), which is where SKP1 glycosylation is confined as if they co-evolved. Its potential role as a substrate receptor is unknown. It is an abundant FBP that is stably associated with SKP1, and its abundance is regulated in parallel with FBXO13. However, there is no obvious similarity between their F-box sequences (Fig. S2).

FBXL2, FBXO5, FBXO6, FBXO11, FBXO12, and possibly FBXO4 were also more highly represented in the interactome of glycosylated SKP1-SF (Table 2). However, the effect is selective because it was not observed for another major characterized FBP, FBXO1 (Fig. 4*C*), and other FBPs (Table 2). Preferential representation was observed also for proteins for which an F-box domain could not be predicted (Table 1). Their presence in the SKP1 interactome and the dependence of some on PHYa, coupled with the difficulty of accurately predicting F-box domains, allows for the possibility that they are cryptic FBPs or co-factors for FBPs. The occurrence of substrate-receptor-like WD-40 repeat domains in some interactors is an additional feature suggestive of a potential FBP. Whether or not their increased representation in the interactome of SKP1 when it is glycosylated (as in RHΔΔ parasites at 21% O_2_) is due to higher abundance as for FBXO13 and FBXO14 remains to be examined.

Regulation of the interactome of SKP1 by PHYa and O_2_ is an evolutionarily conserved mechanism shared by numerous protist groups (32). In *Dictyostelium,* where it was first discovered, glycosylation similarly increases the representation of a fraction of *Dictyostelium* FBPs in the SKP1 interactome. In further concordance between these two unrelated species, there were no instances of decreased representation and the representation of many FBPs was not affected (10). Detailed studies of JcdI (cited above) and FbxwD showed that increased representation was not necessarily associated with a change in abundance (10). Thus the effect of increased representation may vary according to the protein and, as indicated for JcdI in *Dictyostelium*, other conditional factors.

These findings are likely relevant to the phenotype of *Toxoplasma* type II PHYaΔ parasites when introduced into mice *via* oral gavage (33), which mimics the route of natural infection and exposes the parasite to the natural range of O_2_ levels as it comes to its final location and differentiates into bradyzoite cysts in brain or muscle. Though TgPHYa mutant parasites can establish an infection in the gut, they do not efficiently disseminate to peripheral tissues because of their reduced ability to survive within recruited immune cells. This inability is lost in IFNγ knockout mice, and evidence suggests that PHYa is required to scavenge tryptophan, an amino acid that IFNγ decreases by inducing the tryptophan catabolizing enzyme, indoleamine dioxygenase. FBPs that are reduced in level in PHYaΔ cells are candidates for mediating resistance to IFNγ in normal cells. These provide new leads for further studies to pinpoint the specific candidate FBP that is responsible. As a likely enzyme, FBXO13 has the potential to modulate gene expression as suggested by the role of its human paralog.

## Experimental procedures

### Cell culture

Parasite strains (Table 4) were passaged on hTERT fibroblasts or HFFs in 1% (v/v) fetal bovine serum in DMEM at 37˚C under 5% CO_2_ as previously described (15). Host cells were maintained at 10% serum until infection. Tachyzoites were analyzed after substantial spontaneous egress (EC, extracellular) or mechanically lysed out (IC, intracellular). Alternatively, tachyzoites were isolated from cultures where spontaneous lysis was incomplete, which is described by % EC as indicated. For low-O_2_ (0.5%) incubations, parasites were incubated inside a humid chamber within a polycarbonate O_2_ Control InVitro Glove Box (Coy Laboratory Products, Inc.) for 42 h, with the balance composed of N_2_. Host cells were pre-incubated for 18 h at low O_2_ prior to infection. Parasites were observed, syringe-lysed, and recovered by centrifugation in the glove box. Parasites were used for transfection or immediately frozen at −80 °C for later analysis, as indicated.

**Table 4:** Strains employed in this study

Description	Name	Parental	Genotype	Selection	Reference
		strain		marker	
RH (type 1)					(42)
RHΔΔ	RH		Δ*ku80*/Δ*hxgprt*		(43)
PHYaΔ	KR01	RHΔΔ	Δ*phy*a(exon-1)/Δ*ku80*	Hxgprt	(29)
GNT1Δ	KR04	RHΔΔ	Δ*gnt*1/Δ*ku80*	Hxgprt	(12)
PGTAΔ	KR08	RHΔΔ	Δ*pgt*a/Δ*ku80*	Hxgprt	(12)
SKP1-SF	KR16	RHΔΔ	*skp1-SF*^*Hxgprt*^/Δ*ku80*	MPA/xanthine	(12)
SKP1-SF/PHYaΔ	KR80	PHYaΔ-2-KR01	*skp1-SF*/Δ*phy*a/Δ*ku80*	CAT	(12)
SKP1-SF/GNT1Δ	MM49	GNT1Δ-KR04	*skp1-SF*/Δ*gnt*1/Δ*ku80*	CAT	This report
SKP1-SF/PGTaΔ	MM52	PGTaΔ-KR08	*skp1-SF*/Δ*pgt*a/Δ*ku80*	CAT	This report
FBXO1-HA_3_, cl. H6	BD3	RHΔΔ	*fbxo1-HA*_*3*_/Δ*ku80*/Δ*hxgprt*	DHFR	This report
FBXO1-HA_3_/PHYaΔ	BD4	PHYaΔ-KR01	*fbxo1-HA*_*3*_/Δ*phy*a/Δ*ku80*	DHFR, Hxgprt	This report
FBXO13-HA_3_	MM31	RHΔΔ	*fbxo13-HA*_*3*_/Δ*ku80*/Δ*hxgprt*	CAT	This report
FBXO13-HA_3_/PHYaΔ	MM40	PHYaΔ-KR01	*fbxo13-HA*_*3*_/Δ*phy*a/Δ*ku80*	CAT,Hxgprt	This report
FBXO13-HA_3_/GNT1Δ	MM44	GNT1Δ-KR04	*fbxo13-HA*_*3*_/Δ*gnt*1/Δ*ku80*	CAT, Hxgprt	This report
FBXO14-HA_3_	MM34	RHΔΔ	*fbxo14-HA*_*3*_/Δ*ku80*/Δ*hxgprt*	CAT	This report
FBXO14-HA_3_/PHYaΔ	MM38	PHYaΔ-KR01	*fbxo14-HA*_*3*_/Δ*phy*a/Δ*ku80*	CAT,Hxgprt	This report
FBXO14-HA_3_/GNT1Δ	MM48	GNT1Δ-KR04	*fbxo14-HA*_*3*_/Δ*gnt*1/Δ*ku80*	CAT, Hxgprt	This report

All strains were derived from type I strain RHΔhxgprtΔku80, which is abbreviated as RHΔΔ and is, for convenience, referred to as the normal strain whether or not it has been edited for epitope tagging of the encoded protein.

### Proteasome inhibition studies

Tachyzoites were mechanically lysed out of host cells (ICs) and washed 1 × with DMEM media by centrifugation at 2000*g* for 8 min. Parasites were resuspended in 5 ml of complete media with 10 μM MG132 diluted from a 30 mM stock solution in dry DMSO or 10 μM lactacystin from a 3 mM stock solution in dry DMSO. Media for each strain and treatment were supplemented to the same concentration of DMSO, which was 0.0032% (v/v) DMSO for the highest inhibitor concentration tested (10 μM), and cultured in 6-well plates for 8 h. Parasites were collected by centrifugation and stored at −80 °C for further analysis by Western blotting.

### Generation of SKP1-SF tagged parasites in PHYaΔ, GNT1Δ, and PGTAΔ parasites

The C-terminus of SKP1 was modified with an SF-epitope tag in strains PHYaΔ-2, GNT1Δ, and PGTaΔ by insertion from the pSF-TAP-LIC-CAT plasmid by conventional homologous recombination as previously described (12). SF tagged parasites were selected with 20 μM chloramphenicol and SKP1-SF clones were verified by western blotting (Fig. 2*C*).

### HA_3_-epitope tagging of FBXO13 and FBXO14

A single guide (sg) RNA CRISPR/Cas9 plasmid targeting just downstream of the stop codon of JMJD6b (TGGT1_283890) (Fig. S3), was prepared as previously described (34). An optimal gRNA was selected based on an efficiency score using the CRISPR gRNA design tool EuPaGDT (grna.ctegd.uga.edu). A DNA transfection amplicon was generated by PCR of the HA_3_-coding DNA sequence with chloramphenicol drug resistance marker from plasmid p3xHA.LIC.CAT (35) with primers consisting of 45 bp flanking the CRISPR disruption site (Table S1). The gene editing strategy is outlined in Fig. S4*A*.

Freshly lysed parasites were co-transfected with the purified sg CRISPR plasmid (12.5 μg) and the DNA amplicon (1.5 μg), as described (34). Stable parasite clones were selected after propagation for 3 passages in the presence of chloramphenicol (20 μM) and then cloned by limiting dilution. Specific integration of the HA_3_-tag coding DNA into the FBXO13 locus was confirmed by PCR (Fig. S4*B*), and expression of FBXO13-HA_3_ was detected by Western blotting with anti-HA mAb 12CA5 (Fig. 3*A*).

For PCR screening, frozen cell pellets were processed for PCR using a Monarch Genomic DNA Purification Kit from New England Biolabs (NEB) for recovering gDNA. A gene-specific and HA tag-specific primer pair was chosen using a web-based oligo analysis tool (Multiple Primer Analyzer, ThermoFisher). The most efficient annealing temperatures were determined using the NEB *T*_*m*_ Calculator (tmcalculator.neb.com), and Q5 DNA polymerase (NEB) was typically used for amplification. A typical thermocycling reaction consisted of 30 to 35 cycles of denaturation at 98 °C for 10 s, annealing at primer pair-specific temperature for 30 s, and extension at 72 °C for 30 s/1000 base pairs, in an Eppendorf Mastercycler Nexus instrument. PCR products were examined on 1% agarose gels containing 0.5 μg/ml ethidium bromide and imaged under ultraviolet illumination (302 nm). The NEB 2-log DNA ladder was used as the size standard.

Sequencing was used to further confirm the identity of the gRNA plasmids and tagged strains. Either the gRNA plasmids or diagnostic PCR products were used as the templates for the sequencing reactions, respectively. The gRNA plasmids were sequenced with a gRNA-specific primer and a vector-specific primer on the top and bottom strands, respectively. The diagnostic PCR products from the tagged strains were sequenced in both directions with primers that were designed as described above. Sequencing reactions were performed by Eton Bioscience, Inc.

FBXO14 was similarly HA_3_-tagged using the guide RNA described in Fig. S6, as outlined in Fig. S7*A*. Accurate insertion was confirmed by PCR (Fig. S7*B*) and Western blotting (Fig. 3*B*).

### HA_3_-epitope tagging of FBXO1

A C-terminally HA_3_-tagged FbxO1 strain, which was previously generated by conventional homologous recombination (19), was rederived using the CRISPR/Cas9 method described above (Fig. S9*A*). The gRNA sequence (Table S1) was cloned into the pU6-Universal plasmid vector, which also expresses Cas9. The tagging DNA included dihydrofolate reductase, and genomic incorporation was selected for using 1.0 μM pyrimethamine (Sigma). Clones were analyzed by diagnostic PCR (Fig. S9*B*), Western blotting (Fig. S9*C*), and verified by sequencing of the genomic PCR products (Fig. S9*D*). Incorporation was observed to be error-prone, and clones A11 and H6 were chosen for further studies.

### SDS-PAGE and Western blotting

Cell pellets were routinely resuspended in 1% SDS, 10 mM Tris-HCl (pH 8.0), 1 mM EDTA at a minimal cell concentration of 5 × 10^5^/μl, boiled for 5 min, and centrifuged for 15 min at 21,000*g*. The supernatant was mixed with 0.25 vol of 4 × SDS Sample Buffer with DTT and boiled for 5 min. In some trials, SKP1 extracts prepared at 1 × 10^6^ cells/μl were reduced and alkylated. Samples were diluted 4-fold with 0.90 M Tris-HCl (pH 8.45), 24% (v/v) glycerol, 8% (w/v) SDS, 0.01% phenol red, 0.01% Coomassie G, 10 mM DTT, and incubated at 65˚C for 30 min. Iodoacetamide was added to a final concentration of 25 mM and incubated at room temperature in the dark for 1 h. Extracts from equivalent cell numbers (5 × 10^6^) were loaded onto pre-cast NuPage 4 to 12% polyacrylamide Bis-Tris protein SDS-PAGE gels alongside Novex Sharp (Invitrogen) pre-stained protein standards. Gels were electrophoresed in 1 × MOPS buffer (50 mM Tris, 50 mM MOPS, pH 7.7, 1 mM EDTA, 3.5 mM SDS) or MES buffer (50 mM Tris, 50 mM MES, pH 7.3, 1 mM EDTA, 3.5 mM SDS) at 200V and transferred to a nitrocellulose membrane at 10 V for 7 min using an iBlot 2 electrotransfer apparatus (Thermo Fisher Scientific). SKP1 migrates as a closely spaced multiplet in the MOPS buffer but more uniformly in the MES buffer. Membranes were blocked in 5% (w/v) non-fat dried milk in 50 mM Tris-HCl (pH 7.4), 100 mM NaCl, 0.02% (w/v) NaN_3_ (TBS) for 1 h, and incubated overnight in 1:1000 affinity purified anti-Skp1 pAb UOK75 (29), or 1:1000 anti-HA mAb 12CA5, at 22 °C diluted in 5% milk in TBS. Membranes were washed in TBS 3 times and incubated in 1:10,000 Alexa Fluor-680 goat-anti-rabbit for pAb UOK75, or 1:10,000 Alexa Fluor-680 goat-anti-mouse for mAb 12CA5 diluted in 5% milk in TBS, for 1 h. Membranes were washed in TBS 3 times scanned with a Li-Cor Odyssey CLx and imaged with Li-Cor Image Studio. Either a simultaneously prepared duplicate gel or the blotted gel was stained in Coomassie Blue R250, destained, rinsed in H_2_O, and scanned at 700 nm.

Densitometry analyses were performed using Photoshop software. Images were converted to grayscale and 8 bits/channel in Adobe Photoshop. The signal level was automatically adjusted to Photoshop's default settings. A band of interest was outlined with a minimal box and the density was recorded from the image analysis tool. The same box was moved to a region lacking a visible protein signal that was then subtracted from the signal of interest. This method was systematically applied to all bands of interest within the same gel. The same measuring procedure was used for Coomassie blue stained gel except a horizontal region on the gel that was consistent across all lanes was outlined for each lane and used as the normalization factor. Data were analyzed in Microsoft Excel to calculate standard deviations, or GraphPad Prism to analyze significance using 2-way ANOVA with α0.05.

### TgSKP1interactome studies (co-IP experiments)

Protein extracts were generated from frozen tachyzoite pellets (2–6 × 10^8^) from RHΔΔ, PHYaΔ, GNT1Δ, PGTaΔ parasites expressing SF-tagged SKP1 by lysis in IP buffer (50 mM HEPES-NaOH, pH 7.4, 0.5% (v/v) Nonidet P-40, 100 mM NaCl, plus protease and phosphatase inhibitors (10 μg/ml aprotinin, 10 μg/ml leupeptin, 1 μM PMSF, 1 mM NaF, 0.2 mM NaVO_3_), on ice. The lysates were centrifuged at 21,000*g* for 20 min at 4 °C and 100 μl of the supernatant (1.2 × 10^8^ cells) were incubated with 10 μl of anti-FLAG M2 magnetic beads (Sigma, M8823) for 1 h at 4 °C, gently rocking. For reciprocal co-IP experiments with TgSKP1 interactors, similar lysates from strains expressing HA-tagged versions of FBXO1, FBXO13 or FBXO14 derived from RHΔΔ were prepared as above and 100 μl of the clear supernatants (1.2 × 10^8^ cells) were incubated with 10, 5 or 12 μl anti-HA beads (mouse anti-HA mAb clone 12CA5 bound and crosslinked to protein A/G magnetic agarose beads (Pierce, 78,610)) respectively. Cross-linking was accomplished by washing the bound beads in 0.2 M sodium borate (pH 9.0), resuspending and rotating in 20 mM DMP (dimethyl pimelimidate, Thermo Scientific # 21667) in the same buffer for 40 min at room temperature, recapturing with a magnet, resuspending in 0.2 M ethanolamine (pH 8.0) to quench the reaction, and recapturing and resuspending in phosphate-buffered saline (PBS) lacking divalent cations. Beads were captured, the unbound fraction removed, and the beads washed three times with the corresponding IP buffer, three times with 10 mM Tris-HCl (pH 7.4) containing 50 mM NaCl and once with 50 mM NaCl. Bound proteins were eluted twice with 60 μl 133 mM triethylamine (TEA, Pierce, 25,108) by incubating for 15 min at 22 °C, and immediately neutralized with 40 μl of 0.2 M acetic acid. The eluted material was dried under a vacuum. Samples were then reduced, alkylated, and converted to peptides with a mixture of Endoproteinase Lys-C and trypsin; peptides were then captured on C_18_ Zip-tips and released for MS analysis, all essentially as described (10).

### Mass spectrometry of co-IP peptides

Peptides were separated on a C18 nano-column (Thermo Acclaim PepMap 100 C18 series) using an Ultimate 3000 nano-HPLC, and directly infused into a Q-Exactive Plus Orbitrap Mass Spectrometer (Thermo Fisher), as previously described (10). Raw files were then processed in Proteome Discoverer 2.5 using a *T. gondii* GT1 protein database containing 8450 unique proteins (UniProt Proteome ID UP000005641), modified to include the revision for 6 FBPs whole gene model has changed in ToxoDB release 67 (FBXO9, FBXO11, FBXO12, FBXO14, FBXW2, and FBXW3) and 179 common ectopic contaminants (36) essentially as described (10). Proteome Discoverer 2.5 calculated protein abundances for proteins of high (FDR<0.01) and medium confidence (FDR<0.05), and identified with 2 or more peptides, were compared between controls (parental, non-tagged strains) and samples (tagged-version strains, see above) in MetaboAnalyst 6.0 data analysis tool (37). Proteins enriched >5-fold with a p-val <0.05 in SKP1-SF parasites vs. the untagged parental strain, were considered significant interactors of TgSKP1. For FBXO1, FBXO13, and FBXO14 reciprocal co-IPs, proteins enriched >5-fold with a p-val <0.05 in HA-tagged parasites vs. the untagged parental strain were considered significant interactors of the corresponding FBP. The MS proteomics data (listed in Tables S4 and S5 are deposited in the ProteomeXchange Consortium *via* the PRIDE (38) partner repository with the dataset identifiers PXD050988, and PXD050091 (Table S4, SF-coIP experiments), and PXD050011, PXD049975, and PXD049694 (Table S5, HA-coIP experiments).

### Quantification of SKP1-SF modification isoforms

Heavy versions of Skp1-prolyl peptide (H-Ile-Phe-Asn-Ile-Val(^13^C_5_,^15^N)-Asn-Asp-Phe-Thr-Pro-Glu-Glu-Glu-Ala-Gln-Val(^13^C_5_,^15^N)-Arg-OH), with the 2 Val residues containing the heavy isotopes ^13^C and ^15^N, were synthesized to match the unmodified, hydroxylated and GlcNAcylated peptides found in PHYaΔ, GNT1Δ, and PGTaΔ strains, respectively, generated by trypsin. Pro-Skp1, Hyp-Skp1, and GlcNAc-Skp1peptides (5.6–6.2 mg) were synthesized with a free N-terminus and a free carboxyl terminus by release and deprotection after sequential addition of FMOC-amino acids to Arg-resin. Isotopically enriched (^13^C_5_,^15^N)Val residues (CAS Number [1,217,442–94–8], Cambridge Isotope Laboratories, Tewksbury, MA) were incorporated resulting in an overall mass increase of +12. Purity was assessed by HPLC analysis using a ReproSil-Pur 120 C18-AQ, 5 μm column (250 × 4.6 mm), at a flow rate of 1 ml/min, using a 48-min gradient of 5 to 60% (v/v) of MeCN in 0.1% trifluoroacetic acid in water and absorbance detection at 214 nm (Fig. S11). Identity was assessed by MALDI-TOF mass spectrometry using a Shimadzu Biotech Axima in positive ion reflectron mode (Fig. S11). Additional versions were synthesized using 2*S*,4*R*-hydroxyproline (Hyp) or d-GlcNAcα-Hyp in place of the Pro residue. Synthetic peptides were reconstituted in 5% ACN, 0.05% TFA (at 4 pmol/μl), and 0.4 to 1.0 pmol of each was spiked into each immunoprecipitation sample after endo Lys-C/trypsin digestion and before C_18_ zip-tip peptide clean-up. Spiked in synthetic peptides amounts were adjusted empirically to approximately match the abundance of the corresponding endogenous/native peptides (Fig. S12). Proteome Discoverer 2.5 was used to calculate peptide abundances for SKP1-Pro peptide(s) allowing for the following modifications: hydroxylation (+15.995 Da) on Pro, hydroxylation + GlcNAcylation (+219.074 Da) on Pro, hydroxylation + (Hex3+HexNAc + dHex) addition (+851.296 Da) on Pro and heavy Val (+6.0104 Da). Total SKP1 protein abundance was calculated by adding all abundances for each of the identified SKP1. To compensate for the differential MS detection response to each of the modified SKP1-Pro peptides, peptide abundance was calculated in reference to the amount of the corresponding synthetic peptide included as an internal standard. Data for peptides with no missed cleavages are reported, but similar results when including peptides with up to 2 missed cleavages.

The mass spectrometry proteomics data have been deposited to the ProteomeXchange Consortium *via* the PRIDE (38) partner repository with the dataset identifier PXD053703 (see Table S4 for details).

### Gene expression based on real-time PCR

Total RNA was extracted from frozen tachyzoite pellets (1–2 × 10^8^) from RHΔΔ (parental), PHYAΔ , and GNT1Δ strains incubated at normal oxygen (21% O_2_) or low oxygen (0.5% O_2_) conditions with TRIzol Reagent. Briefly, extracellular tachyzoites from cultures when parasites were 50 to 75% spontaneously lysed from HFFs or hTERTs were resuspended in 1 ml TRIzol Reagent (cat. # 15596026, Life Technologies, CA) by vigorous vortexing followed by 5 min incubation at room temperature. Then, 200 μl CHCl_3_ was added, vortexed, and incubated for 10 min at room temperature. Homogenates were centrifuged at 12,000*g* for 15 min at 4 °C and ∼600 μl of clear upper phase were transferred to a new tube. RNA was then precipitated by adding 500 μl isopropanol, mixing by inversion, and incubation for 10 min at room temperature. Samples were centrifuged at 12,000*g* for 10 min at 4 °C. Supernatants were discarded and pellets were washed in 1 ml 75% ethanol. Samples were centrifuged at 12,000*g* for 5 min at 4 °C. Supernatants were discarded. Pellets were air-dried and subsequently resuspended in 30 to 50 μl RNAse-free water. Resuspended RNAs were incubated for 10 min at 55 °C and stored at −80 °C. cDNAs were synthesized from 1 μg total RNA using a MAXIMA First Strand cDNA synthesis kit with DNAse (cat. #K1671, Life Technologies). qPCR reactions were performed in 20 μl using 2 μl of a 1:10 cDNA dilution as template, 1 μl of 10 μM primers and 10 μl of Power UP SYBR master mix (cat. # A25741, Life Technologies, CA) in 96-well plates in a StepOnePlus Real-Time PCR system (Life Technologies) using the following program: 2 min at 50 °C, 2 min at 95 °C, 40 cycles at 95 °C for 3 s and 60 °C for 30 s, and imaging. Relative gene expression values for SKP1, FBXO1, FBXO13, and FBXO14 were calculated with the ΔΔCt method (39), using the average of Ct values for ACT1 (TGGT1_209030) and B-TUB (TGGT1_266960) as housekeeping genes. Measurements were done in triplicate for 4 independent biological replicates. Primer sequences are provided in Table S1.

### Immunofluorescence studies

Intracellular parasites in HFFs were fixed with ice-cold MeOH and blocked in 5% w/v bovine serum albumin (BSA) in PBS for 60 min. Primary Abs were incubated overnight at 4 °C: affinity-purified anti-TgSKP1 pAb UOK75 (19) (1:250, IMC1 antibody (gift of Gary Ward, Univ. of Vermont) (1:4000, rabbit anti-HA mAb (Cell Signaling, C29F4) (1:1000). After rinsing in PBS, samples were incubated with secondary antibodies for 60 min: Alexa Fluor 488- or Alexa Fluor 594-conjugated antibodies (1∶2000, Thermo Fisher Scientific). Images were collected using Structured Illumination Microscopy (SIM) and maximum projections are shown.

### Evolutionary studies

Searches for homologs to *T. gondii* FBPs were carried out using BLASTp in https://toxodb.org/toxo/app and Genbank, and protein data from Apicomplexa, Chromericeae and Vitrellaceae in VeuPathDB, release 64, 12 July 2023. Proteins with apparent incomplete sequences based on alignment and insertions or deletions in conserved domains were amended based on inferences from tBLASTn (default settings) searches. E-values for the full-length protein models were recorded. F-box-like sequences were searched based on sequence alignments and suggestions from NCBI CDD and SMART database searches.

## Data availability

The MS proteomics data, which are indexed in Tables S4 and S5, are deposited in the ProteomeXchange Consortium *via* the PRIDE (38) partner repository with the dataset identifiers PXD050988, PXD050091, PXD053703, PXD050011, PXD049975, and PXD049694.

## Supporting information

This article contains supporting information, with 5 tables and 12 figures. References (6, 10, 19, 40, 41) are cited therein.

## Conflict of interest

The authors declare that they have no conflicts of interest with the contents of this article.
