# Supplementary material for: Oxygen-dependent regulation of F-box proteins in Toxoplasma gondii is mediated by Skp1 glycosylation[image]
Source: J Biol Chem. 2024 Sep 21;300(11):107801. doi: 10.1016/j.jbc.2024.107801 (PMC11570480; doi:10.1016/j.jbc.2024.107801)

SUPPORTING INFORMATION

Oxygen-dependent regulation of F-box proteins in *Toxoplasma gondii* is mediated by Skp1 glycosylation

Msano N. Mandalasi[1,*], Elisabet Gas-Pascual[1,*], Carlos Gustavo Baptista[2], Bowen Deng[1,3], Hanke van der Wel[1], John A.W. Kruijtzer[4], Geert-Jan Boons[4,5], Ira J. Blader[2], and Christopher M. West[1,6]

Table of Contents

Table S1. Oligonucleotides used in this study
Table S2. Gene Identifiers
Table S3. Evolutionary analysis of *Toxoplasma* FBPs. Numerical data supporting Fig. 1C
Table S4. Index of Proteome Discoverer 2.5 search results of SKP1-SF co-immunoprecipitation experiments and ProteomeXchange Consortium accession numbers
Table S5. Index of Proteome Discoverer 2.5 search results of FBP-HA_3 co-immunoprecipitation experiments and ProteomeXchange Consortium accession numbers

Figure S1. Box plots of Skp1-SF interactome data
Figure S2. Alignment of *Toxoplasma* F-box sequences
Figure S3. FBXO13 master sequence
Figure S4. FBXO13 gene editing
Figure S5. Peptide coverage in proteomic analyses of FBXO13 and FBXO14
Figure S6. FBXO14 master sequence
Figure S7. FBXO14 gene editing
Figure S8. FBXO1 master sequence
Figure S9. FBXO1 epitope tagging
Figure S10. Localization of FBPs in parasites at low oxygen
Figure S11. Skp1 heavy peptides
Figure S12. nLC/MS analysis of Skp1 modification status

Table S1. Oligonucleotides used in this study.

Name	Purpose	Position in gene	bp cDNA	bp gDNA	Sequence 5'-3' (cloning site)
**FBXO13-related**					
FbxO13 guide R5246 Fw	FbxO13 CRISPR disruption				AAGTTGCGTAGGACCCCAGAGAAGATG
FbxO13 guide R5246 Rv					AAAACATCTTCTCTGGGGTCCTACGCA
FbxO13 homology arm Fw	FbxO13 HA tag insertion				CGTCGACATGCCGACGGGGAGACACCGACAAGCCAGGCAGTGGCCATTGGAAGTGGAGGACGGGAATTCC
FbxO13 homology arm Rv					CTTCTTAGGGGGCGTAGGACCCCAGAGAAGATCGCTTTCTTTCGCGGCAAGAATTGTGTTAACCGGTTCGAC
FbxO13 start Fw	PCR1				ATGGAAGTCGACGAAAGACAACAGA
FbxO13 stop Rv					AGGGAAGACACGAGACGTTCG
FbxO13 scrn Fw	PCR2				GGCCACTGCCTGGCTTG
3HA_R					CACATCATAGGGATAGCCAGCGTAG
**FBXO14-related**					
FbxO14 guide 2549 Fw	FbxO14 CRISPR disruption				AAGTTGAAACATGCAATGTGAGCAAAG
FbxO14 guide 2549 Rv					AAAACTTTGCTCACATTGCATGTTTCA
FbxO14 homology arm Fw	FbxO14 HA tag insertion				CTTTGTCAATTGCGCGTGTACATATGGATTAAACGAGATGTGCTGATTGGAAGTGGAGGACGGGAATTCC
FbxO14 homology arm Rv					GTTCGTGAACATCTTTGGCGTTTGCTCACATTGCATGTTTGACGTGGCAAGAATTGTGTTAACCGGTTCGAC
FbxO14 start Fw	PCR1				ATGGAATTTTTGGACCAGGCTA
FbxO14 stop Rv					CAGCACATCTCGTTTAATCCATATG
FbxO14 scrn Fw	PCR2				AAGGGAGCAAGGAGGTTGTTCGT
3HA_R					CACATCATAGGGATAGCCAGCGTAG
**FBXO1-related**					
FbxO1 219 top	FbxO1 CRISPR disruption plasmid				AAGTTGAACCCCCGTGGAATATCAAG (BsaI)
FbxO1 219 bottom					AAAACTTGATATTCCACGGGGGTTCA (BsaI)
O1 left arm	FbxO1 3×HA tagging PCR amplicon primer				GGCGACATAATCGATTACTACATGAGTCAGGGCGGAGCCAATGCGAAAATTGGAAGTGGAGGACG
O1 right arm					GAGCTGGTGCCCCTGAAACAGCATTGATCCCCAAAAGCGCCGTTGCATCCTGCAAGTGCATAGAAG
O1F1	PCR1				TCTTGACTTGCCTGTACCGAATG
O1R4					GCTCCATGAAACCTTGGTGCAG
3×HA Rev	FbxO1 tagging diagnostic PCR primer				CGGTGATTAGGCATAATCTGG
DHFR Seq For					GACGCAGATGTGCGTGTATCC
FbxO1HA seq for 1 (Seq For)	FbxO1 tagging sequencing primer				TCACGTCGAGCGGTCTTCTG
FbxO1HA seq rev (Seq Rev)					CATAACTCAGTCAGGCAGTC
**qPCR-related**					
EG_TgACT1-F1	ACT1 , TGGT1_209030	676			GAGGAAATGAAGGCGGC
EG_TgACT1-R1		759	84	516	GTTGCCAACGGTGATGAT
EG_TgSkp1-F1	Skp1, TGGT1_207680	277			TGAAGTGGTCAGCGAGT
EG_TgSkp1-R1		328	86	557	GGCTTGATGTTGAGGTAGTTAG
EG_TgFbxO14-F1	FbxO14, TGGT1_259880	300			GATTCTTCTCAATTCGAAGCCAAA
EG_TgFbxO14-R1		380	81	308	GGCCATATGAATCTCGCTC
EG_TgFbxO13-F1	FbxO13, TGGT1_283890	769			AGTCTCTCCCGGTTCTC
EG_TgFbxO13-R1		822	91	226	CGGAAGAACAAGCTGCTAATA
EG_TgFbxO1_F2	FbxO1, TGGT1_310930	1719			CCTTTCTGCCAACTTCGAC
EG_TgFbxO1_R2		1817	99	389	AGCGAGTAAGACACTGTGA
EG_TgbTUB-F1	β-tub, TGGT1_266960	1180			TTCTTGCACTGGTACACG
EG_TgbTUB-R1		1268	89	230	TGATACTCGGACACCAGG

Table S2. Gene Identifiers

Assigned names in this report	Uniprot accession #	Toxo DB gene ID	Toxo DB TGME49/other annotation
SKP1	A0A125YIV4	TGGT1_207680	suppressor of kinetochore protein 1/SCF subunit
CUL1	S7UQH0	TGGT1_289310	cullin-1
RBX1	S7UPA4	TGGT1_213690	ring box protein 1 family protein
PHYa	S7WC35	TGGT1_232960	SKP1 prolyl 4(*trans*)hydroxylase
GAT1	S7W1B4	TGGT1_310400	SKP1-α3-galactosyltransferase
GNT1	S7UM38	TGGT1_315885	SKP1 α-N-acetylglucosaminyltransferase
PGTa	S7UYD5	TGGT1_260650	SKP1 β3-galactosyltransferase/α2-fucosyltransferase
GLT1	S7UUF5	TGGT1_205060	SKP1 α3-glucosyltransferase
FBXL1	S7UXS6	TGGT1_262530	Leucine rich repeat-containing protein
FBXL2	S7W887	TGGT1_313200	F-Box Protein L2
FBXO1	S7W8Z1	TGGT1_310930	F-box protein FBXO1
FBXO2	S7UQ14	TGGT1_215210	F-box domain-containing protein
FBXO4	S7UXG6	TGGT1_228380	hypothetical protein
FBXO5/TPR	S7VU26	TGGT1_243750	Tetratricopeptide repeat-containing protein
FBXO6	S7UYF8	TGGT1_258900	hypothetical protein
FBXO7	S7VW87	TGGT1_275780	F-box domain-containing protein
FBXO10	S7UPY6	TGGT1_215620	hypothetical protein
FBXO11	S7UVP3	TGGT1_203040	hypothetical protein
FBXO12	S7UZJ1	TGGT1_278815	hypothetical protein
FBXO13/JMJD6b	S7UNJ6	TGGT1_283890	histone lysine demethylase JMJD6b
FBXO14	S7UYK1	TGGT1_259880	hypothetical protein
FBXW1	S7UY78	TGGT1_261370	F-box domain-containing protein
PRMT5	S7UQT1	TGGT1_215560	histone arginine methyltransferase PRMT5
Ankyrin	S7UYH5	TGGT1_261230	ankyrin repeat-containing protein
PPI	S7UMS7	TGGT1_283850	peptidyl-prolyl cis-trans isomerase
ACT1	A0A125YH17	TGGT1_209030	Actin ACT1
B-TUB	A0A125YWG4	TGGT1_266960	beta-tubulin
Ubch-2	S7UQR8	TGGT1_289330	ubiquitin carboxyl-terminal hydrolase family 2 protein
His acid phosphatase	S7UUF0	TGGT1_204080	histidine acid phosphatase superfamily protein
5'-3' exoribonuclease	S7UGZ8	TGGT1_305780	5'-3' exoribonuclease, putative
GAP45	A0A125YXT6	TGGT1_223940	myosin motor complex/gliding-associated protein-45
WD_262650	S7UYD6	TGGT1_262650	WD domain, G-beta repeat-containing protein
LRR_311650	S7UTM9	TGGT1_311650	leucine rich repeat-containing protein
TBC_213325	S7W3F3	TGGT1_213325	TBC domain-containing protein TBC18
DNA_helicase_214970	S7W5U3	TGGT1_214970	DNA replication licensing factor MCM2
WD_311870	S7USV5	TGGT1_311870	WD domain, G-beta repeat-containing protein
Unk_267760	S7VVY8	TGGT1_267760	hypothetical protein
Unk_278760	S7UZJ7	TGGT1_278760	hypothetical protein
Unk_244130	S7VTY7	TGGT1_244130	hypothetical protein
Unk_320130	S7UK23	TGGT1_320130	hypothetical protein
Unk_263440/ICAP7	S7UY13	TGGT1_263440	hypothetical protein
Unk_299240	S7VVT8	TGGT1_299240	hypothetical protein
Unk_224470	S7UW21	TGGT1_224470	hypothetical protein
Unk_216840	S7UM73	TGGT1_216840	hypothetical protein

Table S3. Evolutionary analysis of *Toxoplasma* FBPs. Numerical data supporting Fig. 1C.

Numerical data supporting Fig. 1C.			E-values are for protein sequences														
Species	*T.gondii*	*H.hammondii*	*N.caninum*	*B.besnoiti*	*C.suis*	*S.neurona*	*E.tenella*	*C.cayetanensis*	*C.velia*	*V.brassicaformis*	*C.felis*	*P.falciparum*	*P.gigantica*	*C.andersoni*	*C.tyzzeri*	*B.bigemina*	
Strain	GT1	H.H.34	Liverpool	Bb-Ger1	Wien I	SN3	Houghton 2021	CHN_HEN01	CCMP2878	CCMP3155	Winnie	3D7	A	isolate 30847	isolate UGA55	strain BOND	Notes
FbxL1	TGGT1_262530	HHA_262530	NCLIV_025310 *	BESB_001710	CSUI_005396 *	SN3_00800010 *	ETH2_1015100	cyc_01222	Cvel_5628	Vbra_13305							* incomplete sequences make uncertain
E value	0	0	3.00E-151	0	7.00E-86	3.00E-49	3.00E-83	2.00E-86	3.00E-50	4.00E-84							Csuis: F-box is uncertain
aa	1013	955	486, + 199 from genome	920	286	1226	850	764	1868	714							Ncan & Sneu: features found in genome data
FbxL2	TGGT1_313200	HHA_313200	NCLIV_056530	BESB_074190	CSUI_000346	SN3_00501190	ETH2_0402400	cyc_02718 *			CF000850						* missing features found in genome data
E value	0	0	3.00E-80	7.00E-86	4.00E-63	1.00E-38	3.00E-48	5.00E-24			3.00E-21						C.felis F-box is uncertain
aa	843	855	710	796	757	1433	755	: 766 after fixing fbox			423						
FbxW1	TGGT1_261370	HHA_261370	NCLIV_026120	BESB_084810	CSUI_004615	SN3_00301335	ETH2_1131800	cyc_01327	Cvel_1499	Vbra_11669			KVP18_004762				
E value	0	0	0	<<0.000000005	4.00E-178	5.00E-80	4.00E-55	2.00E-72	6.00E-33	4.00E-46			5.00E-17				Bbes Evalue has near perfect fit
aa	1618	1633	1652	1598	2645	2804	1414	1176	1090	557			587				P.gigantica F-box is uncertain
FbxW2	TGGT1_299230 *	HHA_299230	NCLIV_009280 *	BESB_011910	CSUI_003046	SN3_02500440 **	ETH2_0950500	cyc_00968 *	Cvel_26640	Vbra_22338							* C-terminus truncated; ** truncated both ends
E value	0	0	0	0	0	8.00E-169	4.00E-18	2.00E-17	5.00E-75	2.00E-75							
aa	636	1072	574	1135	1293	561	1560	424	1325	1310							
FbxW3	TGGT1_310910 *	HHA_310910	NCLIV_054930	BESB_072410		SN3_00900485	ETH2_0905700	cyc_07509						cand_003350			* missing features found in genome data
E value	0	0	2.00E-49	2.00E-14	not found	2.00E-25	9.00E-49	1.00E-05						2.00E-28			
aa	3144, + 462 from genome	3436	3033	3754		7206	1983	1982						1254			
Fbx01	TGGT1_310930	HHA_310930	NCLIV_054940	BESB_072430	CSUI_009775	sneu_scaffold00009 *	ETH2_1416100	cyc_07722	Cvel_14590	Vbra_21102					CTYZ_00001977	BBBOND_0105760	* found in genome data
E value	0	0	0	0	0		7.00E-88	1.00E-70	2.00E-38	5.00E-41					2.00E-44	2.00E-25	C.tyzzeri and B.bigemina F-boxes uncertain
aa	808	807	779	745	804	1128	764	614	457	541					551	539	
Fbx02	TGGT1_215210	HHA_215210	NCLIV_052180	BESB_034770	CSUI_003167	sneu_scaffold00001 *	ETH2_0416700	cyc_01193				PF3D7_1473400					* Found in genome data
E value	0	0	0	0	2.00E-142		1.00E-31	2.00E-14									
aa	683	682	897	567	439		903	866									
Fbx03	TGGT1_225900	HHA_225900	NCLIV_046930	BESB_060660	MIGC01001652 *	SN3_00102600											* only in genome data, E value listed as it is one exon
E value	0	0	0	0	9.00E-44	3.00E-08											
aa	1461	1467	1330	1441	2014	5692											
Fbx04	TGGT1_228380	HHA_228380	NCLIV_045080	BESB_058550 *	CSUI_002536												* Missing features found in genome data
E value	0	0	0	3.00E-77	3.00E-39	not found											
aa	1726	1726	1741	807	2040												
Fbx05	TGGT1_243750	HHA_243750	NCLIV_018440 *	BESB_014480	CSUI_003940	SN3_00300580 **	ETH2_0722600	cyc_02838									*, ** missing features found in genome data
E value	0	0	2.00E-132	4.00E-116	7.00E-108	2.00E-27	4.00E-33	7.00E-22									
aa	868	865	606 + 393 from genome	816	777	731 + ??aa from genome	608	1259									
Fbx06	TGGT1_258900	HHA_258900	NCLIV_027570 *	BESB_002520	CSUI_009581	SN3_04500060											* missing features found in genome data
E value	0	0	5.00E-134	2.00E-108	4.00E-75	8.00E-10											
aa	577	577	431 + 147 from genome	586	681	852											
Fbx07	TGGT1_275780	HHA_275780	NCLIV_007150 *	BESB_030060	CSUI_002911	SN3_01300160											* missing features found in genome data
E value	0	0	2.00E-175	5.00E-70	1.00E-49	2.00E-21											
aa	979	988	654 + 326 from genome	946	1327	1576											
Fbx08	TGGT1_305630	HHA_305630	NCLIV_070130 **	BESB_017800	CSUI_003046	sneu_scaffold00025 *	ETH2_0803000										* , ** amended from genome data
E value	0	0	5.00E-142	3.00E-126	1.00E-39		1.00E-15										
aa	2165	2214	981 - 46 +1262 from genome	2041	1293		1698										
Fbx09	TGGT1_359350	KL544036 *	NCLIV_008580	BESB_012640	CSUI_003239	sneu_scaffold00004 *											* from genome data
E value	0		0	1.00E-29	7.00E-26												
aa	1788	1771	2137	2808	2383 (very N-term missing)	1715											
Fbx010	TGGT1_215620	HHA_215620	NCLIV_052520	BESB_034370	CSUI_008059 *												* amended with genome data
E value	0	0	2.00E-76	2.00E-44	5.00E-26	not found											No Sneu ortholog with pblast or tblastn
aa	1958	1807	1811	1884	11, + ~ 2500 from genome												
Fbx011	TGGT1_203040 *	HHA_203040	NCLIV_021880 ***	BESB_066130 **	CSUI_000652												*, **,*** missing features from genome data
E value	0	0	0	0	0	not found											No Sneu ortholog with pblast or tblastn
aa	783, + 1112 from genome	1823	1191, -> 1732 from genome	1677, + 94 from genome	1802												
Fbx012	TGGT1_278815	HHA_278815	NCLIV_067190 *	BESB_027470 **	CSUI_010807 ***	SN3_02400215 ****											* , ** missing features found in genome data
E value	0	0	7.00E-46	3.00E-11	5.00E-27	6.00E-21											*** could not be resolved from genome; ****missing features found in genome data
aa	2160	2094	1173 + 180+523 from genome	414 + ??? From genome	2642	1834											
Fbx013	TGGT1_283890	HHA_283890	NCLIV_028900	BESB_076250													Csuis N-term not found; Sneu not found
E value	0	0	0	0													
aa	739	743	761	668													
Fbx014	TGGT1_259880 *	HHA_259880	NCLIV_027010	BESB_003150	CSUI_002895	SN3_01600195											* missing feature found in genome data
E value	0	0	0	0	4.00E-94	6.00E-04											
aa	76 - 103 + 361 from genom	1028	1131	1138	1179	2153											
Fbx015	TGGT1_226850	HHA_226850	NCLIV_046150 *	BESB_059750	CSUI_002964 **	SN3_01300460	ETH2_1422900	LOC34623133 #									* missing exon found in genome; ** missing C-term
E value	0	0	0	4.00E-164	1.00E-70	2.00E-131	4.00E-45	4.00E-46									Ccay and Eth2 F-boxes uncertain
aa	1094	1071	1142 + 21 from genome	1859	1200	1861	473	469									

Table S4. Index of Proteome Dicoverer 2.5 search results of SKP1-SF co-immunoprecipitation experiments and ProteomeXchange Consortium accession numbers.

Skp1_SF RH_PHYA data

	.RAW file names	
control	RH_EC_M2_8uL_24Nov20_1	PhyA_EC_M2_8uL_26Nov20_1
	RH_EC_M2_8uL_24Nov20_2	PhyA_EC_M2_8uL_26Nov20_2
	RH_EC_M2_8uL_24Nov20_3	PhyA_EC_M2_8uL_26Nov20_3
	RH_IC_M2_10uL_09Dec20_1	PhyA_IC_M2_10uL_11Dec20_1
	RH_IC_M2_10uL_09Dec20_2	PhyA_IC_M2_10uL_11Dec20_2
	RH_IC_M2_10uL_09Dec20_3	PhyA_IC_M2_10uL_11Dec20_3
	RH_M2_10uL_13Jan21_1	PhyA_M2_10uL_16Jan21_1
	RH_M2_10uL_13Jan21_2	PhyA_M2_10uL_16Jan21_2
	RH_M2_10uL_13Jan21_3	PhyA_M2_10uL_16Jan21_3
sample	SF_EC_M2_8uL_27Nov20_1	SF_PhyA_EC_M2_8uL_25Nov20_1
	SF_EC_M2_8uL_27Nov20_2	SF_PhyA_EC_M2_8uL_25Nov20_2
	SF_EC_M2_8uL_27Nov20_3	SF_PhyA_EC_M2_8uL_25Nov20_3
	SF_IC_M2_8uL_10Dec20_1	SF_PhyA_IC_M2_10uL_11Dec20_1
	SF_IC_M2_8uL_10Dec20_2	SF_PhyA_IC_M2_10uL_11Dec20_2
	SF_IC_M2_8uL_10Dec20_3	SF_PhyA_IC_M2_10uL_11Dec20_3
	SF_M2_10uL_14Jan21_1	SF_PhyA_M2_10uL_16Jan21_1
	SF_M2_10uL_14Jan21_2	SF_PhyA_M2_10uL_16Jan21_2
	SF_M2_10uL_14Jan21_3	SF_PhyA_M2_10uL_16Jan21_3

PRIDE	accession	PXD050988
	results	RH_PhyA_SF_ori_new_FBP_db.mzid
	peaks	RH_PhyA_SF_ori_new_FBP_db.mzML

date of submission	26-Mar-24
Submission #	1-20240326-153623-1926934

Skp1_SF 4 strains data

	.RAW file name			
control	RH_5ul_in_nanotrap_7Nov22_1	PhyA_IC_M2_10uL_11Dec20_1	gnt1_5ul_21Sep22_1	pgta_6uL_25Aug22_1
	RH_5ul_in_nanotrap_7Nov22_2	PhyA_IC_M2_10uL_11Dec20_2	gnt1_5ul_21Sep22_2	pgta_6uL_25Aug22_2
	RH_5ul_in_nanotrap_7Nov22_3	PhyA_IC_M2_10uL_11Dec20_3	gnt1_5ul_21Sep22_3	pgta_6uL_25Aug22_3
	RH_M2_high_7ul_16Dec2022_1	PhyA_M2_high_5ul_19Dec2022_1	gnt1_M2_high_7ul_18Jan2023_1	pgta_high_02_IC_5ul_07Sep22_1
	RH_M2_high_7ul_16Dec2022_2	PhyA_M2_high_5ul_19Dec2022_2	gnt1_M2_high_7ul_18Jan2023_2	pgta_high_02_IC_5ul_07Sep22_2
	RH_M2_high_7ul_16Dec2022_3	PhyA_M2_high_5ul_19Dec2022_3	gnt1_M2_high_7ul_18Jan2023_3	pgta_high_02_IC_5ul_07Sep22_3
			Gnt1_high_8ul_24Jan2023_1	pgta_5ul_in_nanotrap_9Nov22_1
			Gnt1_high_8ul_24Jan2023_2	pgta_5ul_in_nanotrap_9Nov22_2
			Gnt1_high_8ul_24Jan2023_3	pgta_5ul_in_nanotrap_9Nov22_3

sample	RH_SF_5ul_19Sep22_1	SF_PhyA_IC_M2_10uL_11Dec20_1	gnt1_SF_5ul_22Sep22_1	pgta_SF_B9_M2_7uL_25Aug22_1
	RH_SF_5ul_19Sep22_2	SF_PhyA_IC_M2_10uL_11Dec20_2	gnt1_SF_5ul_22Sep22_2	pgta_SF_B9_M2_7uL_25Aug22_2
	RH_SF_5ul_19Sep22_3	SF_PhyA_IC_M2_10uL_11Dec20_3	gnt1_SF_5ul_22Sep22_3	pgta_SF_B9_M2_7uL_25Aug22_3
	RH_SF_M2_high_7ul_18Dec2022_1	PhyA_SF_M2_high_5ul_20Dec2022_1	gnt1_SF_high_7ul_19Jan2023_1	pgta_SF_high_02_IC_5ul_08Sep22_1
	RH_SF_M2_high_7ul_18Dec2022_2	PhyA_SF_M2_high_5ul_20Dec2022_2	gnt1_SF_high_7ul_19Jan2023_2	pgta_SF_high_02_IC_5ul_08Sep22_2
	RH_SF_M2_high_7ul_18Dec2022_3	PhyA_SF_M2_high_5ul_20Dec2022_3	gnt1_SF_high_7ul_19Jan2023_3	pgta_SF_high_02_IC_5ul_08Sep22_3
			Gnt1_SF_high_10ul_24Jan2023_1	pgta_SF_5ul_in_nanotrap_10Nov22_1
			Gnt1_SF_high_10ul_24Jan2023_2	pgta_SF_5ul_in_nanotrap_10Nov22_2
			Gnt1_SF_high_10ul_24Jan2023_3	pgta_SF_5ul_in_nanotrap_10Nov22_3

PRIDE	accession	PXD050091
	results	RH_2_PhyA_2_Gnt1_3_PgtA_3.mzid
	peaks	RH_2_PhyA_2_Gnt1_3_PgtA_3.mzML

date of submission 23-Feb-24

Submission # 1-20240223-180110-1926934

Skp1_SF PGTA heavy peptide quantification data

	.RAW file name
sample high 02	pgta_SF_high_02_IC_5ul_08Sep22_1
	pgta_SF_high_02_IC_5ul_08Sep22_2
	pgta_SF_high_02_IC_5ul_08Sep22_3
	pgta_SF_5ul_in_nanotrap_10Nov22_1
	pgta_SF_5ul_in_nanotrap_10Nov22_2
	pgta_SF_5ul_in_nanotrap_10Nov22_3
	pgta_SF_high_Nov_5ul_9Dec2022_1
sample low 02	pgta_SF_low_5ul_31Aug22
	pgta_SF_low_5ul_31Aug22_2
	pgta_SF_low_5ul_31Aug22_3
	pgta_SF_low_5ul_nanotrap_12Nov22_1
	pgta_SF_low_5ul_nanotrap_12Nov22_2
	pgta_SF_low_Nov_5ul_8Dec2022_1
	pgta_SF_low_Nov_5ul_8Dec2022_2

PRIDE accession	PXD053703
results	pgta_SF_high_low_2_reps.mzid
peaks	pgta_SF_high_low_2_reps.mzML

Submit date 05-Jul-24

Submission # 1-202040705-220002-1926934

Table S5. Index of Proteome Dicoverer 2.5 search results of FBP-HA$_3$ co-immunoprecipitation experiments and ProteomeXchange Consortium accession numbers.

Fbx01-HA3 data

	.RAW file name
control	RH_0_2NP40_50_5uL
	RH_02_NP40_75_5uL
	RH_1NP40_50_5uL
	RH_1NP40_75_5uL
	RH_1NP40_NaCl_75_8uL
	RH_1NP40_NaCl_8uL
	RH_1NP40_pre_8uL
	RH_1NP40_pre_8uL_75
	RH_IC_50_8uL
	RH_IC_75_8uL
sample	Fbx01_0_2_NP_75_5uL
	Fbx01_0_2NP40_50_5uL
	Fbx01_1NP40_50_5uL
	Fbx01_1NP40_75_5uL
	Fbx01_1NP40_NaCl_8uL
	Fbx01_1NP40_NaCl_75_8uL
	Fbx01_1NP40_pre_8uL
	Fbx01_1NP40_pre_8uL_75
	Fbx01_IC_50_8uL
	Fbx01_IC_75_8uL

PRIDE accession	PXD050011
results	Fbx01_5_Bio_2_Fr.mzid
peaks	Fbx01_5_Bio_2_Fr.mzML

Submit date 21-Feb-24

Submission # 1-20240221-174856-1926934

Fbx013-HA3 data

	.RAW file name
control	RH_5_2uL_07April21.raw
	RH_5_12uL_07April21_1
	RH_5_12uL_07April21_2
	RH_5_10uL_12May21_1
	RH_5_10uL_12May21_2
	RH_5_10uL_12May21_3
sample	Fbx013_HA_12uL_08April21_1
	Fbx013_HA_12uL_08April21_2
	Fbx013_HA_12uL_08April21_3
	Fbx013_HA_10uL_13May21_1
	Fbx013_HA_10uL_13May21_2
	Fbx013_HA_10uL_13May21_3

PRIDE accession	PXD049975
results	Fbx013_Apr_May.mzid
peaks	Fbx013_Apr_May.mzML

Submit date 20-Feb-24

Submission # 1-20240220-203841-1926934

Fbx014-HA3 data

	.RAW file name
control	RH_12_12uL_09April21_1
	RH_12_12uL_09April21_2
	RH_12_12uL_09April21_3
	RH_12_8uL_15May21_1
	RH_12_8uL_15May21_2
	RH_12_8uL_15May21_3
sample	Fbx014_HA_12uL_09April21_1
	Fbx014_HA_12uL_09April21_2
	Fbx014_HA_12uL_09April21_3
	Fbx014_8uL_17May21_1
	Fbx014_8uL_17May21_2
	Fbx014_8uL_17May21_3

PRIDE accession	PXD049694
results	Fbx014_Apr_May.mzid
peaks	Fbx014_Apr_May.mzML

Submit date 19-Feb-24

Submission # 1-20240219-172527-1926934

Figure S1. Proteomics data for Skp1-SF interactors. The box plots show all data, the mean, and the 95% confidence interval. Peptide abundances calculated by Proteome Discoverer 2.5 were summed to represent protein abundance from 3 biological replicates, each with 3 technical replicates. Comparison of samples were from spontaneously lysed extracellular parasites, syringe-lysed out intracellular parasites, or transitional cultures containing 50% extracellular and 50% intracellular parasites all trended similarly, so were pooled for quantitative analysis. Proteins named in black were detected with ≥3 peptides (except FBXO6, GAP45, and His acid phosphatase, 2 peptides), and enriched >5-fold (FC, $p<0.05$ using Metaboanalyst) based on co-IPs from Skp1-SF tagged strains (labeled sample) relative to untagged strains (labeled control). Proteins were named in green if enriched in tagged RHΔΔ relative to tagged PHYaΔ cells more than 2-fold (at $p<0.05$). *A*, Interactors identified with high (FDR<0.01) or medium confidence (0.01<FDR<0.05). *B*, Interactors detected with low confidence ns = non-significant. See Table 1 for summary, and Table S4 for access to the raw data.

Figure S1A

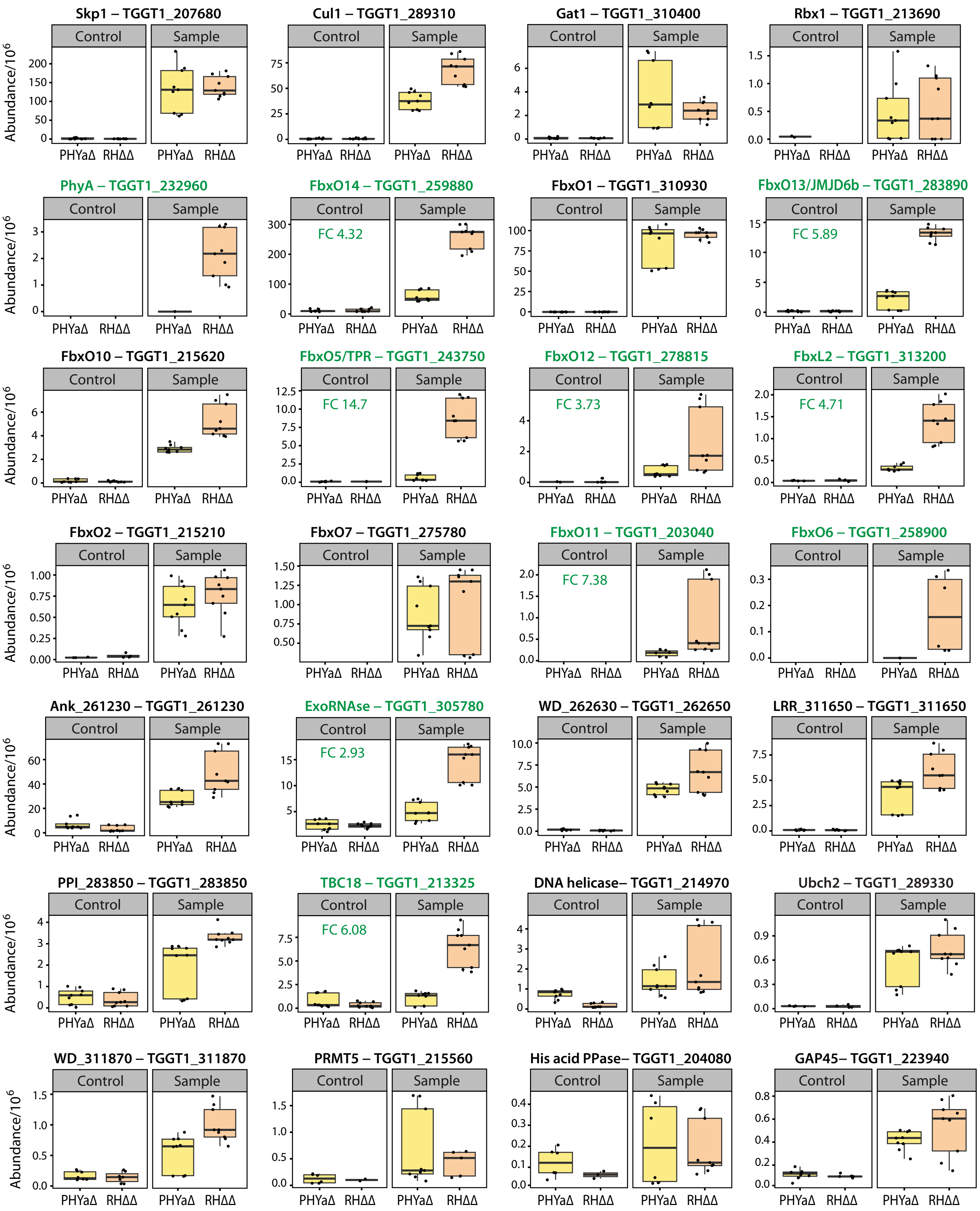
Skp1 – TGGT1_207680
Cul1 – TGGT1_289310
Gat1 – TGGT1_310400
Rbx1 – TGGT1_213690
PhyA – TGGT1_232960
FbxO14 – TGGT1_259880
FC 4.32
FbxO1 – TGGT1_310930
FbxO13/JMJD6b – TGGT1_283890
FC 5.89
FbxO10 – TGGT1_215620
FbxO5/TPR – TGGT1_243750
FC 14.7
FbxO12 – TGGT1_278815
FC 3.73
FbxL2 – TGGT1_313200
FC 4.71
FbxO2 – TGGT1_215210
FbxO7 – TGGT1_275780
FbxO11 – TGGT1_203040
FC 7.38
FbxO6 – TGGT1_258900
Ank_261230 – TGGT1_261230
ExoRNAse – TGGT1_305780
FC 2.93
WD_262630 – TGGT1_262650
LRR_311650 – TGGT1_311650
PPI_283850 – TGGT1_283850
TBC18 – TGGT1_213325
FC 6.08
DNA helicase– TGGT1_214970
Ubch2 – TGGT1_289330
WD_311870 – TGGT1_311870
PRMT5 – TGGT1_215560
His acid PPase– TGGT1_204080
GAP45– TGGT1_223940
Control
Sample
PHYaΔ
RHΔΔ
Abundance/10^6

Figure S1A (cont.)

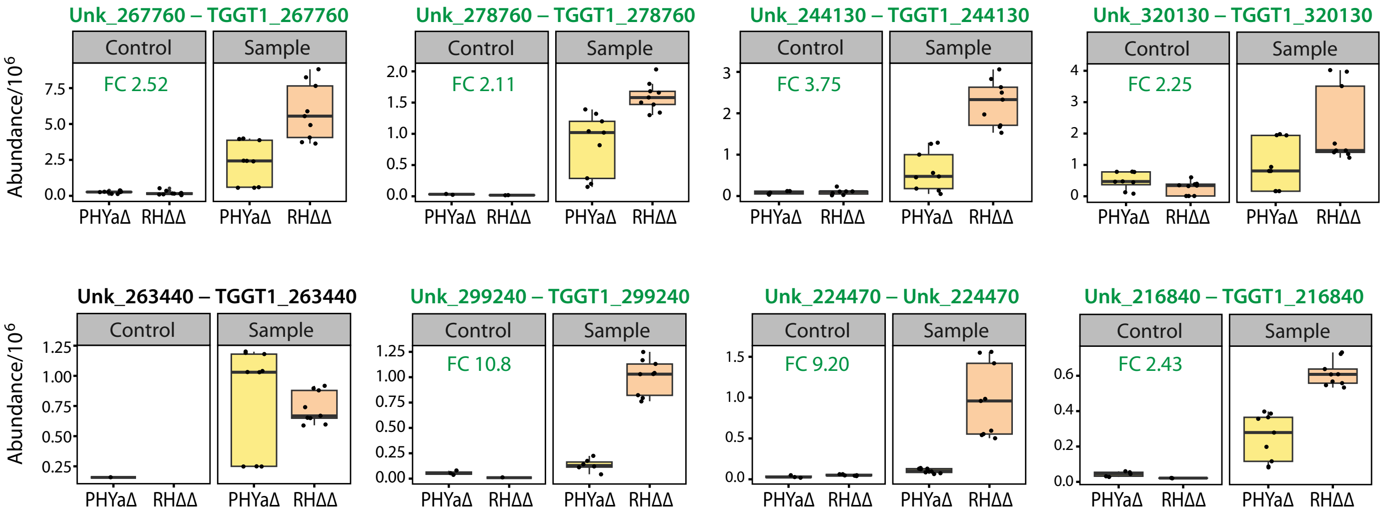

Figure S1B

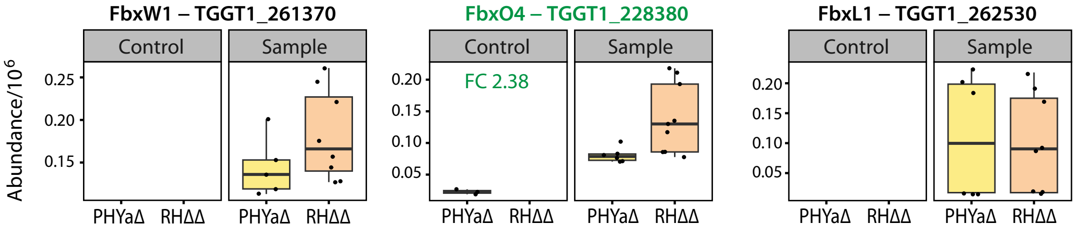

Figure S2. Alignment of predicted F-box sequences of candidate *Toxoplasma* F-box proteins, updated from ref. 19 (Fig. S1C). Sequences were retrieved using BLASTp searches seeded with various F-box sequences from yeast, *Dictyostelium*, and *Arabidopsis*, NCBI domain searches, and hidden Markov models. Inserts were removed as indicated. To facilitate visualization of relatedness, acidic residues are in blue, basic in dark red, Gly and Pro in red, and hydrophobic in green, as previously described (40). Positions possessing related chemical characteristics are highlighted in yellow (hydrophobic), gray (acidic), dark grey (basic), or teal (small). Residues identical to the consensus sequences are bolded. Potential substrate receptor domains and other domains are tabulated on the right.

Name	Gene ID*	Length a.a.	Start	Sequence‡	domains
FbxW1	TGME49_261370•	1618	941	WTSVPAEVLSTLCQFL-AVEDLVAFQRL-DRRAYAVGSH--ATVWRAL	WD40
FbxW2	TGME49_299230+	1101	16	PACLPTAIVFHVASFL-TPNDVCSMGAT-CRKWREVCCSDLQPLWREF	WD40
FbxW3	TGME49_310910	3700	248	LESLSPCLLANLVSFL-LPLDIVSVSLC-SRSLLWLSRC--PFVWRRC	WD40
FbxL1	TGME49_262530•	979	173	FAELPQEVLELIFSRL-GLADLSRCLCV-AKSWHPPLNA---VFAKTI	LRR
FbxL2	TGME49_313200•	832	307	LSDLPEELLQQILDCC-PKECLLVSHAL/RRRVLRLTPL---QCTEPR	LRR
FbxO1	TGME49_310930•	808	434	FPFLDEPALSLLVPFL-FGRSLATCMTV-CPHWFMKINR---AMERMC	AF4
FbxO2	TGME49_215210+•	683	254	FNTCPAECLQAVFHFL-HVEDILRMQVV-SSAFFSTIRDEIGAFTHIR	
FbxO3	TGME49_225900+	1461	160	LEALPAGCMYTLFAFF-DVSEVAELRLL-SRTVKAVVDS--PCSLRGC	
FbxO4	TGME49_228380¶•	1726	31	LETPEPLCFDYLLSFL-DLRDFLTLSLV-SHSLRDILLSDLTRAARCV	
FbxO5	TGME49_243750+•	865	478	LCSLPQELLDVLPLYL-DAFALTRLSSC-CRLLHRLCGNRSDVCWRAK	TPR
FbxO6	TGME49_258900¶•	577	3	DLLQHPDIVGRILSCL-AWRERRQLAAV-CVSWREAAET--SPCWSDL	
FbxO7	TGME49_275780•	978	211	IDALPDDLLCEMLLFL-PFDEVGASIPLVSRRFCRLALL--PYIWTFF	
FbxO8	TGME49_305630	2045	61	SPDGPCETIVFLLGFFLPVADLCSCSAV-CKAWWAVCTLQHQQLWRER	
FbxO9	TGME49_500384¶	3034	742	LRQLPPAILCAVLRFL-PCASVLAFGTT-CRYAHDLVQL--PAAWNLL	
FbxO10	TGME49_215620¶•	1930	220	GAEAPEDARGCLLAFL-TWNDLARLRAV-SRQLKRLVEN-AALSARAV	
FbxO11	TGME49_203040•	1824	240	FSDLPDVCVRICFSFL-SVEEILKYQFL-SRYIRRAIGL---DHVLPL	
FbxO12	TGME49_278815¶•	2447	361	LHLLPSSALSLLLRFL-HLDDVCRLALS-SKQLYLHPDLNTPFAVAHL	LeuZip
FbxO13	TGME49_283890•	739	63	FAALDDAAFLAFLSTL\PLSALLSLSCA-SKFLLAALLDE--ELWQSL	JmjC
FbxO14	TGME49_259880•	1034	1	MEFLDQATLATLATFL-LSNELPKLRLL-SKALNGRLFP--VDSFRRL	

F-box motif consensus sequence¢ #xxLPxEILxxILs#L-xxxaLLxLsx#-Cbx#xxLxxx--xx#wbxl

*Sequence identifiers are from www.toxoDB.org/.
‡ Acidic residues are in blue, basic in dark red, small in red, and hydrophobic in green. Positions matching the consensus motif are highlighted in yellow (hydrophobic), gray (acidic), green (basic), or teal (small). Bolded residues correspond to the PxE motif.
+= reported also in ref. 6
¶ = Identified by hidden markov model
• supported by interactome studies
/ = AAAVRR; \ = AEFV; LeuZip=leucine zipper
¢ Consensus: #=hydrophobic side chain, s=small, b=basic, a=acidic, PxE motif is bold

Fig. S3. DNA and protein master sequence of FBXO13, derived from TGGT1_283890, is annotated as JMJD6b at Toxo.db. Coding nucleotides are upper case. Potential nuclear localization sequences (41) are in blue, the predicted F-box sequence (10) is in dark red, the jumonji C domain is in green, the PAM sequence is in violet, and the modification of the PAM sequence in the repair DNA is in red. Oligonucleotides utilized in modifying and verifying modifications of FBXO13 are represented.

```
ttccgtttctctgggcgttatccttcctcttcattcctccactggcttctccgacggaac
ttttcttcttctgtctgcagtctttttcgctttttctttttttgcgtccatgcgtggagg

M  E  V  D  E  R  Q  Q  T  R  V  A  F  D  P  G  R  K  K  N     20
ATGGAAGTCGACGAAAGACAACAGACGCGGGTCGCTTTTGATCCAGGAAGAAAAAAGAAC   60

L  K  K  K  K  P  R  L  L  L  T  K  H  P  F  G                 36 potential NLS
CTGAAAAAGAAGAAGCCTCGACTCCTCTTGACAAAGCATCCTTTCGGCgtaagttatatg  120

Tttgtgtgtttttcttccgatcaccatcgatgcctgtcgactcccgacttcagaaggaga  180

gactgccttctttcacctcccaggaccacagttgtttttctccctgccttcacacccttc  240

ttgcgtcatctcctgtgtgattcttcagctcgtccagagtttctccctttcttcgtctct  300

gtcgctcttctcatttcttttgtgcaacttgttcgcctctccagtctgatacggttttgt  360

tcatcctctttctccttcccgctccgtttcctttactctttaactctctctctcttca  420

aacggaggagtgtgtgtcgttacttgatttctgtgcttgacttctgatccatccgtatac  480

aagagttctctaccttttcgtatttttaggtctccttttctgtgacgaccttgttgccg  540

ccgtaccctcgtatcttctgcactttctttcttttcctttttacaagtcttttcctgttc  600

tttttctccttgcttaacctgtgcttggccgtttgcgtcattattctttctgcgttgcct  660

                           L  L  P  S  G  N  A  L  F  V  S  S     48
ccgtttcccctgaccctccactgcagTTGCTGCCTTCAGGTAACGCGCTCTTTGTCTCTT  720

  G  G  S  T  V  V  D  R  S  A  S  L  G  S  F  A  A  L  D  D   68 F-box domain
CTGGCGGCTCGACGGTGGTGGATCGCTCGGCGTCGCTCGGGAGTTTCGCGGCTCTGGACG  780

  A  A  F  L  A  F  L  S  T  L  A  E  F  V  P  L  S  A  L  L   88
ACGCAGCGTTCCTCGCTTTCCTCTCGACTCTCGCCGAATTCGTTCCGCTCTCTGCTCTTC  840

  S  L  S  C  A  S  K  F  L  L  A  A  L  L  D  E  E  L  W  Q  108
TCTCGCTCTCCTGCGCCTCCAAGTTCCTTCTCGCTGCTCTCCTCGACGAAGAGCTGTGGC  900

  S  L  L  L  S  R  Q  Q  R  R  V  G  R  E  E  S  G  A  R  G  128
AGTCTCTCCTTCTCTCGCGACAGCAACGGCGAGTGGGCCGGGAAGAGAGTGGAGCGAGAG  960

  L  G  A  R  E  H  G  E  Q  G  T  R  K  N  G  E  T  L  L  G  148
GTCTCGGAGCGAGAGAACACGGTGAACAAGGAACGAGGAAGAATGGAGAGACTCTCCTAG 1020

  T  A  D  P  L  L  L  S  D  S  A  S  P  S  A  P  S  P  P  S  168
GGACAGCGGATCCTCTTCTTCTCTCTGACTCTGCTTCTCCTTCTGCTCCCTCTCCTCCTT 1080

  L  S  S  S  F  S  A  C  S  E  E  G  E  T  P  T  K  D  E  T  188
CTCTCTCTTCTTCTTTCTCGGCTTGTTCGGAGGAAGGCGAGACGCCGACAAAGGACGAGA 1140

  S  E  S  T  D  F  T  W  R  G  S  W  K  K  T  Y  L  F  A  E  208
CGTCTGAGTCGACCGACTTCACCTGGCGCGGCAGTTGGAAGAAAACCTACCTGTTCGCAG 1200

  R  E  R  L  T  R  Q  R  T  A  R  A  S  S  S  S  S  S  S  A  228
AACGAGAGAGACTCACGCGCCAAAGGACAGCCCGCGCGTCTTCGTCCTCTTCTTCTTCGG 1260
```

```
  C  S  T  Q  E  E  R  S  A  D  T  C  L  D  T  A  G  D  S  V  248
CTTGCTCAACGCAGGAAGAGCGCTCGGCAGACACCTGCCTAGACACCGCAGGCGATTCCG 1320

  D  R  Q  S  L  P  V  L  R  G  V  C  S  D  T  F  Y  Q  R  W  268
TCGACAGGCAGTCTCTCCCGGTTCTCCGCGGAGTCTGTTCTGATACTTTTTACCAACGAT 1380

  L  C  A  T  V  D  I  S  S  L                                278
GGCTTTGCGCGACTGTCGATATTAGCAGCTTgtaagtttttcactttccgacacaaaaac 1440

gtttcctttctcgacagactgtgcctcttcttgacgtcaacgcggcaacgacgaactctc 1500

tgctctgggcaggcagcctgtagaagaagcggcacatccgttgctctgtcttccatctcc 1560

                                                          F   279
tgcctctccttgcatctggccttcttgactgtggggcctctcggctctctcttcagGTTC 1620

F  R  H  Y  D  N  L  E  R  V  S  A  S  A  L  S  V  D  A  F    299
TTCCGCCACTACGACAACCTCGAGAGAGTCTCTGCCTCGGCATTGAGCGTCGACGCGTTC 1680

V  E  L  Y  E  K  P  N  K  P  V  V  I  T  D                   314
GTTGAGCTCTATGAAAAACCAAACAAGCCGGTCGTCATCACAGgtccgtccactcctcaa 1740

ctcaaaactggagcctcaagacggtgtagacagtgcagactcaactgtggccgtcggtgc 1800

atctaagcggaaagtcggctgaatacgtcgaacgcgaacttccagactgaacatcgctgt 1860

catttgaaccaaaccttctttcttttgccatatgcgcaaattcgggtggaactttttgtg 1920

acgttgccttcccgcgttcgttcgctgcttcctcctggaagaacagcgaaaccgttctcg 1980

agctgcgcaccaagcttccatctaaaggaatgtttctctcctgcccagtgcctttctctc 2040

aagaggctacgactccgctgcggtgtcgcttgctgcatcgaacagccggagctaaatcca 2100

ggacttcttctgcttctcggctggtcgcacatgcgccgacgactcacacattcccatacg 2160

agtgcatgcatgcagctgtgtcggcatacactcaagaagagtcatatgtatctacgtacc 2220

ctggtatactcccctttacgcgtccggaagcagggacatgttccacttgttgaaagcttt 2280

atacctgctcatatatttatactcttccctgagcaccctcagacggccagttgccagtac 2340

gcctatcttcacatactagtgcatgcatctatctgagtacatccacatacagatgtatat 2400

atgtatacatgtatacatgtatatatgtatatatgtgtatttgtgtagtcacctcctgct 2460

caggggtccagatgtctacagggacagatgtgtactgtttctatgttttaatttttttcg 2520

                                      L  V  P  K  W  A  A  F    322
tccacatgcatttttctgttctatttgtttgtagATCTGGTGCCCAAGTGGGCCGCCTTC 2580

G  K  W  N  G  E  Y  F  R  R  H  F  G  G  V  R  F  N  A  G    342
GGGAAGTGGAATGGAGAGTACTTTCGCCGTCATTTTGGCGGCGTGCGTTTCAACGCCGGG 2640

A  A                                                           344
GCTGgtgaggctgtgaacgcgtttcgtctttggaaggaataaaccactgtgctttgtgtg 2700

tctcgaatctcaataatatcagacctccgttctccgttgccgcagcttgcggtagaacca 2760

ggtttctgggaggccttccgtccactgcggcactcttggttctcgccgttatgtccttc 2820

gtccgtcttccttcttggctgacctcctgccctttgttttcccgcttttccgtggatct 2880
```

```
ccctgtctcgcttttaacgactttttccgctcggatttccccccacaactgcgcatgctt 2940

ccgcccctgttgacgattctctgactcttgcgtttttttgcgtcgatttctctgtcgcctg 3000

                           S  N  I  Q  L  E  T  F  Y  Q  Y  A   356
ctgcctgctgttgcgctcaacagCCTCCAACATACAGCTCGAGACCTTCTACCAGTACGC 3060

 D  S  N  F  D  E  A  P  L  F  I  F  D  P  R  F  A  E  S  T   376
GGACTCCAACTTTGACGAAGCGCCTCTCTTCATCTTTGATCCGCGCTTTGCTGAATCGAC 3120

 R  E  A  L  S  S  S  S  A  L  S  S  P  S  S  S  S  P  V  N   396
TCGAGAGGCTCTCTCGTCTTCATCAGCGTTGTCGTCACCTTCTTCTTCTTCTCCTGTGAA 3180

 V  P  P  A  S  R  E  I  G  E  Q  G  D  C  R  R  E  T  T  G   416
CGTTCCTCCGGCTTCGCGGGAGATAGGGGAACAAGGCGACTGTCGGCGAGAGACGACGGG 3240

 A  Q  A  S  A  E  E  R  R  H  H  E  L  G  D  R  V  C  S  L   436  JMJC domain
AGCACAAGCGTCGGCGGAAGAAAGGCGTCATCACGAACTCGGTGACAGAGTGTGCTCGCT 3300

 A  E  D  Y  E  V  P  P  Y  F  S  D  S  R  D  L  F  A  C  L   456
GGCCGAGGACTACGAAGTCCCTCCGTACTTTTCAGACTCTCGCGACCTCTTTGCGTGTCT 3360

 G  E  R  R  P  N  F  R                                        464
TGGAGAACGCAGACCCAATTTCAGgtgcgttgccttcctttctcttctttttcttcttc 3420

tcttgctctcgtttttctgttcctgactatctttcgccaactcttcctcgcctttcctct 3480

                        W  L  L  V  G  N  C  R  S  G  S  K  W   477
ccgcgctctccgctgtgcagGTGGTTGCTCGTGGGGAACTGCCGCTCCGGATCGAAATGG 3540

H  V  D  P  N  Q  T  S  A  W  N  A  V  V  R  G  A  K  R  W    497
CATGTTGATCCGAACCAGACGAGTGCCTGGAACGCCGTGGTGAGAGGCGCCAAACGGTGG 3600

I  L  L  P  P  T  V  C  P  P  G  V  F  P  S  H  D  G  G  E    517
ATTCTTTTGCCTCCGACTGTCTGTCCGCCTGGCGTCTTTCCCTCGCACGACGGCGGCGAA 3660

V  T  Q  P  T  A  L  V  E  W  L  M  N  Y  Y  F  D  A  L  H    537
GTGACGCAGCCCACCGCCCTCGTCGAGTGGCTCATGAACTACTATTTCGACGCACTGCAT 3720

A  P  G  Y  P  Y  T  G                                         545
GCGCCAGGATACCCGTACACCGgtaggcgagcaagcggcagaagcgaaaagaaaggaaaa 3780

gaagaccgagcaatcgaggagagcgcgagggaactcgcgcgacagaaagacggagtggaa 3840

aggacgaggaaggacctgcgcagcgaaggacttgaactcgaggggacggagaacgcgaca 3900

tgtgacgggaagggaggagaagtgaggacgtaaaaaacagtggagagaaaaagcagattc 3960

gtttgtgggggtttcaaggttctaggcgcttcgtttccgagtcgcctcacatcttcttaa 4020

                          G  I  A  P  I  E  G  S  V  R  E  G  557
aacgggatttatgtttcttgtcagGAGGCATTGCCCCCATCGAGGGCTCTGTTCGCGAGG 4080

  E  L  I  F  V  P  Q  G  W  W  H  C  V  L  N  E  E  D  D  T  577
GAGAGCTCATCTTCGTTCCTCAAGGATGGTGGCACTGCGTGCTCAATGAGGAAGATGACA 4140

  I  A  V  T  Q  N  F  V  S  P  V  I  L  Q  N  V  R  S  F  L  597
CGATTGCCGTCACCCAGAACTTCGTCTCGCCTGTTATCCTTCAAAATGTTCGATCTTTTC 4200

  H  Y  K  K  D  Q  I  S  G                                     606
TCCACTACAAAAAAGACCAAATCTCCGgtaacttcacactcctcactccctattggctta 4260
```

```
tttatttactttttatttatgtacttgatcatttgtttgctcgcttgtttatttactta 4320

ctcacttgtttccttatttattggcatgctcatttatttctctatttaattactcattcg 4380

gcttgccgagttatctgtttttacatgtagttgccttcgcttcgtatctccgtctcttt 4440

attttcacctttctcccgatgcatttgtctccagaactctgtgtatcacggggtggacat 4500

caagctgtggcaacgccctcggcggagtcgatgccacccggctacagatctctgtatttg 4560

cgtctgcgtttttcgctcttttcgaccgtttacgcgaaactccgaccgctgcggaggccg 4620

gtgtgtttttgcaaaaggcggaacgccgtcgggaggtttttatgctgtagatgtcgcct 4680

taaatccgccttggggagctgacggctttgcctccgacaccgggagggagtgctttgttt 4740

ccttcgtttccttcgtttgcttccttttctttcttttctttctcaacactttgggaaagg 4800

                                                    L  C  A   609
cgtctctctcctcgacgaggacgcttcgtttttctgcgtcgctgctttcagGCCTCTGTG 4860

  Q  G  R  H  E  T  F  A  S  E  F  D  A  A  V  G  A  S  Y  P  629
CTCAGGGAAGACACGAGACGTTCGCGAGTGAGTTCGACGCCGCGGTCGGAGCTTCTTACC 4920
5'-agggaagacacgagacgttcg                                          FBXO13 scrn Fw

  E  L  L  P  L  V  S  S  P  P  P  P  C  P  S  S  S  P  S  S  649
CGGAGCTGCTGCCTCTCGTCTCTTCGCCTCCCCCTCCATGTCCCTCTTCTTCTCCTTCTT 4980

  P  S  P  V  S  R  T  A  L  R  S  S  R  P  K  E  T  C  G  E  669
CTCCTTCGCCTGTGAGTCGGACGGCGCTGAGGAGCAGTCGTCCGAAGGAGACTTGTGGAG 5040

  E  Q  K  G  V  E  R  G  G  G  Q  C  G  E  P  Q  D  I  R  E  689
AGGAGCAGAAAGGCGTCGAGAGGGGAGGGGGGCAATGCGGAGAGCCACAGGACATAAGGG 5100

  Q  T  D  G  E  G  R  E  G  T  K  R  N  Q  E  P  Q  D  S  G  709
AACAGACAGACGGCGAAGGCAGAGAAGGAACAAAGAGGAACCAAGAGCCACAGGACTCAG 5160

  S  F  W  E  W  L  K  K  R  R  R  P  V  V  L  R  R  H  A  D  729 potential NLS
GAAGCTTCTGGGAGTGGCTGAAGAAGAGACGGAGACCGGTGGTTCTACGTCGACATGCCG 5220
                                             5'-cgtcgacatgccg     Fw homology arm

  G  E  T  P  T  S  Q  A  V  A  *                             739
ACGGGGAGACACCGACAAGCCAGGCAGTGGCCTAGgcgaaagaaaccgatcttctctggg 5280
acggggagacaccgacaagccaggcagtggcc                                  Fw homology arm
                                3'-cgctttctttgcctagaagagaccc      Rv homology arm
                                        3'-tggctagaagagaccc      gRNA sequence

gtcctacgccccctaagaagacatccaatgccggagtcgactgtccaccggatttgtttg
caggatgcgggggattcttc-5'                                           Rv homology arm
caggatgc-5'                                                       gRNA sequence

tttcattgctggattttgctctgtttttctcaactttctccccttgtgtccctgggtc
```

Figure S4. HA3-epitope tagging of FBXO13/JMJD6b (TGGT1_283890). The positive result for PCR2 in the edited strains is consistent with the resistance of the strains to growth in the presence of chloramphenicol. The positive result for PCR1 indicates that the remainder of the locus is intact.

A. Editing strategy

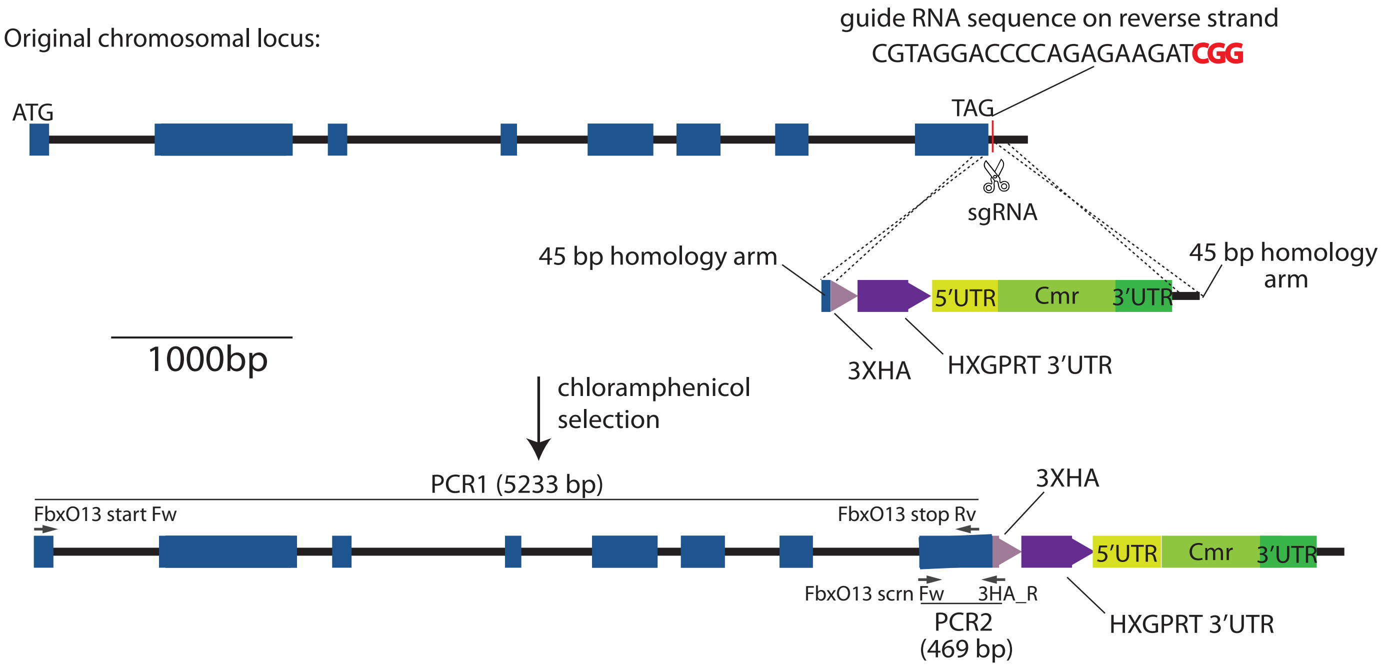

B. PCR assessment

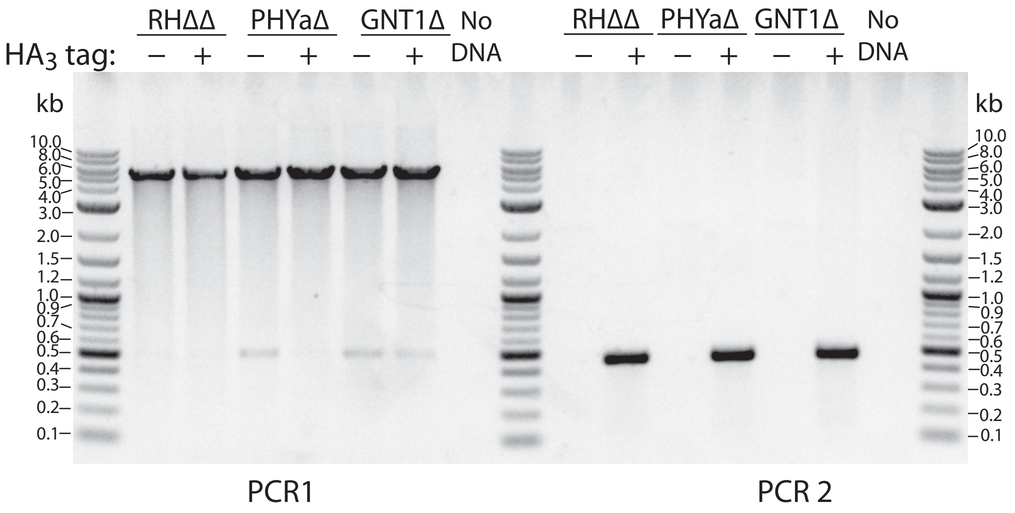

Figure S5. Peptide coverage of FBPs. *A*, Peptides found only in co-IPs of FBXO14-HA$_3$ (yellow) or SKP1-SF (blue) or both (green). *B*, Peptides found only in co-IP of FBXO13-HA$_3$ (yellow) or SKP1-SF (blue) or both (green). See Fig. S8 for peptides found for FBXO1.
Found Modifications: A, acetyl (N-term); C, carbamidomethyl (C); D or E, deamidated (N or Q); O, oxidation (M)

A) FBXO14

```
A    E
MEFLDQATLA TLATFLLSNE LPKLRLLSKA LNGRLFPVDS FRRLIREPRS LVFPAEWGQE KLESVKNRSK REQLFEEGSQ    80
  D                                              D                                  E
RAQGGAVPEA SEVHSVTASP PVSLDIPVSS VAPSRRDFNY LRVLVNLPPP PPHLLHLTLH IPENLRTVTR SWLRTYHQLL   160
C          E              O            D     C                       E D E      C C
CVAAPHLETL QLVEFFSARL RKDLMVRDGA VHAEDKNGDA SCSADTSNPR ESIPESDAVA PENQENRQEE APVCSCPWRA   240
                                    C                     C
PKKHEALPLP AFPRLRFLAV RNRGGWSVRP PLPCAFTYLF LGDLQTGDSS RESCWASSES TTPSEAFPVL KDIRFLEFTT   320
                                                                                    C
IGLDLFRQFL VKHNVTSAEH IAIGMWHMSP LQDFLLFLVR ENSKPKRRFH KLRSLDVHGA RFIWPPDEFW YFFWRSWCDF   400
                             C       C       C  E                 C
TQIWKESFLQ RSSDSDRGDA RRRASALCDD HWTSCPKDWS TCPDQWGTDN DPLLRVSDGD CEPQDEDLVE EGGSINDEFL   480
                        C
SDAGEDEGKD RESIVRMLKR LPCLRRVLLS GFVGTFVAEE ADEAIRFFED IFPQAVVDVE GEIIVSTNAL LEAVVQATAH   560
                                                     D       D                        D
PGWASRLDSF MARKREHRAQ VTQATGSRAS PWTEDFDRSG VTAAAVGAPN SEVSTSNAEL ERDDESEHLA LEGRRYDERN   640
                                                        C      C        O      E
WEALDGVFLS ERGKENFGRW LALGVIEDVY IEVSNPTYPK VHLQINAFHR YCVSTYFKCL ELPATKMEDP VGQDWGLPSV   720
                                 D                  C         C                C
TVRHVVQWGT KYVQDFLDIV STFRHVILNL NYIFWTDRGE YVDANAERCL GSEAGEACRT PSEGNLIQSD APCARTDGRA   800
               E                      EC
QDVHDVAKAD FSVDQESGET DAEPPPVVSA DTRFLQCSES ASPADGASIR SSDAGDERLV ASDRTAHQGS KEVVRLGEFD   880
    D                                          D    O              O
GHFPNVVGLL LPLYWMDDAT WSTDEIARIA EKYRSQTQVV RIKNLFPIMG EYATVDDPVS LMARDFEIVT RICKDTMRVI   960
                                                  O E             C
DYRVYSFFSW KDELGLDENT LIPQFLKEFV SNNPEFTLAK RLDSPSMTQT GVVTVGQEAL CQLRVYIWIK RDVL        1034
```

B) FBXO13

```
MEVDERQQTR VAFDPGRKKN LKKKKPRLLL TKHPFGLLPS GNALFVSSGG STVVDRSASL GSFAALDDAA FLAFLSTLAE    80

FVPLSALLSL SCASKFLLAA LLDEELWQSL LLSRQQRRVG REESGARGLG AREHGEQGTR KNGETLLGTA DPLLLSDSAS   160
                                                                          C           C
PSAPSPPSLS SSFSACSEEG ETPTKDETSE STDFTWRGSW KKTYLFAERE RLTRQRTARA SSSSSSSACS TQEERSADTC   240
                    C
LDTAGDSVDR QSLPVLRGVC SDTFYQRWLC ATVDISSLFF RHYDNLERVS ASALSVDAFV ELYEKPNKPV VITDLVPKWA   320
                                                                                  D
AFGKWNGEYF RRHFGGVRFN AGAASNIQLE TFYQYADSNF DEAPLFIFDP RFAESTREAL SSSSALSSPS SSSPVNVPPA   400
                                    C                      C                 C
SREIGEQGDC RRETTGAQAS AEERRHHELG DRVCSLAEDY EVPPYFSDSR DLFACLGERR PNFRWLLVGN CRSGSKWHVD   480

PNQTSAWNAV VRGAKRWILL PPTVCPPGVF PSHDGGEVTQ PTALVEWLMN YYFDALHAPG YPYTGGIAPI EGSVREGELI   560
                                                   C
FVPQGWWHCV LNEEDDTIAV TQNFVSPVIL QNVRSFLHYK KDQISGLCAQ GRHETFASEF DAAVGASYPE LLPLVSSPPP   640
 C                          C               C   E
PCPSSSPSSP SPVSRTALRS SRPKETCGEE QKGVERGGGQ CGEPQDIREQ TDGEGREGTK RNQEPQDSGS FWEWLKKRRR   720

PVVLRRHADG ETPTSQAVA                                                                      739
```

Fig. S6. DNA and protein master sequence of FBXO14, derived from TGGT1_259880 at Toxo.db. Coding nucleotides are upper case. The predicted F-box sequence (10) is in dark red. Oligonucleotides utilized in modifying and verifying modifications of FBXO14 are represented and listed in Table S1.

```
tctttcagcgttctggcagtaacttgtgagatgaaataattctcctcgtgtgggactgga
cgactcttgcggaatcgacatctcagggctcagccgtggatttgtgtcattccggctgag


M  E  F  L  D  Q  A  T  L  A  T  L  A  T  F  L  L  S  N  E     20  predicted F-box domain
ATGGAATTTTTGGACCAGGCTACTTTGGCCACCCTGGCCACCTTTCTTCTTTCCAATGAG    60

L  P  K  L  R  L  L  S  K  A  L  N  G  R  L  F  P  V  D  S     40
CTGCCCAAGCTCCGGCTTCTCTCTAAAGCACTCAATGGTCGTCTTTTCCCCGTTGACTCG   120

F  R  R  L  I  R  E  P  R  S  L  V  F  P  A  E  W  G  Q  E     60
TTTCGGCGGCTTATACGAGAGCCGCGATCGCTGGTCTTTCCAGCCGAATGGGGACAAGAG   180

K  L  E  S  V  K  N  R  S  K  R  E  Q  L  F  E  E  G  S  Q     80
AAACTGGAAAGCGTGAAGAATCGCAGCAAGCGTGAGCAGCTTTTCGAGGAAGGAAGCCAA   240

R  A  Q  G  G  A  V  P  E  A  S  E  V  H  S  V  T  A  S  P    100
AGAGCGCAAGGCGGTGCTGTGCCAGAGGCGTCGGAGGTCCACAGCGTGACTGCGTCCCCG   300

P  V  S  L  D  I  P  V  S  S  V  A  P  S  R  R  D  F  N  Y    120
CCGGTTTCCCTGGACATACCGGTTTCATCAGTTGCCCCTTCCAGACGAGATTTTAACTAC   360

L  R  V  L  V  N  L  P  P  P  P  P  H  L  L  H  L  T  L  H    140
CTTCGGGTTCTGGTGAACTTACCCCCCCCACCGCCGCATTTGCTGCACCTGACTCTTCAC   420

I  P  E  N  L  R  T  V  T  R  S  W  L  R  T  Y  H  Q  L  L    160
ATCCCGGAGAATTTACGCACAGTAACGCGTTCGTGGCTGCGAACCTACCATCAGCTTCTG   480

C  V  A  A  P  H  L  E  T  L  Q  L  V  E  F  F  S  A  R  L    180
TGTGTGGCGGCTCCTCACCTGGAAACTCTGCAGCTCGTCGAGTTTTTCTCAGCTCGTCTG   540

R  K  D  L  M  V  R  D  G  A  V  H  A  E  D  K  N  G  D  A    200
CGGAAGGACCTCATGGTACGCGATGGGGCCGTTCATGCTGAAGACAAAAACGGTGACGCC   600

S  C  S  A  D  T  S  N  P  R  E  S  I  P  E  S  D  A  V  A    220
TCGTGCTCAGCAGACACTTCGAATCCACGAGAGTCGATTCCGGAAAGTGACGCAGTCGCC   660

P  E  N  Q  E  N  R  Q  E  E  A  P  V  C  S  C  P  W  R  A    240
CCGGAGAATCAGGAGAACCGTCAAGAAGAGGCTCCAGTTTGCTCGTGTCCGTGGAGAGCT   720

P  K  K  H  E  A  L  P  L  P  A  F  P  R  L  R  F  L  A  V    260
CCTAAAAAGCACGAAGCTCTGCCGCTTCCCGCCTTTCCGCGACTCCGTTTTCTGGCAGTG   780

R  N  R  G  G  W  S  V  R  P  P  L  P  C  A  F  T  Y  L  F    280
CGCAACCGCGGCGGGTGGTCTGTCCGTCCACCTCTGCCGTGTGCCTTTACTTACCTATTC   840

L  G  D  L  Q  T  G  D  S  S  R  E  S  C  W  A  S  S  E  S    300
CTCGGGGATTTGCAGACCGGCGATTCTTCTCGTGAGTCCTGTTGGGCCTCCTCGGAGTCC   900

T  T  P  S  E  A  F  P  V  L  K  D  I  R  F  L  E  F  T  T    320
ACAACGCCTTCTGAAGCGTTTCCTGTCTTGAAAGACATCCGATTTCTCGAGTTCACCACC   960

I  G  L  D  L  F  R  Q  F  L  V  K  H  N  V  T  S  A  E  H    340
ATAGGTTTGGATTTGTTCAGGCAGTTTCTAGTCAAACACAACGTGACGTCTGCGGAGCAC  1020

I  A  I  G  M  W  H  M  S  P  L  Q  D  F  L  L  F  L  V  R    360
ATTGCGATAGGCATGTGGCACATGAGTCCGTTGCAGGACTTCTTGTTGTTTTTGGTGCGC  1080

E  N  S  K  P  K  R  R  F  H  K  L  R  S  L  D  V  H  G  A    380

GAGAATTCGAAGCCAAAACGGCGATTCCACAAGCTGAGGAGTCTAGACGTGCACGGAGCG  1140

R  F  I  W  P  P  D  E  F  W  Y  F  F  W  R  S  W  C  D  F    400
AGATTCATATGGCCACCAGACGAGTTCTGGTATTTCTTTTGGCGAAGTTGGTGTGACTTC  1200
```

```
T  Q  I  W  K  E  S  F  L  Q  R  S  S  D  S  D  R  G  D  A    420
ACGCAAATCTGGAAGGAAAGCTTTCTTCAGCGCAGCTCAGACAGCGACCGGGGAGACGCG  1260

R  R  R  A  S  A  L  C  D  D  H  W  T  S  C  P  K  D  W  S    440
CGACGACGAGCTTCTGCTCTGTGTGACGACCACTGGACGTCTTGCCCCAAAGATTGGTCT  1320

T  C  P  D  Q  W  G  T  D  N  D  P  L  L  R  V  S  D  G  D    460
ACGTGCCCTGACCAATGGGGAACAGACAACGACCCGCTACTACGAGTTAGCGACGGTGAC  1380

C  E  P  Q  D  E  D  L  V  E  E  G  G  S  I  N  D  E  F  L    480
TGCGAACCACAAGACGAAGATTTGGTGGAAGAAGGTGGCTCAATCAATGACGAATTCCTG  1440

S  D  A  G  E  D  E  G  K  D  R  E  S  I  V  R  M  L  K  R    500
TCGGATGCGGGAGAGGATGAGGGGAAAGACCGCGAGAGTATCGTGCGCATGCTGAAAAGG  1500

L  P  C  L  R  R  V  L  L  S  G  F  V  G  T  F  V  A  E  E    520
CTTCCCTGCCTTCGCCGTGTTCTTTTGTCAGGTTTTGTGGGCACATTTGTAGCCGAGGAA  1560

A  D  E  A  I  R  F  F  E  D  I  F  P  Q  A  V  V  D  V  E    540
GCAGACGAGGCCATTCGCTTTTTCGAGGATATATTCCCCCAAGCAGTTGTGGACGTGGAG  1620

G  E  I  I  V  S  T  N  A  L  L  E  A  V  V  Q  A  T  A  H    560
GGCGAGATCATCGTCAGCACGAATGCTCTCCTGGAGGCCGTTGTCCAAGCGACTGCGCAT  1680

P  G  W  A  S  R  L  D  S  F  M  A  R  K  R  E  H  R  A  Q    580
CCTGGGTGGGCGTCCCGACTTGATTCGTTTATGGCTCGAAAAAGGGAGCACCGCGCGCAA  1740

V  T  Q  A  T  G  S  R  A  S  P  W  T  E  D  F  D  R  S  G    600
GTGACTCAGGCGACAGGGTCACGAGCAAGCCCATGGACAGAAGATTTTGATCGAAGTGGA  1800

V  T  A  A  A  V  G  A  P  N  S  E  V  S  T  S  N  A  E  L    620
GTGACTGCGGCCGCCGTGGGAGCACCAAACAGTGAAGTCTCAACCTCTAACGCCGAGCTA  1860

E  R  D  D  E  S  E  H  L  A  L  E  G  R  R  Y  D  E  R  N    640
GAACGGGATGACGAGAGTGAGCATCTTGCACTCGAAGGTCGTCGGTACGATGAGCGGAAC  1920

W  E  A  L  D  G  V  F  L  S  E  R  G  K  E  N  F  G  R  W    660
TGGGAAGCGTTAGATGGAGTCTTCCTGTCTGAACGAGGAAAAGAGAATTTCGGTCGGTGG  1980

L  A  L  G  V  I  E  D  V  Y  I  E  V  S  N  P  T  Y  P  K    680
CTGGCTCTGGGCGTCATCGAGGACGTTTACATAGAAGTGAGTAATCCAACATATCCAAAA  2040

V  H  L  Q  I  N  A  F  H  R  Y  C  V  S  T  Y  F  K  C  L    700
GTGCATTTGCAAATCAATGCATTTCATCGATATTGCGTGTCTACATATTTTAAATGTTTG  2100

E  L  P  A  T  K  M  E  D  P  V  G  Q  D  W  G  L  P  S  V    720
GAACTGCCAGCAACCAAGATGGAGGATCCCGTAGGGCAAGACTGGGGTCTTCCATCAGTG  2160

T  V  R  H  V  V  Q  W  G  T  K  Y  V  Q  D  F  L  D  I  V    740
ACTGTCCGTCACGTGGTGCAGTGGGGTACGAAGTATGTTCAGGATTTTTTAGATATTGTT  2220

S  T  F  R  H  V  I  L  N  L  N  Y  I  F  W  T  D  R  G  E    760
TCCACCTTCCGGCACGTCATTCTCAATTTGAATTACATCTTCTGGACAGACCGGGGAGAA  2280

Y  V  D  A  N  A  E  R  C  L  G  S  E  A  G  E  A  C  R  T    780
TATGTAGACGCAAACGCTGAGAGATGCCTCGGATCTGAAGCGGGCGAAGCATGTAGGACA  2340

P  S  E  G  N  L  I  Q  S  D  A  P  C  A  R  T  D  G  R  A    800
CCGTCAGAGGGAAATCTTATACAGAGCGACGCGCCATGCGCGCGCACAGACGGTCGGGCT  2400

Q  D  V  H  D  V  A  K  A  D  F  S  V  D  Q  E  S  G  E  T    820
CAGGATGTCCATGACGTGGCAAAAGCAGATTTCTCCGTAGATCAGGAGTCTGGTGAGACG  2460

D  A  E  P  P  P  V  V  S  A  D  T  R  F  L  Q  C  S  E  S    840
GACGCGGAGCCCCCGCCGGTTGTATCGGCTGACACGCGCTTTCTCCAGTGCTCCGAAAGT  2520

A  S  P  A  D  G  A  S  I  R  S  S  D  A  G  D  E  R  L  V    860
GCCAGCCCTGCCGACGGGGCCAGTATCAGAAGCAGCGACGCCGGTGACGAACGGTTGGTG  2580
```

```
A  S  D  R  T  A  H  Q  G  S  K  E  V  V  R  L  G  E  F  D    880
GCTTCGGATAGAACGGCTCACCAAGGGAGCAAGGAGGTTGTTCGTTTGGGTGAATTTGAC  2640
                   5’-aagggagcaaggaggttgttcgt                        259880 scrn fw

G  H  F  P  N  V  V  G  L  L  L  P  L  Y  W  M  D  D  A  T    900
GGTCATTTCCCGAACGTTGTTGGGCTTCTCCTGCCCCTGTACTGGATGGATGATGCAACA  2700

W  S  T  D  E  I  A  R  I  A  E  K  Y  R  S  Q  T  Q  V  V    920
TGGTCCACTGATGAGATAGCGCGAATTGCGGAGAAGTACAGAAGCCAAACGCAGGTCGTA  2760

R  I  K  N  L  F  P  I  M  G  E  Y  A  T  V  D  D  P  V  S    940
CGTATCAAAAACCTTTTTCCAATCATGGGGGAATACGCCACGGTAGACGATCCAGTGTCA  2820

L  M  A  R  D  F  E  I  V  T  R  I  C  K  D  T  M  R  V  I    960
CTGATGGCTCGAGATTTCGAAATTGTGACACGCATCTGTAAAGACACAATGCGTGTAATT  2880

D  Y  R  V  Y  S  F  F  S  W  K  D  E  L  G  L  D  E  N  T    980
GATTATCGAGTTTACAGCTTTTTCTCGTGGAAGGATGAGCTCGGCCTCGATGAGAACACG  2940

L  I  P  Q  F  L  K  E  F  V  S  N  N  P  E  F  T  L  A  K   1000
TTGATACCTCAGTTCTTGAAGGAGTTTGTATCAAACAATCCTGAATTTACTTTGGCTAAG  3000

R  L  D  S  P  S  M  T  Q  T  G  V  V  T  V  G  Q  E  A  L   1020
CGCCTCGACTCTCCTTCGATGACGCAGACAGGTGTGGTGACTGTCGGACAAGAAGCGCTT  3060
                                                      5’-ctt       Fw homology arm

C  Q  L  R  V  Y  I  W  I  K  R  D  V  L  *                   1034
TGTCAATTGCGCGTGTACATATGGATTAAACGAGATGTGCTGTGAacgtcaaacatgcaa  3102
tgtcaattgcgcgtgtacatatggattaaacgagatgtgctg                         Fw homology arm
                                           3’tgcagtttgtacgtt       Rv homology arm

tgtgagcaaacggcaaagatgttcacgaactaaccttttcataaattattgagcggtcca
acactcgtttggcgtttctacaagtgcttg                                     Rv homology arm

cgaagccccatgcattcgttgtttgtactgcagacat
```

Figure S7. **HA$_3$-epitope tagging of FBXO14**, as described in Fig. S6. The positive result for PCR2 in the edited strains is consistent with resistance of the strains to growth in the presence of chloramphenicol. The positive result for PCR1 indicates that the remainder of the locus is intact.

A. FBXO14 gene editing strategy

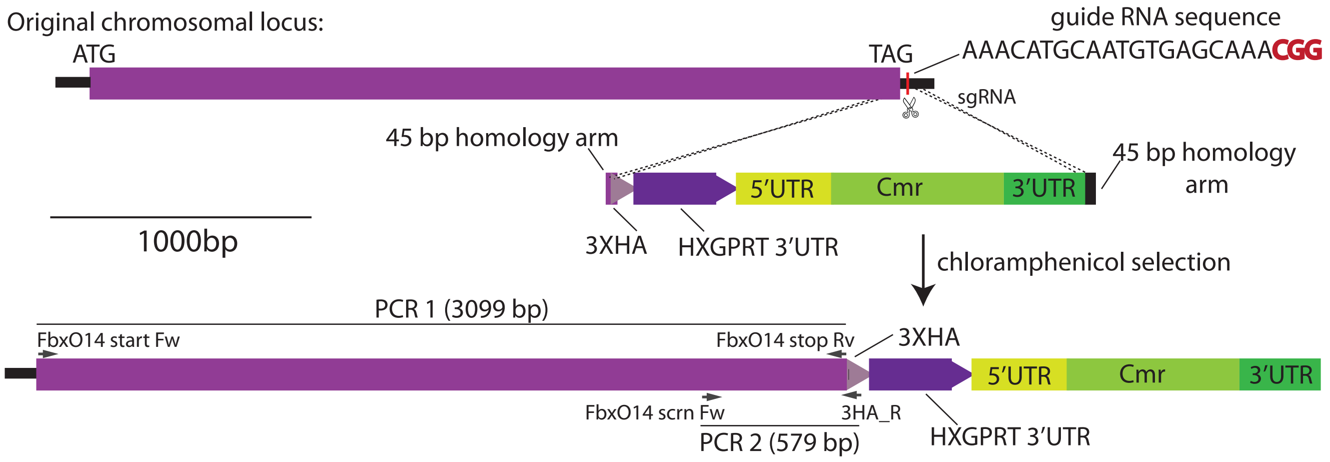

B. PCR assessment

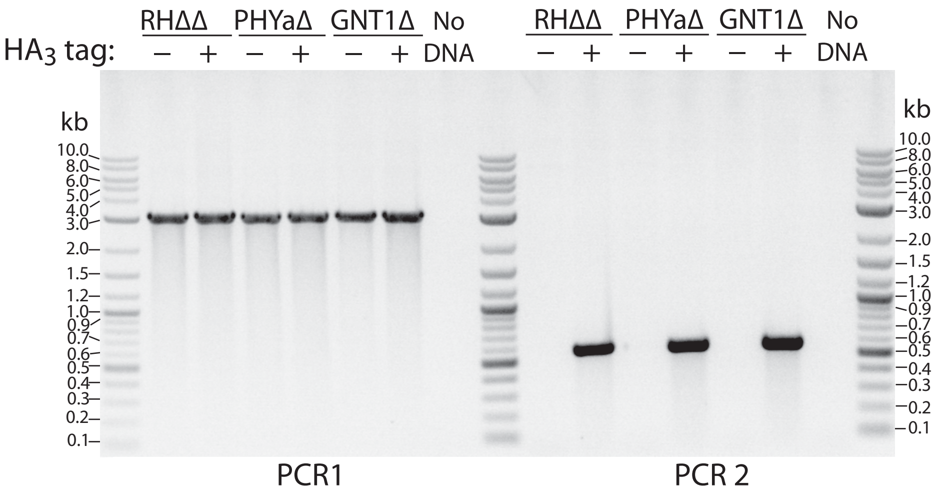

Fig. S8. DNA and protein master sequence of *Toxoplasma gondii* FBXO1 (TGME49_310930 and TGGT1_310930). Strain GT1 nt sequences are above ME49 sequences. Differences between strains are colored in red. Amino acid sequence motifs are colored distinctly. Yellow highlighting indicates peptides found in FBXO1 from the GT1 strain.

```
tggtagactgcatccgcgggacacgctgtgcaggctcagctttgcgtaaaggacttaccc
agcacatttaacgttcactaccacggaaaaaacttttctttccgtgctcaactgcgcctt
ctcgctcggggtcccggaactgcgtgcttttttgcccagctccctcttcgtgtcccca
ca

ATGGGCAACACGGAATCCACGGCCGCTGAGTTGCACGACAACTACTTGGCGGTTCTTAAA   60 - GT1
ATGGGCAACACGGAATCCACGGCCGCTGAGTTGCACGACAACTACTTGGCGGTTCTTAAA   60 - Me49
M  G  N  T  E  S  T  A  A  E  L  H  D  N  Y  L  A  V  L  K     20 N-myristoylation
motif

AGCGTCGAGGGTCGGTTGGGACAGTACCCAAGCCGAACGCTCTCCCCTCGGTCGCAGTCA
AGCGTCGAGGGTCGGTTGGGACAGTACCCAAGCCGAACGCTCTCCCCTCGGTCGCAGTCA  120
S  V  E  G  R  L  G  Q  Y  P  S  R  T  L  S  P  R  S  Q  S     40

AGCGGTGTGGATCGGTCTCGCGCGAGGGTACGCCCCGAGGAAGCGTACCGGGATCCTGGA
AGCGGTGTGGATCGGTCTCGCGCGAGGGTACGCCCCGAGGAGGCGTACCGGGATCCTGGA  180
S  G  V  D  R  S  R  A  R  V  R  P  E  E  A  Y  R  D  P  G     60

AAGCGGGGTGCTGGAAACCGCCCTCGCAACTTGCCATCCAAAGGCGAGAAGCCCTCTC
GG
AAACGGGGTGCTGGAAACCGCCCTCGCAACTTGCCATCCAAAGGCGAGAAGCCCTCTCGG  240
K  R  G  A  G  N  R  P  R  N  L  P  S  K  G  E  K  P  S  R     80

TCTGCGTACAAGTCGGGATCCACTTCCTCAGTGGACCAGTTCAACAACGGGCCTGGTGCG
TCTGCGCACAAGTCGGGATCCACTTCCTCAGTGGACCAGTTCAACAACGGGCCTGGTGCG  300
S  A  YH K  S  G  S  T  S  S  V  D  Q  F  N  N  G  P  G  A    100

AACCGGAAACGCGGGAAGGACAGCGTCCAGCGGCGCGATGATGCCCCGCCACAAGCAGAT
AACCGGAAACACGGAAAGGACAGCGTCCAGCGGCGCGATGATGCCCCGCCACAAGCAGAT  360
N  R  K  RH G  K  D  S  V  Q  R  R  D  D  A  P  P  Q  A  D    120

CAGGAACACTCTTCGCTTCATCCAGCTGACAGCAAGCGCCGGCCTGCCGCGAAAGGAGAG
CAGGAACACTCTTCGCTTCAACCAGCTGACGGCAAGCGCCGGCCTGCCGCGAAAGGAGAG  420
Q  E  H  S  S  L  HQ P  A  D  SG K  R  R  P  A  A  K  G  E    140

ACTCCCAATGTGGAGTCGACTGCGACAGGATCCGACTGGGGAGACTCCGCTGTCAACG
AT
ACTCCCAATGTGGAGTCGACTGCGACAGGATCCGACTGGGGAGACTCCGCTGTCAACGAT  480
T  P  N  V  E  S  T  A  T  G  S  D  W  G  D  S  A  V  N  D    160

TTTGCTGAAGAGGATAGCTGCGCCGTTGAGAGTCCAGCACACTTTAAGCTTCAGACGAAG
TTTGCTGAAGAGGATAGCTGCGCCGTTGAGAGTCCAGCACACTTTAAGCTTCAGACGAAG  540
F  A  E  E  D  S  C  A  V  E  S  P  A  H  F  K  L  Q  T  K    180

GAGTCGGGTGTCAAGGCACCTTCGCCGCGCCTCTTCCCCTGTGAAACGCTGTATGAGGTC
GAGTCGGGTGTCAAGGCACCTTCGCCGCGCCTCTTCCCCTGTGAAACGCTGTATGAGGTC  600
E  S  G  V  K  A  P  S  P  R  L  F  P  C  E  T  L  Y  E  V    200

ACTTCGCAGATAGACCTCGCGGATGAGAAAACGCAAGTCGAAGACGGAGAGCTCAGCTCT
ACTTCGCAGATAGACCTCGCGGATGAGAAAACGCAAGTCGAAGACGGAGAGCTCAGCTCT  660
T  S  Q  I  D  L  A  D  E  K  T  Q  V  E  D  G  E  L  S  S    220

CTCCCGAGTTCCCCCACGGCCTCTGCAGAGGTCGCGACGGACAAGCCCGGGGAGGGAC
GA
CTCCCGAGTTCCCCCACGGCCTCTGCAGAGGTCGCGACGGACAAGCCCGGGGAGGGACGG  720
L  P  S  S  P  T  A  S  A  E  V  A  T  D  K  P  G  E  G  R    240
```

```
ACGGCGACGCGGGAAGACACGCGGACTGGTCGGTCAGCTGAGGGAGACAGAGGGCCTTGC
ACGGCGACGCGGGAAGACACGCGGACTGGTCGGTCAGCTGAGGGAGACAGAGGGCCTTGC  780
T  A  T  R  E  D  T  R  T  G  R  S  A  E  G  D  R  G  P  C    260

CATACAGAGAATCAGGTCGACGGAGCAGACGCGAAGGAGAATGAGTCAGACAACTCACAC
CATACAGAGAATCAGGTCGACGGAGCAGACGCGAAGGAGAATGAGTCAGACAACTCACAC  840
H  T  E  N  Q  V  D  G  A  D  A  K  E  N  E  S  D  N  S  H    280

GGCGAGTCAGACCAAGACCTGCGACGTCGGAGCGACGGCCGCGGTGCATCCTCCCAGAGA
GGCGAGTCAGACCAAGACCTGCGACGTCGGAGCGACGGCCGCGGTGCATCCTCCCAGAGA  900
G  E  S  D  Q  D  L  R  R  R  S  D  G  R  G  A  S  S  Q  R    300

TCCAGGGACGAGGCCTCGAACAGTGGCGAGCCTCCGTCACAATCGAAACCGTCGCAAC
TT
TCCAGGGACGAGGCCTCGAACAGTGGCGAGCCTCCGTCACAGTCGAAACCGTCGCAGCTT  960
S  R  D  E  A  S  N  S  G  E  P  P  S  Q  S  K  P  S  Q  L    320

TCAAAGCAAATACGGAAGAAAGAACTCGCCCCGGAGCCTCTCCGCGCAGCAGATATCGAC
TCAAAGCAAATACGGAAGAAAGAACTCGCCCCGGAGCCTCTCCGCGCAGCAGATATCGAC 1020
S  K  Q  I  R  K  K  E  L  A  P  E  P  L  R  A  A  D  I  D    340

CTTTCCTCAGATGTCCGTGTCGGAGCCTCGTTGAAGGGGGGGAAGGGAGGGAAGGGAGAC
CTTTCCTCAGATGTCCGTGTCGGAGCCTCGTTGAAGGGGGGGAAGGGAGGGAAGGGAGAC 1080
L  S  S  D  V  R  V  G  A  S  L  K  G  G  K  G  G  K  G  D    360

GCTGAGGGTGATAATGTCGCCGACCTCACAGCTGGATTCGAGGGATTTTCGCCGTACTCG
GCTGAGGGTGATAATGTCGCCGACCTCACAGCTGGATTCGAGGGATTTTCGCCGTACTCG 1140
A  E  G  D  N  V  A  D  L  T  A  G  F  E  G  F  S  P  Y  S    380

CCCTCCTACTCTATTGGCTTCAGTCAGCCGTCGTCGAAGAAGGCTGCAGAAACGACAG
CT
CCCTCCTACTCTCTTGGCTTCAGTCAGCCGTCGTCGAAGAAGGCTGCAAAAACGACAGCT 1200
P  S  Y  S  IL  G  F  S  Q  P  S  S  K  K  A  A  EK  T  T  A    400

GAAAACTCCAAAGCACACCTACCAGGAGGAACCTGCGGCAGCGGCAACAACACTGCTGTC
GAAAACTCCAAAGCACACCTACCAGGAGGAACCCGCGGCAGCGGCAACAACACTGCTGTC 1260
E  N  S  K  A  H  L  P  G  G  T  CR  G  S  G  N  N  T  A  V    420

GACCGGGACGCGTGCATCTACGGAAACAAGAAAAACCACTTTCCCTTCCTCGACGAACCT
GACCGGGACGCGTGCATCTACGGAAACAAGAAAAACCACTTTCCCTTCCTCGACGAACCT 1320
D  R  D  A  C  I  Y  G  N  K  K  N  H  F  P  F  L  D  E  P    440   predicted F-box

GCCTTGTCACTGCTTGTACCATTTCTCTTCGGTAGAAGCCTCGCGACTTGCATGACCGTC
GCCTTGTCACTGCTTGTACCATTTCTCTTCGGCAGAAGCCTCGCGACTTGCATGACCGTC 1380
A  L  S  L  L  V  P  F  L  F  G  R  S  L  A  T  C  M  T  V    460

TGTCCACACTGGTTCATGAAAATCAATCGgtacgcgacaagacgccactgcggtgaac
at
TGTCCACACTGGTTCATGAAAATCAATCGgtacgcgacaagacgccactgcggtgaacat 1440
C  P  H  W  F  M  K  I  N  R                                  470

agactcctacctcacatacaaagggattctaagatcttggcagtgtgatctctactaaga 1500
agactcctacctcacatacaaagggattctaagatcttggcagtgtgatctctactaaga 1500

gcctggtcgttccctttggtccacgcatctcatttttctactcgaatcagcagtggtcct
gcctggtcgttccctttggtccacgcatctcatttttctactcgaatcagcagtggtcct 1560

tgaaaggggggtttccatccagcgtttttcctcactgtccgctgtttttttgcctgcctgac
tgaaaggggggtttccatccagcgtgtttcctcactgtccgctgtttttttgcctgcctgac 1620
```

```
gctttctctttttcagGGCTATGGAACGCATGTGTGGGCCTGCCACAAAAGGTTTCCA
GC
gctttctctttttcagGGCTATGGAACGCATGTGTGGGCCTGCCACAAAAGGTTTCCAGC 1680
                 A  M  E  R  M  C  G  P  A  T  K  G  F  Q  Q  484

AAATGTACTCAAAATACTTGGAAGTCTGGGGAAGCGCTGTCAAACTTCAGCCTCTCCAGA
AAATGTACTCAAAATACTTGGAAGTCTGGGGAAGCGCTGTCAAACTTCAGCCTCTCCAGA 1740
  M  Y  S  K  Y  L  E  V  W  G  S  A  V  K  L  Q  P  L  Q  T  504

CAGTTGGCGATGGCGGAGTCCGCGTGGACTGGGTGATTTTTGCCAAGgtgcgtctcggtg
CAGTTGGCGATGGCGGAGTCCGCGTGGACTGGGTGATTTTTGCCAAGgtgcgtctcggtg 1800
  V  G  D  G  G  V  R  V  D  W  V  I  F  A  K                 520

ctttcctgtttgctgcgccttgccgtcgtggctgataaagggggcagtacacttgatga
ctttcctgtttgctgcgccttgccgtcgtggctgataaaggggacagtacacttgatga 1860

tatgcatgagatagccttccatacttctgcgttgattgtgccagacgttcctgttcgc
tc
tatgcatgagatagccttccatacttctgcgttgattctgccagacgttcctgttcgctc 1920

tttgcgtcaggagttccgcttcataataaaagcgttccgcttccagttggttttcttctc
tttgcgtcagcagttccgcttcataataaaagcgttccgcttccagttggttttcttctc 1980

ttcggtagctggcccttcggtttctttgcttggccagagtgaagaaccggggccggcgta
ttcggtagctggaccttcggtttctttgcttggccagagtgaagaaccggggccggcgta 2040

cagggatgagcttccgcgaatgcaccgcaccgggcaaaatgcgtcaagaatactctgtgg
cagggatgagcttccgcgaacgcatcgcaccgggcaaaatgcgtcaagaatactccgtgg 2100

tatgtttttagGTCCTCCCGGAATGTGAAGGTCACATCCTTGACATATCGTATACCTA
TT
tatgtttttagGTCCTCCCGGAATGTGAAGGTCACATCCTTGACATATCGTATACCTATT 2160
             V  L  P  E  C  E  G  H  I  L  D  I  S  Y  T  Y  S  536

CCTACATTTCCGAAAGTTCGAGTTCGACTGTTCGTCCGGTGGACGAAGCGGACAAGCCCG
CCTACATTTCCGAAAGTTCGAGTTCGACTGCTCGTCCGGTGGACGAAGCGGACAAGCCCG 2220
  Y  I  S  E  S  S  S  S  T  VA  R  P  V  D  E  A  D  K  P  A  556

CGGCGGGTGGGAAGAGAGATCGCGGACGGCAAGGAGAAAAAAGGCGATCCCTTTCTGCCA
CGGCGGGTGGGAAGAGAGATCGCGGACGGCAAGGAGAAAAAAGGCGATCCCTTTCTGCCA 2280
  A  G  G  K  R  D  R  G  R  Q  G  E  K  R  R  S  L  S  A     576

ACTTCGACGAAGGCGTCATTCTCTCTCCGTGCGGACAGgttcgtggactggaaagcagtg
ACTTCGACGAAGGCGTCATTCTCTCTCCGTGCGGACAGgttcgtggactggaaagcagcg 2340
  F  D  E  G  V  I  L  S  P  C  G  Q                          589

cgtctctgagagctcgtttcttcttcagtttttgcagctccttgcgcttccaccaccc
at
cgtctctgagagctcgtttcttcttcagtttttgcagctccttgcgcttccaccacccat 2400

gcgagagcaccggcgcgtctctcagtcgatgaggggactggaccgagaaacctacactcg
gcgagagcactggcgcgcctctcagtcgatgaggggactggaccgagaaacctacactcg 2460

cctgaagggtgcggatccagtggctctcatgttagcatggtttcgagaacagtcggtata
cctgaagggtgcggatccagtggctctcatgttagcatggtttcgagaacagtcggtata 2520

tccggccaccgcggacaaaccgtgtgcctatcgcctgcatcgcctttggtctcctaactc
tctggccaccgcggacaaaccgtgtgcctatcgcctgcatcgcctttcgtctcctaactc 2580

cggtgtttctgtggatgtttctctgcagCCTTGCCCGACCAACATGCCTTCGCGCGTG
TT
cggtgtttctgtggatgtttctctgcagCCTTGCCCGACCAACATGCCTTCGCGCGTGTT 2640
```

```
                            P  C  P  T  N  M  P  S  R  V  F   600

CACAGTGTCTTACTCGCTGGCGGCGGGTGCGGCGAAGAGCAGCCGAACGCTTTGGATGCA
CACGGTGTCTTACTCGCTGGCGGCGGGTGCGGCGAAGAGCAGCCGAACGCTTTGGATGCA 2700
 T  V  S  Y  S  L  A  A  G  A  A  K  S  S  R  T  L  W  M  H   620

CCGAGACTTGTGCCGGTTCCACGGAGACGAGACTGGGGTCGCTGCCATGGGCTCCGTCTC
CCGAGACTTGTGCCGGTTCCACGGAGACGAGACTGGGGTCGCTGCCATGGGCTCCGTCTC 2760
 R  D  L  C  R  F  H  G  D  E  T  G  V  A  A  M  G  S  V  S   640

AAGTGTCTGCGTAGGAGACTTCGTGGAAGTCGCTGTCACTGTGTACAACGGAGGCGGGCG
AAGTGTCTGCGTAGGAGACTTCGTGGAAGTCGCTGTCACTGTGTACAACGGAGGCGGGCG 2820
 S  V  C  V  G  D  F  V  E  V  A  V  T  V  Y  N  G  G  G  R   660

CGTTGCTCTGGACAAAGTCAAGTGGCTGCCTGCGCGAGTCGAGTGGAGACGGGAGGCT
GT
CGTTGCTCTGGACAAAGTCAAGTGGCTGCCTGCGCGAGTCGAGTGGAGACGGGAGGCTGT 2880
 V  A  L  D  K  V  K  W  L  P  A  R  V  E  W  R  R  E  A  V   680

TTCGACTCGCGGCGTCTTCAACAGAGAAATCTGTCCTCTCGAACGCTGCTCTCCCGACTG
TTCGACTCGCGGCGTCTTCAACAGAGAAATCTGTCCTCTCGAACGCTGCTCTCCCGACTG 2940
 S  T  R  G  V  F  N  R  E  I  C  P  L  E  R  C  S  P  D  W   700

GCTTCCTGCAGACCAGTTCCGCATCATGACTACGGAACGCCTGAAGGCTCCAGgtgagtc
GCTTCCTGCAGACCAGTTCCGCATCATGACTACGGAACGCCTGAAGGCTCCAGgtgagac 3000
 L  P  A  D  Q  F  R  I  M  T  T  E  R  L  K  A  P  E         718

tgtcggggactggacgggccgacgctctcctgaacgtgacttggattcgaagggtcagac
tgtcggggactggacgggccgacgctctcctgaacgtgactcggattcgaagggtcagac 3060

agccagtcctttctgcctgcggtccacctccgtatcactgaacactgtgcaacagggg
at
agccagtcctttctgcctgcggtccaccaccgtatcactgaacactgtgcaacaagggat 3120

cgaacggcagctcgtatctgcctggtgcagtgcacagagatgatgacacgcgggaccgtg
cgaactgcagctcgaatctgcctggtacagtgcacagagatgatgacacgcgggaccgtg 3180

gtgctgatcatcatgatcgtcctagcatctgaggagctgttttctctcgctgttaatacg
gtgctgatcatcatgatcatcctagcatctgaggagctgttttctctcgctgttaatacg 3240

catgacaccagaaatccatgaaagcgtacgcgtgaaacgcaagcgatttttggaatactt
cgtgacaccagaaatccatgaaaccgtacgcgtgaaacgtaagcgatttttggaatactt 3300

tcaatgtcgtaagacatggaactccagcgcgtttcgctctcgtggaaagaaaacagct
gg
tcagtgtcgtaagacatggaactccagcgcgtttcgctctcgtggaaagaaaacagctgg 3360

aagcgaggaagcaaagccgtgggcgaagggccaaaaagtcatgacggggaaa-tgcgggg
aagcgaggaagcaaagccgtgggcgaagggccaaaaagtcatgccgagggaaatgcgggg 3420

atcgaaaggacggattccaaataggaacctgcgcactgtgcagtcggcctttcgcggcaa
atcgaaaggacggattccaaataggaacctgcgcactgtgcagtcggcctttcgcggcaa 3480

ccctgctggaaccactgcagtgcatgaagaagatatgaaggactcctgtagcacccgtag
ccctgctggaaccactgcagtgcatgaagaagatatgaaggactcctgtagcacccgtag 3540

ctgaaagaacatttctttagctgaagcaagtcccgaaagatgtccgattaactgtgca
gA
ctgaaagaacatttctttagctgaagcaagtcccgaaagatgtccgattaactgtgcagA 3600

GGACTTCTCGCCGTGTTTGAAACATGTCAAGACAGAATTCTCCGGAATGGATGTCGCCGT
GGACTTCTCGCCGTGTTTGAAACATGTCAAGACAGAATTTTCCGGAATGGATGTCGCCGT 3660
```

```
 D  F  S  P  C  L  K  H  V  K  T  E  F  S  G  M  D  V  A  V   738

TCGCAAATCAACATATCGAGCAGTCCGCCAAGgtaggctgaaaccgccttcgataggaca
TCGCAAATCAACATATCGAGCAGTCCGCCAAGgtaggctgaaaccgccttcgataggaca 3720
 R  K  S  T  Y  R  A  V  R  Q  G                              748

tcgcacgca----------tatatatatatatgtatgtatatgctggaaatatgtgttaa
tcgcacgcatatatatatatatatatatatatgtatgtgtatgttggaaatatgtgttaa 3780

tacttctacattggattaaaacactggagttacgctataactttagcgtgattgtctcga
tacttctacattggattaaaacactggagttacgctataactttagcgtgattgtctcca 3840

gcatcgatggatagggcaggtgcatgggaagtctgtggacagacggcacatatacaccga
gcatcgatggatagggcaggtgcatgggaagtctgtggacagacggcacatatacaccga 3900

acgcgcgtaggacgttggtgagcgccagttgcttgtcgttgcctacaccggaggggaatg
acgcgcgtaggacgttggtgagcgccagttgcttgtcgttgcctacaccggaggggaatg 3960

tgcattttacgcaaacgaagtctatttttcggcagaacacaggcgtttcacatctgtttt
tgaattttacgcaaacgaagtctatttttcggcagaacacaggcgtttcacatctgtttt 4020

cttccggatcgaatccttgcgacagtctgacatgcttgcctgggactaccacaactgtct
cttccggatcgaatccttgcgacagtctgacatgcttgcctgggactaccaaagctgtct 4080

gtcctcgctgcgcatgccacagGGTCGCTGGGATCAGCAGCGTGTCGGTCTTGGGGGTTC
gtcctcgctgcgcatgccacagGGTCGCTGGGATCAGCAGCGTGCCGGTCTTGGGGGTTC 4140
                        S  L  G  S  A  A  C  R  S  W  G  F     761

CCTTGTGAGATCCTTCCTCAAGGCGTCCCTGTTGTTTGCTCCCTGACGCGCTGGGGACTC
CCTTGTGAGATCCTTCCTCAAGGCGTCCCTGTTGTTTGCTCCCTGACGCGCTGGGGACTC 4200
P  C  E  I  L  P  Q  G  V  P  V  V  C  S  L  T  R  W  G  L     781

CAGCATGATCGATTCCTCTCCGTTCAGCTGCGCGAGgtgagtttcgccgattgctctcgc
CAGCATGATCGATTCCTCTCCGTTCAGCTGCGCGAGgtgagtttcgccgattgctctcgc 4260
Q  H  D  R  F  L  S  V  Q  L  R  E                             793

gaattgccgaaaatgtcggcccgtcgaagcaaaaagagctgccgattctcagggtttccg
gaattgccgaaaatgtcggcccgtcgaagccaaaagagctgccgattctcagggtttccg 4320

ttttgcgagagtgcgagcgagtacgagcgactttctgcggcaccgcagtggctttgagcg
ttttgcgagagtgctagcgagtacgagcgactttctgcggcaccgcagtggctttgagcg 4380

ttgacgtttttctaggtcgttgccggaacacagcagatgggtccagccttagacgatcgc
ttgaagtttttctaggtcgttgccggaacacagcagatgggtccagccttagacgatcgc 4440

ctgtctccacctttccggcatctgatgcccgtaaacgtatcaaattcatggcctgtctgg
ctgtctccacctttccggcatctgatgcccgtaaacgtatcaaattcatggcctgtctgg 4500

aatttgcttgtcgccgtagctacagagagagggtgtgtgggtgtgtgtgctgggaagatg
aatttgcttgtcgccgtagctacagagagagggtgtgtgggtgtgtgtgctgggaagatg 4560

acatgtctgaaacgacgtaagacggagaaacgcgacgtagttcaatttcggtgtagtgcc
acatgtctgaaacgacgtaagacggagaaacgcgacgtagttcaatttcggtgtagtgcc 4620

acaaacgctttactgtattcacgtcgagcggtcttctgcagagccactctgagagttaag
acaaacgctttactgtattcacgtcgagcggtcttctgcggagccactctgagagttaag 4680
                5'-tcacgtcgagcggtcttctg-3'                          Seq For

atatggttgaatctatatcctaagttctgtagtttggtcgcttcacccatgttcagtgtt
atatggttgaatctatatcctaagttctgtagtttggtcgcttcacccgtgttcagtgtt 4740

ttgtcttggtgctttgttttgcggtcggcctccagatggtaaagtgtagacaatcccaga
```

```
ttgtcttggtgctttgttttgcggtcggcctccagatggtaaagtgtagacaatcccaga 4800

tctttccgcactgagtgctccatgggtgaccatctgagggaagtcttgacttgcctgtac
tctttccgcactgagtgctccatgggtgaccatctgagggaagtcttgacttgcctgtac 4860
                                        5'-tcttgacttgcctgtac

cgaatgcggtacgaggactttccacgtattcgcgtatgcggcctggccgtggtttgttcg
cgaatgcggtacgaggactttccaagtattcgcgtatgcggcctggccgtggtgtgttcg 4920
cgaatg-3'                                                         O1F1

cagGGCGACATAATCGATTACTACATGAGTCAGGGCGGAGCCAATGCGTGAgggaggtct
cagGGCGACATAATCGATTACTACATGAGTCAGGGCGGAGCCAATGCGTGAgggaggtct 4980
   G  D  I  I  D  Y  Y  M  S  Q  G  G  A  N  A  *             808
5'-ggcgacataatcgattactacatgagtcagggcggagccaatgcgaaaattggaagtggaggacg O1 left arm

cagggaaccccgtggaatatcaacggcgcttttggggatcaatgctgtttcaggggcac
cagggaaccccgtggaatatcaacggcgcttttggggatcaatgctgtttcaggggcac 5040
 5'-gaacccccgtggaatatcaacgg-3'                                    gRNA219 (PAM:CGG)
gaagatacgtgaacgtcctacgttgccgcgaaaacccctagttacgacaaagtccccgtg      O1 right arm

cagctcttcgaaatctcaatagttctgcaccaaggtttcatggagccgagttccggcttt
cagctcttcgaaatctcaatagttctgcaccaaggtttcatggagccgagttccggcttt 5100
gtcgag-5'                                                         O1 right arm
                     3'-gacgtggttccaaagtacctcg-5'                 O1R4
```

B. Inserted HA_3-tag sequence, insert sequence in *italics*

```
AAAATTGGAAGTGGAGGACGGGAATTCCCTAGGTACCCGTACGACGTCCCGGACTACGCT   60
K  I  G  S  G  G  R  E  F  P  R  Y  P  Y  D  V  P  D  Y  A     20 Linker,1st HA
aaaattggaagtggaggacg-3' (insert homology only)                    O1 left arm

GGCTATCCCTATGATGTGCCCGATTATGCGGGCTCTTATCCTTACGATGTTCCAGATTAT  120
G  Y  P  Y  D  V  P  D  Y  A  G  S  Y  P  Y  D  V  P  D  Y     40  2nd HA, 3rd HA
                                                3'-ggtctaata      3×HA Rev

GCCTAAtcaccgttgtgctcacttctcaaatcgacaaaggaaacacacttcgtgcagcat  180
A  *                                                           41
cggattagtggc-5'                                                   3×HA Rev
```

Figure S9. HA$_3$-epitope tagging of FBXO1 (TGGT1_310930). *A*, CRISPR/Cas9 strategy for introducing a C-terminal HA$_3$-tag. *B*, clonal analysis of edited clones in RHΔΔ and PHYaΔ strains. The increased length of the PCR1 product in edited compared to parental strains confirms introduction of the cassette, and is consistent with the resistance of the strains to growth in the presence of pyrimethamine. *C*, Western blot analysis of expression of FBXO1-HA$_3$, SKP1, and SAG1 in the clones. n.s., non-specific bands. *D*, sequencing of selected region of PCR1 in clones. Clones H6 and A11, which lacked errors, were selected for further studies.

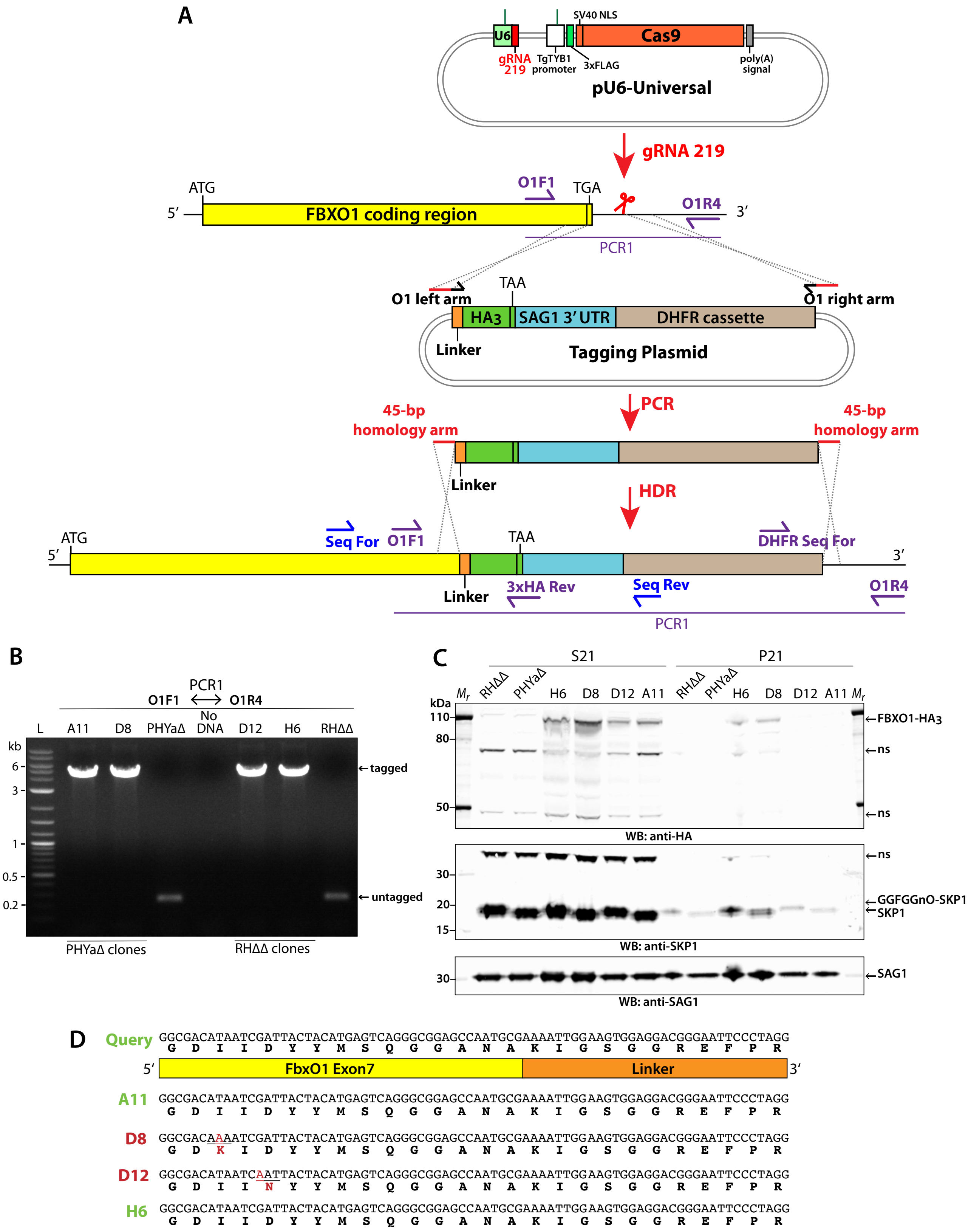

Figure S10. Localization of FBPs in parasites at low oxygen. Studies were performed on HFF infected parasites after 48 h at 0.5% O_2, and MeOH fixed. *A*, FBXO13-HA$_3$. *B*, FBXO14-HA$_3$. See Fig. 8 for details and comparative findings at 21% O_2. Scale bars = 1 μm.

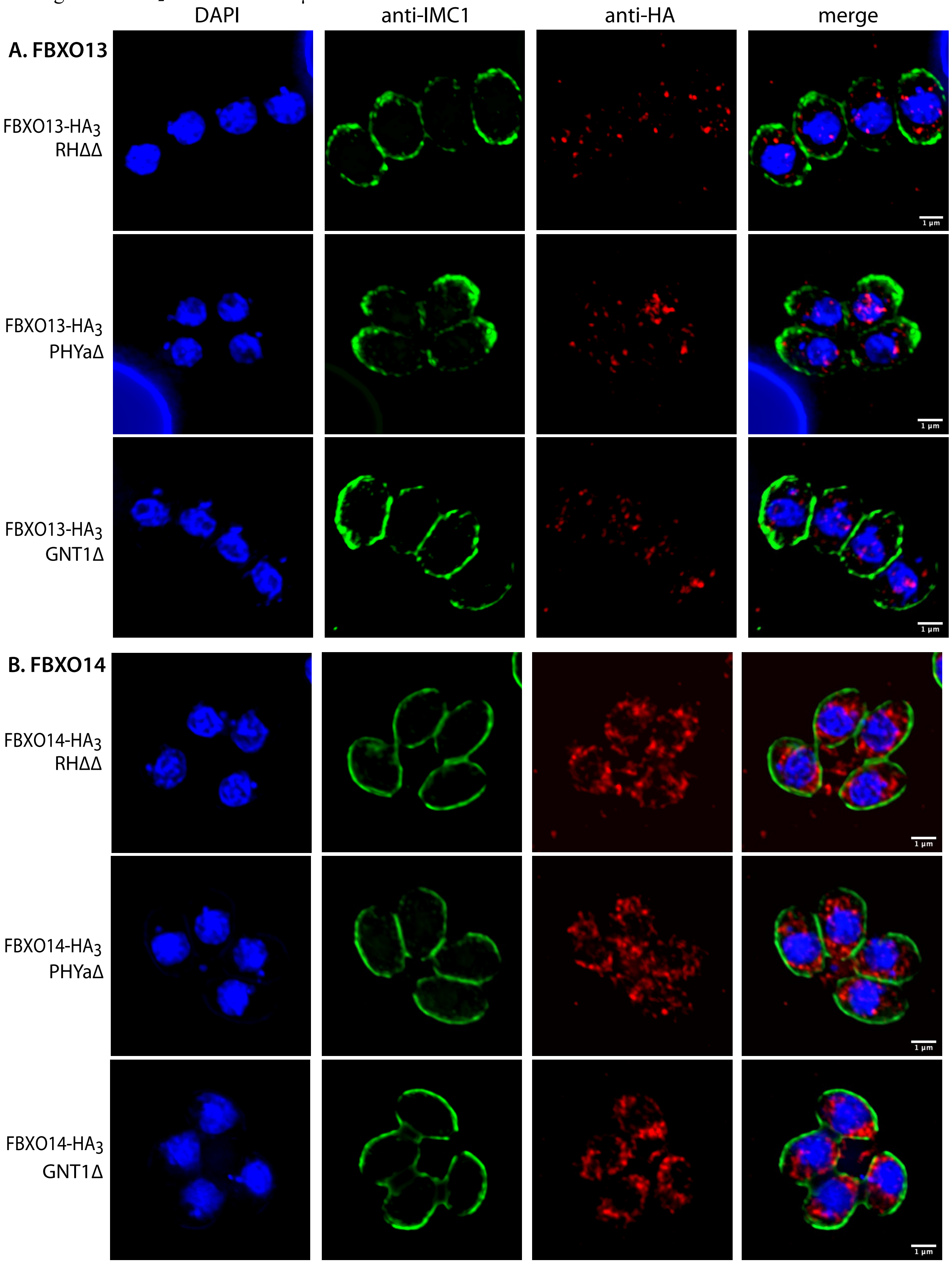

Figure S11. SKP1 heavy peptides. The location of the tryptic peptide$_{145\text{-}161}$ bearing the target Pro$_{154}$ is shown as red font in the context of the full protein sequence. *A*, HPLC analysis of purity, based on detection by absorbance at 214 nm, of the synthetic peptide containing two copies of $^{13}C_5$,^{15}N-Val. MALDI-TOF-MS (positive ion mode) analysis supports purity and identity based on exact mass of the singly charged protonated monoisotopic ion. *B*, similar analysis of the synthetic peptide containing *2S*,*4R*-hydroxyproline (Hyp) in place of Pro$_{154}$. C, similar anlaysis of the synthetic peptide containing αGlcNAc linked to Hyp$_{154}$.

```
MSKERMGDAR KVTLVSQEGD EFDVDIEVAS MSALIKTMVE EDSDCQESIP LPNVDTCILK KIIEYCEHHH
NNPPEEIPKP LKSSNLAEVV SEWDYQFINE NSDQKILFAL ILAANYLNIK PLLDLSVAKV ATMIKAKTPE
EIRRIFNIVN DFTPEEEAQV REENKWCEDA
```

A. Pro-Skp1:
H-IFNIV($^{13}C_5$,^{15}N)NDFT**P**EEEAQV($^{13}C_5$,^{15}N)R-OH

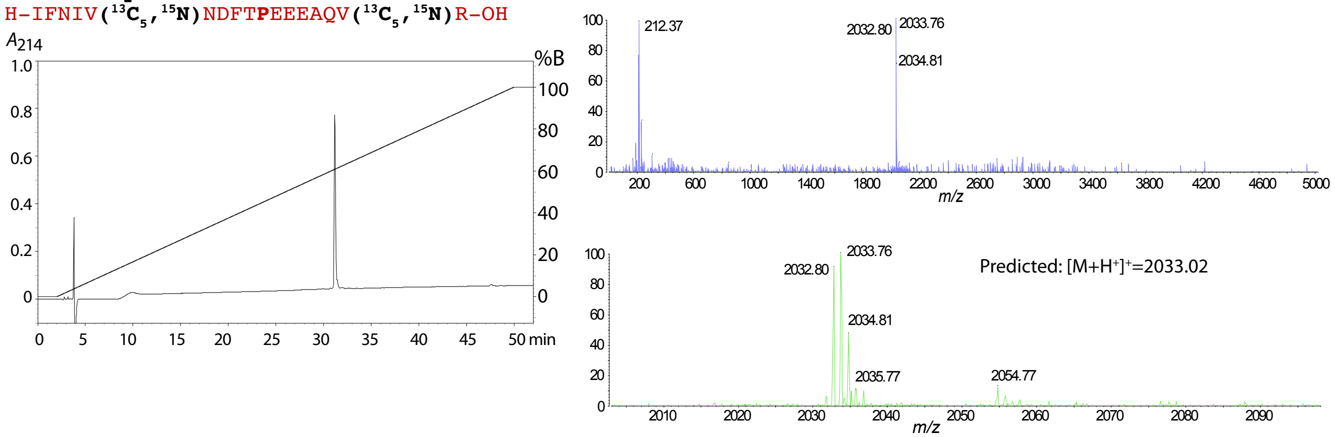

B. Hyp-Skp1:
H-IFNIV($^{13}C_5$,^{15}N)NDFT**P(OH)**EEEAQV($^{13}C_5$,^{15}N)R-OH

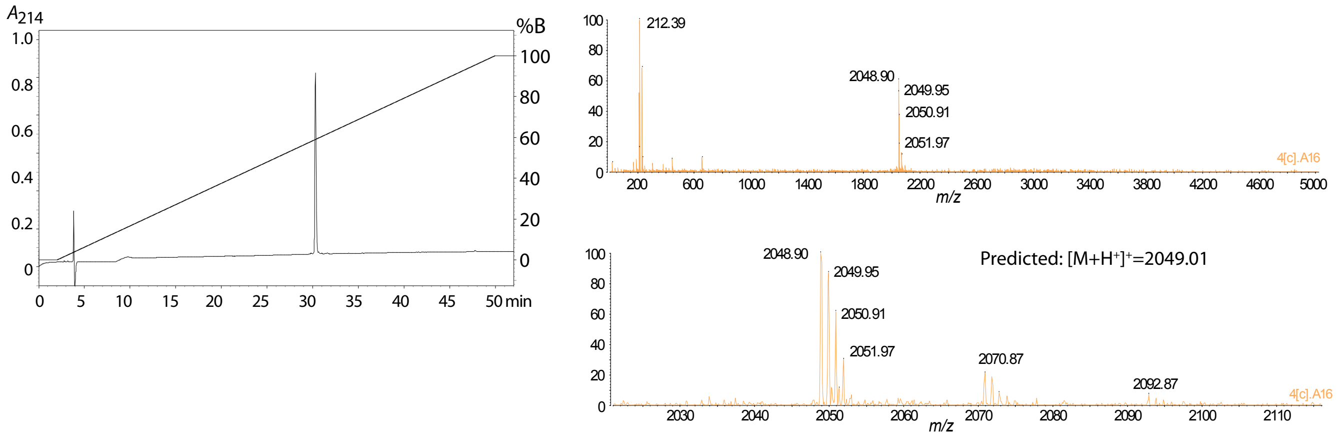

C. GlcNAc-Skp1:
H-IFNIV($^{13}C_5$,^{15}N)NDFT**P(O-α-D-GlcNAc)**EEEAQV($^{13}C_5$,^{15}N)R-OH

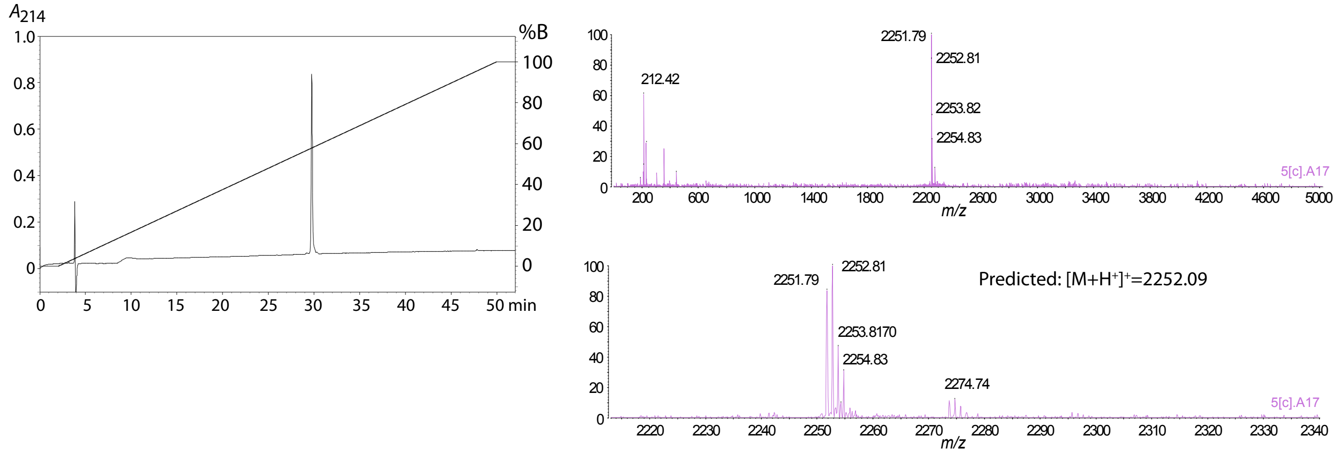

Figure S12. nLC/MS analysis of Skp1 modification status.
Skp1-SF was FLAG-IPed from manually lysed out intracellular PGTaΔ parasites cultured at ambient O_2, released with high pH (Fig. 2C), and digested with endo Lys-C/trypsin. The peptide preparation was supplemented with known amounts of all 3 synthetic peptides possessing heavy isotopes (Fig. S11) as internal standards. Peptides were recovered on C18 zip-tips and analyzed by nLC-MS using a C18 column and an Orbitrap detector operated in positive ion mode. Representative data from a single trial are shown. *A*, expected *m/z* values for native and heavy peptides. *B*, total ion current over a nLC run, and extracted ion chromatograms for each of the 3 possible peptide isoforms in their native and heavy forms. *C*, zoomed in mass spectra for each of the 3 possible peptide isoform pairs (native and synthetic).

A Expected m/z values:

	native peptides	heavy peptides
Pro-peptide	$[M+2H^+]^{+2}$ = 1011.00	$[M+2H^+]^{+2}$ = 1017.01
Hyp-peptide	$[M+2H^+]^{+2}$ = 1019.00	$[M+2H^+]^{+2}$ = 1025.01
GlcNAc-Hyp-peptide	$[M+2H^+]^{+2}$ = 1120.52	$[M+2H^+]^{+2}$ = 1126.55

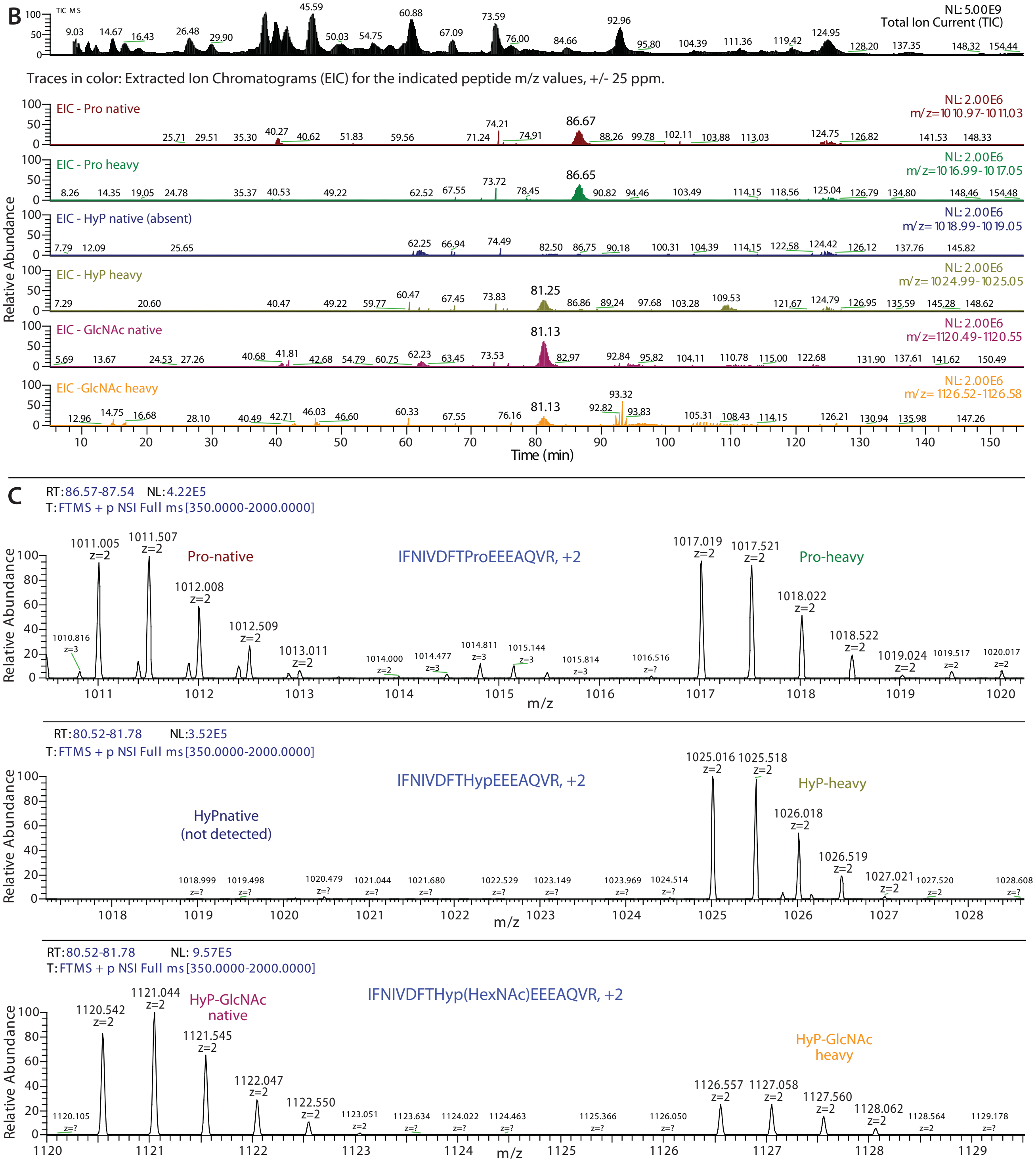

Supplement: Supporting Information [file mmc1.pdf]
